# The Scandinavian Neurotrauma Committee 2026 guidelines for initial management of minimal, mild and moderate head injuries in adults: a systematic review, meta-analysis and modified Delphi consensus study

**DOI:** 10.1016/j.lanepe.2026.101911

**Published:** 2026-11-07

**Authors:** Li Jin Yang, Johan Undén, Alexander Fletcher-Sandersjöö, Jussi P. Posti, Karoline Skogen, Mads Aarhus, Ramona Åstrand, Eric P. Thelin, Scandinavian Neurotrauma Committee, Johan Ljungqvist, Anders Christian Feyling, David Nelson, András Buki, Mads Aarhus, Tor Brommeland, Ramona Åstrand, Johan Undén, Niklas Marklund, Teemu Luoto, Olga Calcagnile, Jussi Posti, Eric Thelin, Elham Rostami, Fredrik Ginstman, Karoline Skogen, Shirin Kordasti, Fredrick Wickbom, Alexander Fletcher-Sandersjöö, Kirsten Møller

**Affiliations:** a Department of Emergency Medicine, Stockholm South General Hospital, Stockholm, Sweden; b Department of Clinical Science and Education Södersjukhuset, Karolinska Institutet, Stockholm, Sweden; c Department of Operation and Intensive Care, Halland Hospital Halmstad, Region Halland, Halmstad, Sweden; d Department of Clinical Sciences, Lund University, Lund, Sweden; e Department of Neurosurgery, Karolinska University Hospital, Stockholm, Sweden; f Department of Clinical Neuroscience, Karolinska Institutet, Stockholm, Sweden; g Neurocenter, Department of Neurosurgery and Turku Brain Injury Center, Turku University Hospital, Turku, Finland; h Department of Clinical Neurosciences, Neurosurgery, University of Turku, Turku, Finland; i Department of Radiology and Nuclear Medicine, Oslo University Hospitals, Oslo, Norway; j Department of Neurosurgery, Oslo University Hospital, Oslo, Norway; k Faculty of Medicine, University of Oslo, Oslo, Norway; l Department of Neurosurgery, Rigshospitalet, Copenhagen University Hospital, Copenhagen, Denmark; m Medical Unit Neurology, Karolinska University Hospital, Stockholm, Sweden

**Keywords:** Traumatic brain injury, Emergency medicine, Head injury, Intracranial lesion, Neurosurgery

## Abstract

**Background:**

Several clinical guidelines exist to identify intracranial injury (ICI) after head injury. The Scandinavian Neurotrauma Committee (SNC) published evidence- and consensus-based guidelines in 2000, updated in 2013. Since then, demographic changes, including an ageing population and increased use of antithrombotic therapy have prompted further revision. The aim of this study is to provide a clinically relevant update to the SNC guidelines for the initial management of adults with minimal, mild, or moderate head injury.

**Methods:**

A PRISMA-compliant systematic review and meta-analysis addressed population, exposure, comparator, and outcome questions on which emergency department patients with minimal or mild head injury require head computed tomography (CT), direct discharge, in-hospital observation, or repeat CT. Evidence was graded using the GRADE approach. Recommendations were developed through a modified Delphi process with stakeholder review.

**Findings:**

Twenty-nine stakeholders participated in developing a seven-point recommendation set and updated management flowcharts. Key changes include reduced emphasis on routine CT in patients receiving direct oral anticoagulants, incorporation of glial fibrillary acidic protein and ubiquitin C-terminal hydrolase L1 biomarkers, and use of structured observation as an alternative to CT in selected risk groups.

**Interpretation:**

The updated guidelines support risk-adapted imaging and observation strategies that may reduce unnecessary CT scans and hospital admissions, although further validation of safety and effectiveness is needed.

**Funding:**

No specific funding.

**Research in context**

**Evidence before this study:**

The Scandinavian Neurotrauma Committee published guidelines for the initial management of minimal, mild, and moderate head injury in 2000 and updated them in 2013. For the present update, we searched MEDLINE and Embase for English-language studies published from August 2012 to April 2024. We examined clinical predictors of traumatic intracranial injury and acute neurosurgical intervention in adults presenting within 24 h of minimal or mild head injury. Existing evidence was derived predominantly from observational cohorts with heterogeneous populations, imaging practices, predictor definitions, and outcome definitions. Important uncertainties remained regarding patients receiving direct oral anticoagulants, the role of blood biomarkers, the safety of observation as an alternative to immediate imaging, and the management of older patients.

**Added value of this study:**

This study combines an updated systematic review and random-effects meta-analysis with assessment of the certainty of evidence using GRADE and a Nordic multidisciplinary modified Delphi process. The updated evidence base included 110 studies evaluating traumatic intracranial injury and 33 studies evaluating acute neurosurgical intervention. Twenty-nine stakeholders participated in the consensus process. The resulting recommendations distinguish the certainty of the underlying evidence from the level of stakeholder agreement and identify recommendations based partly or predominantly on expert consensus. The updated clinical pathways provide separate options according to local biomarker availability and integrate computed tomography, structured neurological observation, hospital admission, and discharge into a risk-adapted framework. They also introduce a combination-based approach to antithrombotic treatment rather than treating exposure to an anticoagulant in isolation as an automatic indication for computed tomography.

**Implications of all the available evidence:**

The updated guidelines provide a structured framework for the initial management of adult head injury in Nordic emergency-care settings. They aim to support more consistent decision making and more selective use of computed tomography and hospital observation, while maintaining escalation pathways for patients at increased risk of clinically important traumatic intracranial injury. However, the certainty of evidence is low or very low for several recommendations, and consensus does not independently establish their safety or effectiveness. Implementation should therefore be adapted to local imaging capacity, observation resources, biomarker availability, transfer arrangements, and opportunities for reliable follow-up. Prospective external validation is planned to assess missed or delayed traumatic intracranial injury, acute neurosurgical intervention, and resource use.

## Introduction

Suspected traumatic brain injury (TBI) is one of the most common causes of emergency department (ED) visits, with an estimate of 60 million cases globally every year.^1^ In the emergency setting, patients present with head injury rather than confirmed TBI, as TBI is established only following clinical assessment or imaging. The Scandinavian Neurotrauma Committee (SNC) classifies ED admitted head injury into minimal, mild, and moderate categories using an adaptation of the Head Injury Severity Scale.^2^ In the Nordic countries, most head trauma patients present with mild head injury.^3^ In the case of a TBI, its severity is traditionally classified as mild, moderate or severe based on the Glasgow Coma Scale (GCS), with mild traumatic brain injury (mTBI) being most common with a low risk of requiring acute neurosurgical or medical intervention at around 1–3% depending on patient selection.^1^, ^4^ Currently, there are a wide variety of clinical decision rules and guidelines available for head injury patients,^5^, ^6^, ^7^, ^8^, ^9^ but there remains no broad international consensus for the optimal ED management of these patients.^10^

The SNC developed evidence and consensus-based guidelines in 2000 with an updated version published in 2013.^3^, ^11^ These guidelines have been validated and shown to reduce computed tomography (CT) scanning in about 30% of patients while maintaining safety.^12^, ^13^, ^14^, ^15^ Since the last update, which included S100B, additional biomarkers have become available in the clinical setting like glial fibrillary acidic protein (GFAP). The head injury population has also changed significantly, with an increase in the elderly population, in whom antithrombotic medication and cognitive decline are common.^16^, ^17^, ^18^ The use of oral anticoagulants has also evolved substantially, with direct oral anticoagulants (DOACs) largely replacing warfarin in many clinical settings.^19^ This trend places a significant burden on healthcare systems, as current recommendations often lead to head CT imaging and hospital admission for observation in a large proportion of patients despite a low likelihood of radiologically detected traumatic intracranial injury (ICI) that requires neurosurgical intervention.^20^, ^21^

The aim of this study was to update the SNC guideline for 2026 and provide full transparency on the methodology and rationale behind its development. These guidelines are intended to be applied in an ED or equivalent setting on adult patients 18 years of age or older with head injury and GCS score 9–15, where there is a reasonable expectation that the patient may benefit from acute diagnostic work-up and potential escalation to neurosurgical or neurointensive care interventions. They are not intended to guide management in patients for whom such escalation is not clinically appropriate due to pre-injury frailty, comorbidity burden, or established treatment-limiting decisions. The intention is to support decision-making regarding imaging, observation, admission and safe discharge, while optimising resource utilisation.

## Methods

The guidelines were developed using a multistep methodology consistent with the process for the preceding version, following the Appraisal of Guidelines for Research and Evaluation (AGREE) framework.^22^ An update to the systematic review performed for the 2013 guidelines was conducted in accordance with the Preferred Reporting Items for Systematic Reviews and Meta-analyses (PRISMA) statement.^23^ The SNC mTBI workgroup then proposed an evidence-based draft for the updated guidelines. For the final stage of guideline development, stakeholders were invited from all relevant medical specialities in the Nordic countries and patient organisations. A modified Delphi meeting was held to establish consensus and refine the proposed guidelines based on expert commentary.^24^ The full workflow process for the guideline update is shown in Fig. 1.

**Fig. 1:**
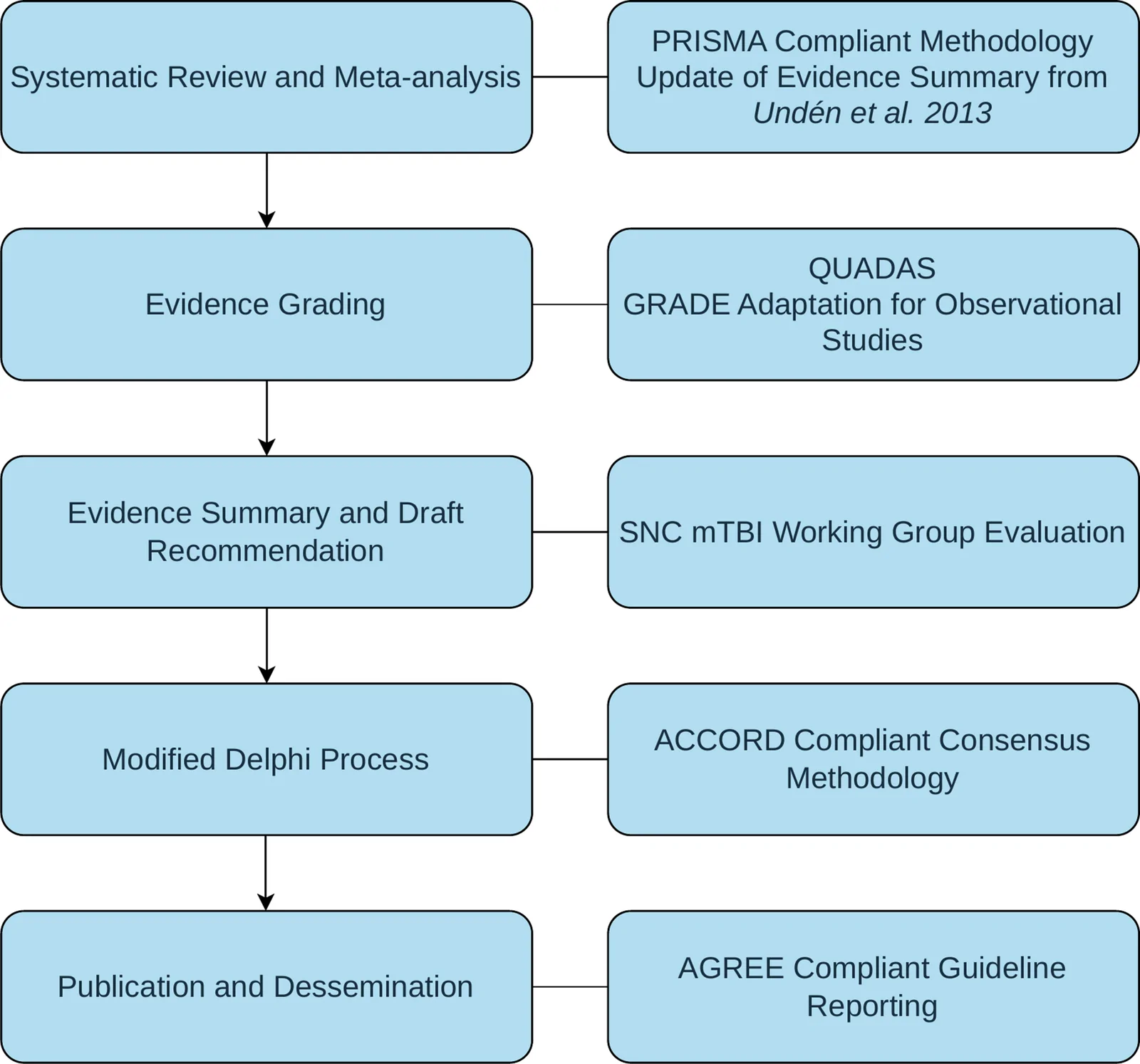
*Flow diagram showing the complete guideline development process*. PRISMA, Preferred Reporting Items for Systematic Reviews and Meta-Analyses, QUADAS, Quality Assessment of Diagnostic Accuracy Studies, GRADE, Grading of Recommendations Assessment, Development, and Evaluation, SNC, Scandinavian Neurotrauma Committee, mTBI, Mild Traumatic Brain Injury, ACCORD, Accurate Consensus Reporting Document, AGREE, Appraisal of Guidelines for Research and Evaluation.

The objective of this work was to update the evidence-based guidelines for the initial 24-h management of adult patients 18 years of age or older presenting to the ED with head injury and GCS score 9–15, specifically to decide which patients are suitable for CT scanning, admission or discharge. The aim was to identify patients with traumatic ICIs that require emergency neurosurgical intervention while minimising unnecessary CT imaging and hospital admissions. We define emergency neurosurgical intervention as surgery performed to prevent neurological deterioration or death from traumatic ICIs, including haematoma evacuation, decompressive craniectomy, intracranial pressure monitoring, or external ventricular drainage. We acknowledge that thresholds for intervention may vary across centres depending on local practice, resources, and clinician judgement in existing literature. Table 1 defines the terminology used in this paper and clarifies their intended context.

**Table 1:** Terminology and definitions.

Term	Definition	Context
Head injury	Any head trauma due to external force	Distinguishes presentation prior to confirmed diagnosis (TBI), classified by the SNC into minimal (no loss of consciousness or neurological deficits), mild and moderate based on GCS score and clinical risk factors
TBI	A head injury that results in a disturbance of normal brain function	Diagnosed after clinical assessment or imaging. Classified into mild, moderate or severe based on GCS score.
ICI	Any traumatic structural brain damage including haemorrhage visible on ED neuroimaging	Imaging (non-contrast head CT) based outcome
Neurosurgical intervention	Acute invasive procedure performed to prevent further neurological deterioration or death from ICIs	Key outcome and used to define clinically significant ICI subset

TBI, Traumatic Brain Injury; SNC, Scandinavian Neurotrauma Committee; GCS, Glasgow Coma Scale; ICI, Intracranial Injury.

As an extension to the systematic review conducted for the 2013 guideline, updated searches were performed targeting the following population, exposure, comparison and outcome questions:

Question 1, addressing: “Which adult ED patients with minimal or mild head injury require a head CT, and which can be safely discharged without imaging?”

Population: Adult mild head injury or mTBI patients at the ED within 24-h from injury.

Exposure: Predictive variable available from the ED prior to a radiological examination.

Comparator: Absence of the exposure predictive variable.

Outcome: CT-verified acute traumatic ICI.

Question 2, addressing: “Which adult ED patients with minimal or mild head injury require in-hospital observation and/or a repeat head CT?”

Population: Adult mild head injury or mTBI patients at the ED within 24-h from injury.

Exposure: Predictive variable available from the ED prior to a radiological examination.

Comparator: Absence of the exposure predictive variable.

Outcome: acute neurosurgical intervention.

The two search strategies applied were adapted from the systematic review conducted in conjunction with the 2013 SNC guideline development and applied Medical Subject Headings (MeSH) terms and keywords adjacent to “brain injury”, “management”, “hospitalisation” and “observation”. The search was limited to publications in the English language. Searches were conducted in the MEDLINE and EMBASE databases from August 2012, when the last guidelines were draughted, to April 2024. The full search strategies are available in the Appendix.

Title screening was conducted by one author (LJY) and abstract and full text screening was conducted by two authors (LJY and EPT). Discrepancies during the screening process were resolved through discussion and one author was available for conflict resolution if consensus could not be reached (JU). Data were extracted by one author (LJY) and checked by two others (EPT and JU). Evidentiary tables were constructed including quality grading through Centre for Evidence-Based Medicine (CEBM) criteria^25^ as well as the Quality Assessment of Diagnostic Accuracy Studies (QUADAS) tool,^26^ and the summary level of evidence was evaluated using the Grading of Recommendations Assessment, Development and Evaluation (GRADE) approach.^27^ A meta-analysis was conducted based on data extracted for each predictive variable for the two outcomes of interest and integrated with the dataset from the original 2013 review. For each clinical predictor, data on events and totals with the predictor present and absent were extracted. Data is presented as forest plots for calculated odds ratios (OR) as well as funnel plots for assessment of publication bias were generated using the web-based platform MetaAnalysisOnline,^28^ applying a random effects model using the DerSimonian-Laird model as a heterogeneity estimator.^29^, ^30^ Statistical heterogeneity was assessed using I^2^ and τ^2^. I^2^ values of approximately 0–25%, 26–50%, 51–75%, and above 75% were interpreted as low, moderate, substantial, and considerable heterogeneity, respectively. Studies with zero events in both exposure groups were not estimable for relative effects and were excluded from the relevant synthesis. A sensitivity analysis using the leave-one-out method was performed for each predictor and outcome using OpenMeta Analyst (based on the metafor package 1.6.0 in R version 3.2.4).^31^ The review was preregistered in the Open Science Framework database (DOI: 10.17605/OSF.IO/G3QB4), selected due to ongoing issues with PROSPERO phasing out its peer review process during the study period and for data file archival and dissemination purposes.^32^ The candidate predictors included in the meta-analysis were partially inherited from the 2013 review and selected based on the findings from the updated screening. Where a study reported several strata for the same predictor, strata were combined where clinically appropriate to create a single exposed group, while retaining a non-overlapping reference group and avoiding double-counting of participants. Variable selection and harmonisation procedures are fully reported in the Appendix.

The authors LJY, EPT, JU, and AFS reviewed the evidentiary tables, meta-analysis data, and GRADE-based evidence grading prior to draughting the updated recommendations. The draft was evaluated by all members of the SNC mTBI workgroup (all co-authors) before distribution to stakeholders in the form of a pre-Delphi package with instructions for the Delphi meeting.

To address differences of opinion prior to implementation, a modified Delphi method was used.^24^, ^33^ The SNC mTBI workgroup recruited stakeholders with proximity to acute management of TBI through specialist organisations in emergency medicine, neurosurgery, neurology, intensive care, general and trauma surgery, geriatric medicine, rehabilitation medicine and patient organisations in all Nordic countries. No financial incentive was provided to stakeholder participants. Stakeholder recruitment was designed to be maximally inclusive rather than based on predetermined quotas. No upper limit was placed on the total number of participants. Dissemination was delegated to these organisations and networks rather than restricted to a central list of named invitees, thus the total number of individuals who received or had access to the invitation could not be determined. The stakeholders used a seven-point Likert-scale to provide individual, anonymised responses and the aggregated results were used to determine the level of consensus for each of the presented recommendations. Over 75% of respondents scoring ≥5 was defined *a priori* as consensus in favour of the recommendation and ≤3 as consensus against. The modified Delphi meeting was conducted in conjunction with the European Neurotrauma Summit in Lund, Sweden on the 11th of November 2025. Participation was possible on-site at Skåne University Hospital Lund or through video conferencing. The SNC mTBI workgroup members were non-voting moderators during the modified Delphi meeting. Stakeholders were encouraged to provide both oral and written feedback on all aspects of the recommendations, weighing the risk versus benefit aspects of the update and obstacles to implementation in a Nordic context. Multiple rounds of voting were planned if consensus could not be reached. A final reassessment of the summary result of the modified Delphi process was made within the workgroup before approval for dissemination.

### Role of the funding source

No funding has been received for the undertaking of this research project.

## Ethics approval

Ethics approval was not required for either the systematic review and meta-analysis of published data or the modified Delphi consensus process involving professional stakeholders. Participation in the Delphi process was voluntary, and no patient data or sensitive personal information were collected.

## Results

The complete search, screening process and synthesis of literature with the 2013 systematic review for the two clinical questions are presented as PRISMA flowcharts in Figs. 2 and 3. For the first clinical question, a total of 110 studies were included combining 38 new studies with 72 studies from the previous review. To address the risk of multiple comparison groups and double counting participants, publications were assessed for overlapping cohorts and populations during the screening process, resulting in a total of five studies excluded on the basis of duplicated cohorts (Fig. 2). For the second clinical question, a total of 33 studies were included, combining 12 studies with 21 studies from the previous review.

**Fig. 2:**
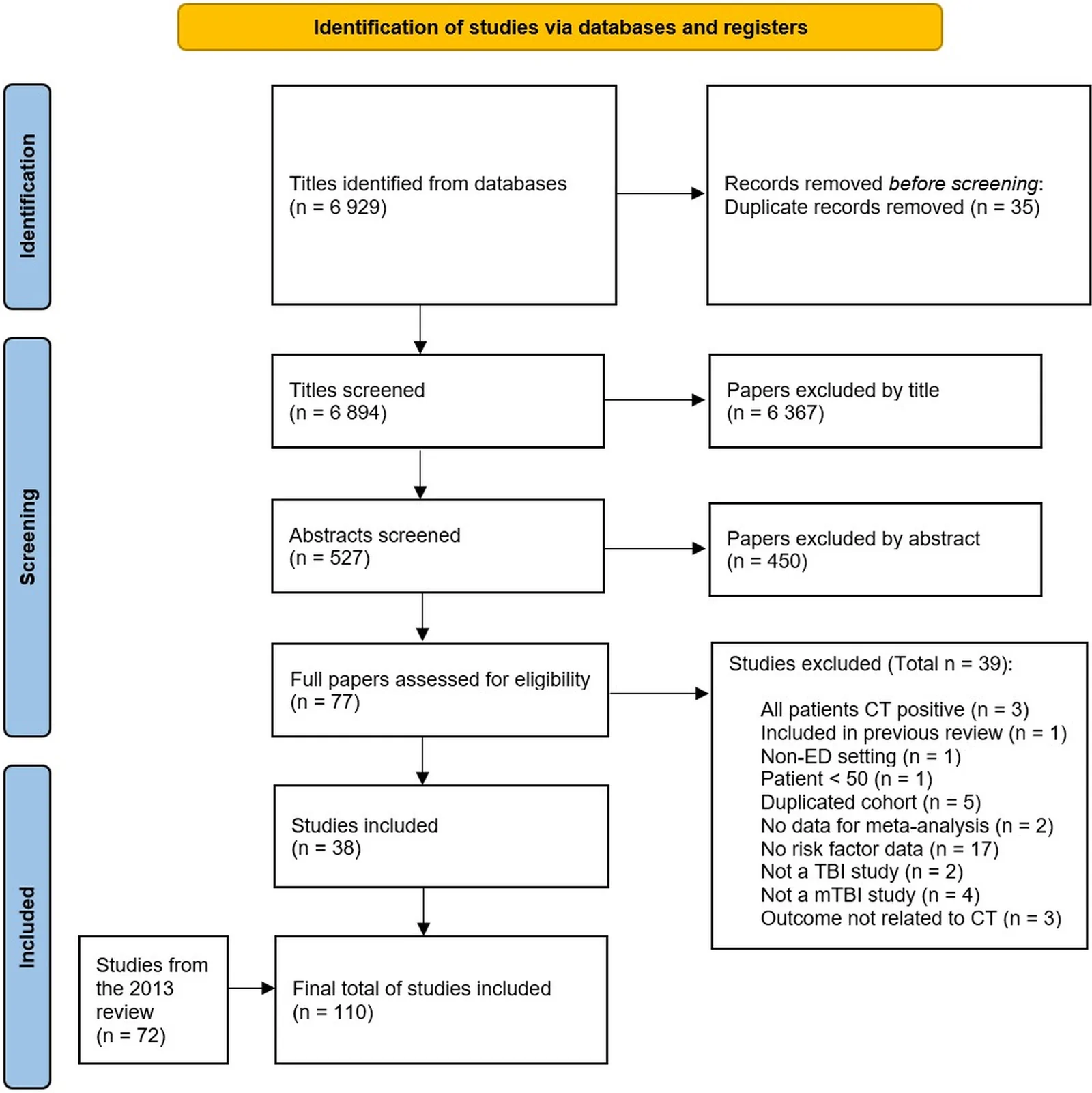
*Preferred Reporting Items for Systematic Reviews and Meta-Analyses (PRISMA) flowchart showing the search and screening process for the clinical question: “Which adult emergency department patients with minimal or mild head injury need a head computed tomography and which patients may be directly discharged?”*. CT, Computed Tomography, ED, Emergency Department, TBI, Traumatic Brain Injury, mTBI, Mild Traumatic Brain Injury.

**Fig. 3:**
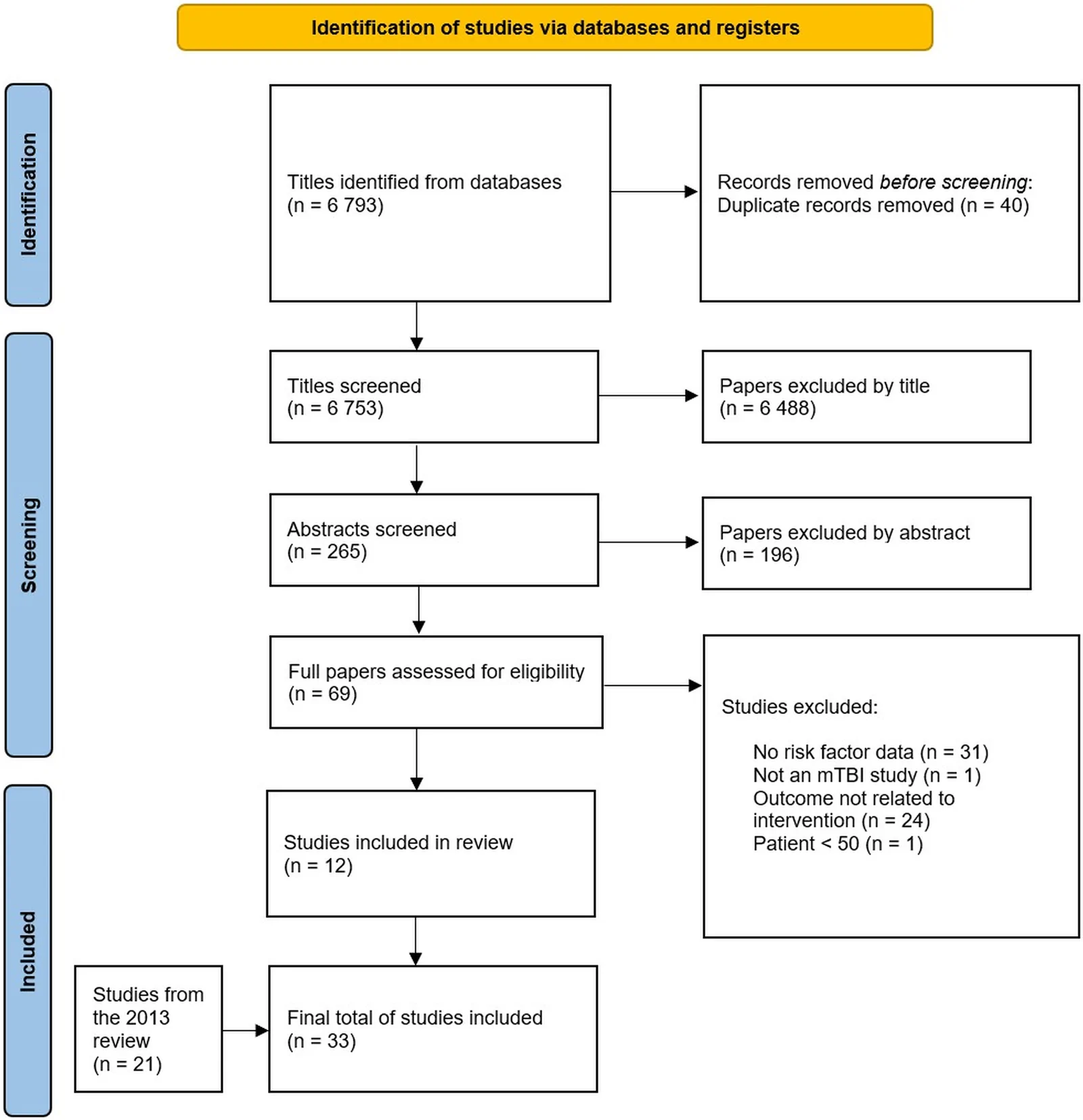
*Preferred Reporting Items for Systematic Reviews and Meta-Analyses (PRISMA) flowchart showing the search and screening process for the clinical question: “Which adult emergency department patients with minimal or mild head injury need in-hospital observation and/or a repeat head computed tomography?”*.

The quality of the studies as evaluated by CEBM were predominantly at level 3–4, at an intermediate tier due to the observational nature of a majority of the studies (Appendix). When applying QUADAS to evaluate the included studies, for the first clinical question a majority of studies presented some concerns for overall risk of bias, while for the second clinical question approximately two-thirds of the studies included presented a high risk of bias (Appendix), with the highly varied patient selection criteria being a significant contributor to bias. For the first clinical question with outcome defined as radiologically verified traumatic ICI, a total of 19 predictive variables were available for data synthesis (Fig. 4). For the second clinical question with outcome defined as neurosurgical intervention, six predictive variables were available for data synthesis (Fig. 5).

**Fig. 4:**
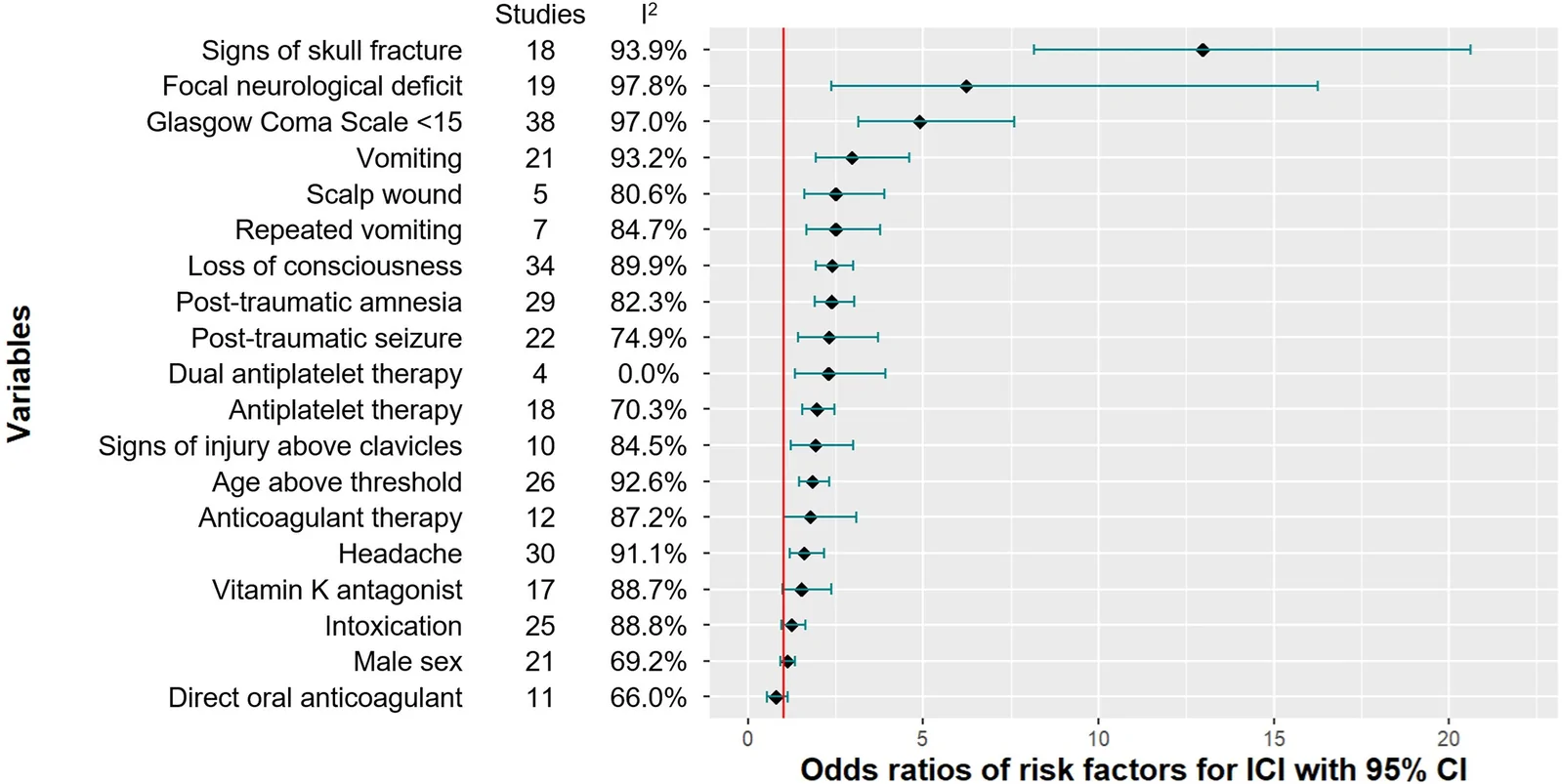
*Forest plot summarising the findings from all meta-analyses performed for individual predictive factors for traumatic ICI*. I^2^, percentage of variability in effect estimates attributable to between-study heterogeneity rather than sampling error: 0–25%: low heterogeneity, 25–50%, moderate heterogeneity, 50–75%, substantial heterogeneity, 75–100%, considerable heterogeneity. ICI, Intracranial Injury, CI, Confidence Interval.

**Fig. 5:**
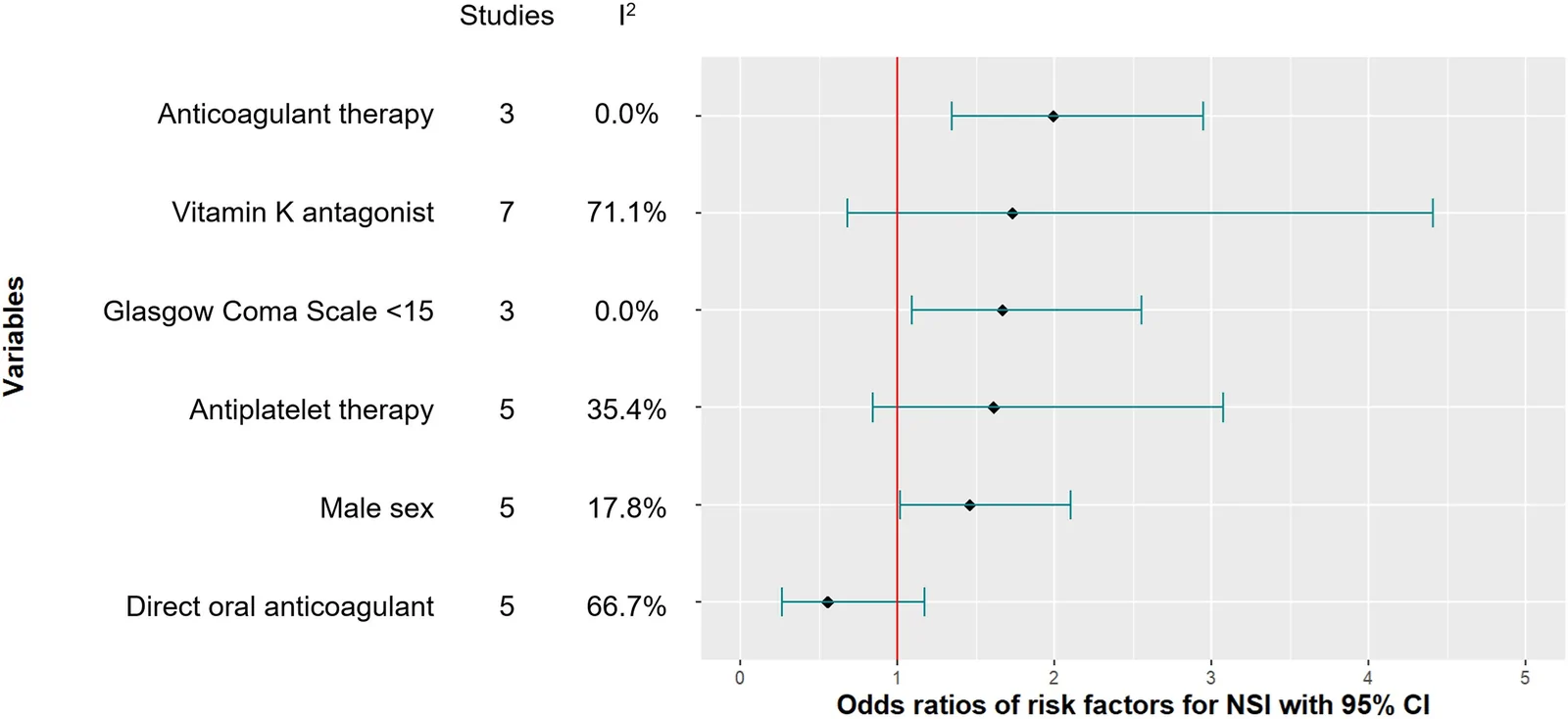
*Forest plot summarising the findings from all meta-analyses performed for individual predictive factors for neurosurgical intervention*. I^2^, percentage of variability in effect estimates attributable to between-study heterogeneity rather than sampling error: 0–25%: low heterogeneity, 25–50%, moderate heterogeneity, 50–75%, substantial heterogeneity, 75–100%, considerable heterogeneity. NSI, Neurosurgical Intervention, CI, Confidence Interval.

Forest plots, funnel plots and leave-one-out sensitivity analyses for all individual predictive variables are available in the Appendix. The strongest predictor for traumatic ICI was signs of skull fracture with an OR of 12.98 (95% confidence interval, CI 8.16–20.63), summarised from 18 studies. The strongest predictor for neurosurgical intervention was any anticoagulation with an OR of 1.87 (95% CI 1.32–2.64). The pooled analysis did not demonstrate a statistically significant association between DOAC use and either outcome (OR and 95% CI of 0.79, 0.55–1.14 and 0.58, 0.28–1.18 respectively).

The GRADE system was utilised to evaluate the factors that impact the level of evidence from the systematic review addressing the outcomes of interest.^27^ These factors include the availability of data, selection bias, inconsistencies in methodology and study design. Additional analysis was performed for each risk factor based on the subgroup of literature available on a case-by-case basis. Overall level of evidence is moderate to low. The complete GRADE tables are available in the Appendix. A seven-point list of recommendations was draughted to reflect key points from the literature review (Table 2). The final guideline is also provided as flowcharts with variations for settings with or without biomarker availability (Fig. 6 and Appendix).

**Table 2:** Recommendations draughted based on evidence grading and workgroup consensus.

	Modified Delphi recommendation text	GRADE level of evidence certainty	Basis	Delphi agreement level	Statement status
Recommendation 1	We recommend that adult patients with mild head injury (GCS score 14–15) with an intraventricular shunt or on dual antiplatelet therapy should undergo a head CT or undergo an observation period.	Very low level of evidence	Evidence- informed, consensus-based and retained prior recommendation	100%	Good-practice statement based on expert opinion
Recommendation 2	We suggest that adult patients with mild head injury should undergo head CT or structured observation if they have either: (1) a post-traumatic GCS score of 14 or (2) a GCS score of 15 together with at least two of the following factors: pre-injury anticoagulant therapy, pre-injury antiplatelet monotherapy, suspected loss of consciousness, repeated vomiting, scalp wound, or age ≥65 years.	Moderate/low level of evidence	Evidence-informed and consensus-based recommendation	75.8%	Consensus-based recommendation based on expert opinion
Recommendation 3	We recommend that adult patients with moderate head injury (GCS score 9–13), or mild head injury (GCS score 14–15) with post-traumatic seizure, new focal neurological deficits, or signs of depressed or basilar skull fractures should undergo a head CT and undergo admission regardless of the result.	Moderate/low level of evidence	Evidence- informed and retained prior recommendation	100%	Consensus-based recommendation based on expert opinion
Recommendation 4	We recommend that adult patients with mild head injury with GCS score 14, or GCS score 15 with at least two of: pre-injury anticoagulation therapy, pre-injury antiplatelet monotherapy, suspected loss of consciousness, repeated vomiting, or scalp wound can be subjected to biomarker testing with S100B or GFAP/UCH-L1 when available. For S100B: if the patient is ≤ 65 years of age without presence of extracranial injury and ≤6 h from time of injury. For GFAP/UCH-L1 or GFAP in isolation: if the patient is ≤ 65 years of age and ≤12 h from time of injury. Biomarker value below threshold rules out the need for an acute head CT.	Moderate/low level of evidence	Consensus-based and retained prior recommendation	86.2%	Consensus-based recommendation based on expert opinion
Recommendation 5	We suggest that adult mild head injury patients may undergo observation as an alternative to an acute head CT. Observation may be done in-hospital or in the emergency department, depending on local routines. We recommend checking for decreased level of consciousness (GCS score) and unilateral extremity weakness (basic neurological exam) every 15 min during the first 3 h post-trauma and every 30 min following this. Observation frequency can be tailored to patient status (i.e. stable patients may need less frequent observation). If a CT has been performed and the patient is being observed, a routine repeated head CT should not be performed unless there is deterioration in GCS score ≥2 points or new/progressive neurological deficits (unilateral motor or sensory dysfunction, dysphasia or visual disturbance). The recommended minimum observation time is 6 h post-trauma.	Very low level of evidence	Consensus-based recommendation	75.9%	Good-practice statement based on expert opinion
Recommendation 6	We recommend that all adult patients with minimal, mild and moderate head injury should receive written information prior to discharge. Patients with worrying or worsening symptoms (other than typical post-injury issues such as mild headache and nausea) should not be discharged. Patients should be discharged at their premorbid neurological and functional baseline and into an appropriate level of supervision or care (see attached discharge instructions).	Very low level of evidence	Consensus-based and retained prior recommendation	89.6%	Good-practice statement based on expert opinion
Recommendation 7 Implementation statement	We recommend that all adult patients presenting to an ED with minimal, mild and moderate head injury and are candidates for emergency neurosurgical and medical interventions should be managed with the updated 2026 Scandinavian Neurotrauma Committee recommendations (see attached flowcharts and help sheets), applying a version that is appropriate based on local availability of biomarkers.	Very low level of evidence	Consensus-based implementation statement	86.2%	Implementation statement based on expert opinion

GCS, Glasgow Coma Scale; CT, Computed Tomography; GFAP, Glial Fibrillary Acidic Protein; UCH-L1, Ubiquitin Carboxy-terminal Hydrolase L1. The flowchart for standard management without biomarkers is presented in Fig. 6. Flowcharts for initial management with biomarkers and discharge instructions are available in Appendix.

**Fig. 6:**
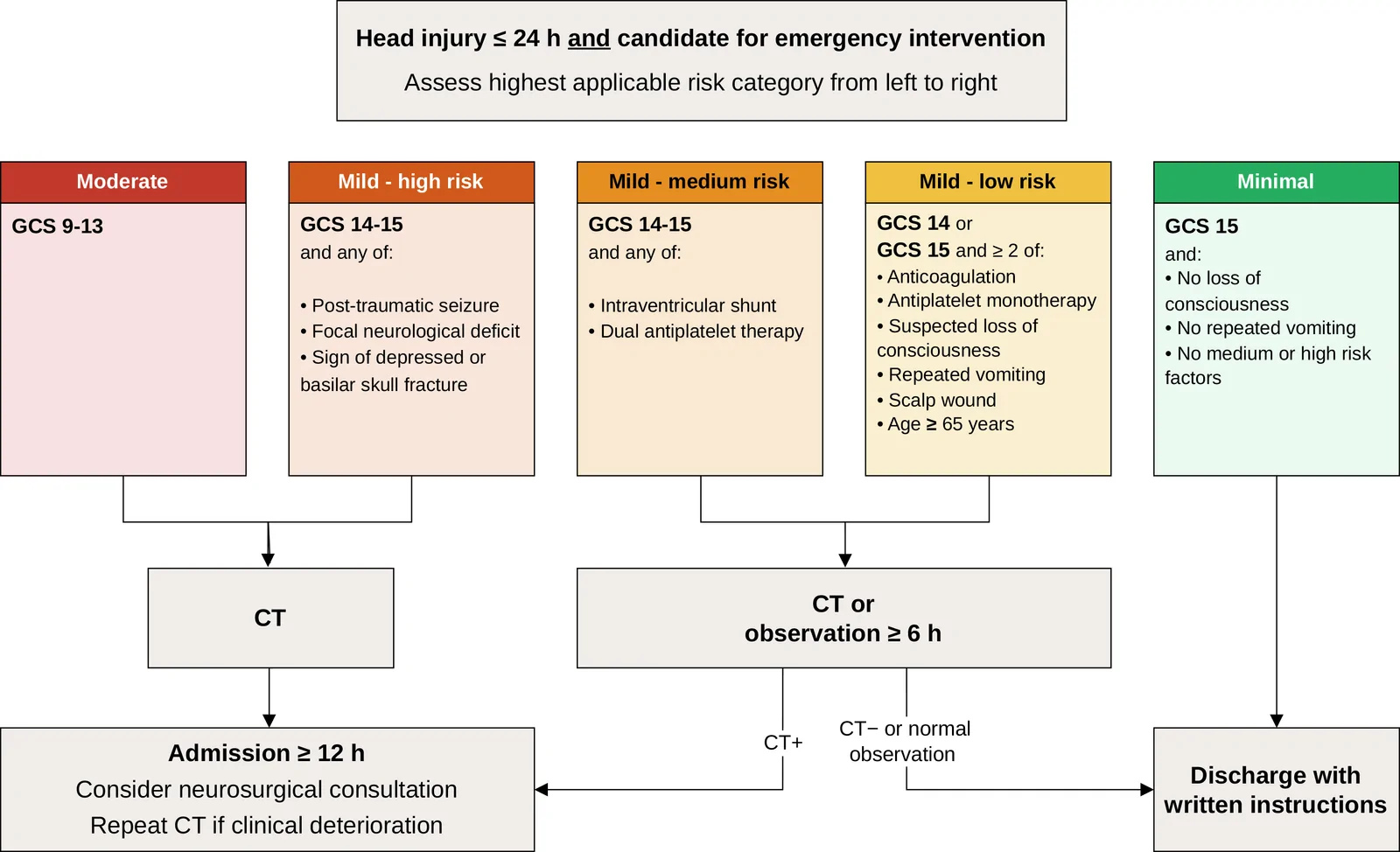
*Guidelines flowchart for the management of adult minimal, mild and moderate head injury patients ≥18 years of age in settings without biomarker availability*. GCS, Glasgow Coma Scale, CT, Computed Tomography. Help sheet for the flowchart available in the Appendix.

A total of 29 stakeholders participated in the modified Delphi process (see Appendix). The largest constituent of stakeholders were representatives from neurosurgery at 24.1%, followed by emergency medicine at 20.7%, with general and trauma surgery and neurointensive care at shared third at 17.2%. Seven of the stakeholders were non-workgroup members of the SNC. Of the four Nordic countries represented, Sweden had a majority of stakeholders at 58.6%, followed by Norway at 17.2%, Finland at 13.8% and Denmark at 10.3%. Nine of the 29 stakeholders were female (31%). Consensus was reached for all recommendations during the first round of the Delphi process. Recommendations 1 and 3 had the highest level of agreement at 100%, while recommendations 2 and 5 had the lowest at 75.8% and 75.9% respectively.

Rationale for Recommendation 1: Four studies from our systematic review indicate that pre-injury dual antiplatelet therapy is predictive of traumatic ICI in mTBI patients (OR 2.30, 95% CI 1.35–3.92).^34^, ^35^, ^36^, ^37^ OR for traumatic intracranial haemorrhage in older mTBI patients on dual antiplatelet therapy is also reported in a 2025 study at 1.02 (95% CI 0.54–1.78), and though not statistically significant, is higher than the reported ORs for DOAC, vitamin K antagonists, and antiplatelet single therapy within the study setting.^38^ Despite limited data, these patients remain a higher-risk subgroup. We recommend that either a head CT or a period of observation are viable management options in stable patients with no deterioration of GCS score from baseline and absence of symptoms or no symptom progression (see rationale for Recommendation 5).

Rationale for Recommendation 2: We recommend significant changes to the management of patients on antithrombotic medication. Previous recommendations were influenced by a single-centre study suggesting higher rates of delayed intracranial haemorrhage (defined as ICI findings in repeated head CT performed in patients with an initial normal head CT) in anticoagulated patients on warfarin.^39^ However, recent Nordic studies in cohorts after the introduction of DOACs demonstrate extremely low rates of clinically relevant delayed haemorrhage, with no reported cases requiring neurosurgical intervention and an estimated numbers needed to scan of 1500 to detect a single delayed haemorrhage within the first 24 h after injury.^21^, ^40^, ^41^, ^42^ Though these studies were generally observational with no follow-up CT performed if no clinical deterioration occurred, findings are consistent across studies. A two-year observational study conducted in Sweden also demonstrated a 0.08% incidence of clinically relevant delayed haemorrhages (95% CI 0.01–0.3%), with no cases resulting in neurosurgery, demonstrating a very limited clinical benefit of admitting anticoagulated patients with negative initial head CT for further observation.^20^ In the present meta-analysis, neither vitamin K antagonist nor direct oral anticoagulant use demonstrated a statistically significant unadjusted association with traumatic intracranial injury or acute neurosurgical intervention. However, the analyses were based principally on aggregate event counts and could not consistently adjust for age, frailty, injury mechanism, GCS score, concomitant medication, or other potential confounders. Two included cohort studies provide supportive adjusted evidence. Antithrombotic treatment was not independently associated with intracranial haemorrhage in a cohort of 1846 adults with mTBI, although differential CT use between medication groups creates a risk of verification bias. In a separate cohort of 4850 adults with head trauma, oral anticoagulation was associated with intracranial haemorrhage in the unadjusted analysis but not after multivariable adjustment, whereas antiplatelet therapy remained independently associated.^34^, ^36^ These findings suggest that the crude association with oral anticoagulation may partly reflect differences in other patient and injury characteristics, but they do not establish absence of anticoagulant-related risk. Recommendation 2 therefore considers anticoagulant therapy as one component of a combination-based assessment rather than as an automatic indication for imaging. A patient with GCS score 15 and anticoagulant therapy as the only identified risk factor does not automatically meet the proposed threshold for CT or structured observation. This is a consensus-based recommendation supported by low-certainty evidence, requires prospective validation, and should not override clinical judgement.

In addition to anticoagulation therapy, the risk factors we have included in this category (antiplatelet monotherapy, suspected loss of consciousness, repeated vomiting, scalp wound and age ≥65 years) were all shown to have an OR in the 1–3 range against ICI. Previous recommendations from the 2013 guidelines have applied a combined risk factor of age ≥65 with antiplatelet therapy where the management options proposed were a minimum of 12 h of observation or head CT, providing a validated reference point. The variable scalp wound was added due to its relatively high OR in our meta-analysis (2.51, 95% CI 1.62–3.90, based on five studies) as well as its utility as a marker of significant impact over the neurocranium and a plausible mechanism for a TBI which is also more specific than legacy predictors such as injury above the clavicle included in the New Orleans CT Head Criteria.^6^ We specifically define scalp wound as a traumatic injury over the eyebrow level requiring management (such as suturing).

Repeated vomiting was defined as more than one episode after the injury. The pooled estimates for vomiting once and repeated vomiting were similar and had overlapping confidence intervals. Retaining repeated vomiting as the operational threshold was therefore a consensus-based choice intended to improve specificity rather than an evidence-established distinction between the two predictors.

Rationale for Recommendation 3: Our recommendations for the management of moderate head injury, and head injury patients with post-traumatic seizure, new focal neurological deficits, or signs of depressed or basilar skull fractures remain unchanged from the 2013 guidelines.

Rationale for Recommendation 4: We retain our previous recommendation for the usage of serum biomarkers as an alternative to head CT to rule out CT-detectable traumatic ICI in selected low-risk patients. In addition to S100B which was included in the 2013 recommendations, we add GFAP in isolation or in combination with UCH-L1 as additional biomarker alternatives. The workgroup references a recent Swedish Health Technology Assessment report summarising current evidence on TBI biomarkers suggesting the potential for biomarker usage to reduce head CT use.^43^ While the cumulative evidence in currently available literature suggests that there may be room to extend the sampling time window for GFAP and UCH-L1 past 12 h post-trauma,^44^ late sampling appears to contribute to a reduction in sensitivity for epidural haemorrhages,^45^ and should be avoided.

Rationale for Recommendation 5: There is currently limited evidence to support any specific observation protocol, and the current SNC recommendation is based on internal consensus that a decreased level of consciousness and new onset unilateral focal neurological deficits, dysphasia or visual disturbance are the most specific findings to warrant a renewed assessment and a repeated head CT in observed patients. We make a good-practice statement that the initial 3 h post-trauma require intensified observation. This recommendation is intended to be considered in non-ED settings (such as residential and care facilities for the elderly) as a basis for refraining from transporting an asymptomatic patient. The specific minimum time recommended for admission is 12 h and observation 6 h, provided the patient is either symptomatically improving or asymptomatic. Additionally, to clarify the intended setting for observation, a centre with CT but no on-site neurosurgery is recommended to observe selected clinically stable patients if images can be reviewed promptly and urgent neurosurgical advice and transfer are available. A centre without CT should not use observation as a substitute for imaging when an immediate CT indication is present as such patients require transfer according to local emergency pathways. Observation without on-site CT is limited to carefully selected low-risk patients only when deterioration can be recognised promptly and transfer can be activated without important clinical delay.

Rationale for Recommendation 6: Discharge instructions were updated to provide a clear distinction between symptoms that may be associated with acute intracranial haemorrhagic progression and post-concussive symptoms. In patients with pre-existing conditions, discharge should be at the individualised premorbid baseline level and into an appropriate level of care.

Rationale for Recommendation 7 (Implementation Statement): Integration of all preceding recommendations into a holistic management flowchart, with alternative versions for biomarker usage, help sheets to clarify definitions and guide clinically sound implementation.

## Discussion

This report presents the 2026 update of the SNC guidelines for the initial management of minimal, mild, and moderate head injury in adults. The guideline is intended for patients in whom the result of acute diagnostic assessment could reasonably alter management. In patients with advanced frailty, substantial comorbidity, or established treatment limitations, decisions concerning management should be individualised according to goals of care and patient preferences. The guideline should not be used to deny diagnostic evaluation solely on the basis of age, frailty, or disability. This restriction reflects two distinct scenarios: patients with minimal, non-surgical traumatic ICIs who are clinically stable and without neurological deterioration, and patients who would not be candidates for emergency intervention regardless of CT findings, for instance due to frailty, advanced comorbidity, or goals-of-care decisions. In both groups, CT-based screening is unlikely to alter management. This outcome-oriented approach is consistent with broader trends in emergency medicine toward clinically meaningful triage, which also aligns with ongoing global initiatives encouraging reduction of low-value healthcare.^46^

The updated recommendations reflect several important shifts in the position of the SNC on TBI management. Firstly, while the SNC 2013 guideline recommended routine admission and repeat imaging in anticoagulated patients, accumulating evidence suggests that the risk of clinically significant delayed haemorrhage is lower than previously estimated. The present guideline therefore emphasises individualised risk stratification and allows observation as an alternative to immediate CT imaging. This approach is also comparable to the stance taken in the National Institute for Health and Care Excellence (NICE) guideline where antithrombotic medication is stratified as a risk factor where an acute head CT should be considered but not fully mandated.^5^ One implication of our guideline is that patients on antithrombotic medication under 65 years of age with minimal or mild head injury are no longer required to undergo a CT at all. Though no stakeholder specifically addressed this during the modified Delphi process, the workgroup acknowledges that the update contributes to a decrease in sensitivity as a trade-off to the increase in specificity, which in clinical scenarios may translate to a risk of missing minimal non-neurosurgical traumatic ICIs.

Secondly, the most substantial difference between the SNC 2026 guidelines and current international recommendations concerns DOACs. Our meta-analysis found that DOAC use was not a statistically significant predictor of either ICI (OR 0.79, 95% CI 0.55–1.14) or neurosurgical intervention (OR 0.58, 95% CI 0.28–1.18). The point estimates suggesting a potentially protective effect likely reflect confounding rather than a true biological effect, as DOAC users may represent a population with lower-energy trauma mechanisms and fewer high-risk comorbidities. Nevertheless, the consistent absence of a significant association across studies informed the consensus-based decision to consider DOAC exposure within a combination-based assessment rather than as an automatic CT indication. The safety of this approach remains to be established prospectively. To our knowledge, SNC 2026 is the first major clinical guideline to formally de-escalate DOAC from an independent indication for CT, differentiating from NICE (2023) and other current recommendations that retain anticoagulation as an indication for imaging consideration.

Thirdly, the updated guideline incorporates additional biomarkers GFAP and UCH-L1, as alternatives to rule out the need of CT imaging in selected patient populations. Biomarker-guided strategies have the potential to reduce CT utilisation, particularly among low and intermediate-risk head injury patients. However, variation in biomarker availability and limited clinical experience across healthcare systems remain important considerations. To address this, parallel management guidelines were developed to support implementation in settings both with and without biomarker availability. Also, UCH-L1 seems to add limited clinical utility. A guideline with only GFAP included was therefore presented, although we acknowledge the current lack of GFAP-only analysis kits. To our knowledge, this is the first European clinical guideline to formally incorporate GFAP and UCH-L1 as alternatives to S100B for CT triage in head injury patients, following the 2018 Food and Drug Administration clearance of the Abbott i-STAT platform in the United States.^47^

Finally, the updated recommendations emphasise structured observation as a potential alternative to immediate imaging in selected patients. While observation has long been used in clinical practice and has been shown to be equivalent to immediate head CT in the mTBI population,^48^ the latter has been shown to be more cost effective.^49^ The panel adopted 6 h from the time of injury as a consensus-based operational benchmark rather than an evidence-derived safety threshold. Discharge after this interval requires neurological status at premorbid baseline, stable or improving symptoms, no new clinical indication for CT, and reliable return precautions. The optimal duration and frequency of observation remain uncertain. This flexible approach aims to provide adaptability across healthcare systems with varying resource availability across Nordic settings.

The Nordic specificity of these guidelines arises from the intended health-system context, the high median age, access to CT and inter-hospital transfer, and the availability of selected biomarkers. The recommendations may be adaptable to other high-income settings with timely CT, trained clinical observation, reliable discharge supervision, and established pathways for urgent neurosurgical consultation or transfer. Their use should not be assumed where these capabilities differ. In low- and middle-income settings, limited CT availability, transport delays, staffing constraints, out-of-pocket costs, and limited access to biomarkers or neurosurgery can change the balance between imaging, observation, and transfer. Local adaptation, stakeholder engagement, resource assessment, and prospective validation are therefore required before implementation.

This guideline update has several strengths. The methodology followed established guideline development frameworks, including PRISMA, ACCORD, and AGREE, and incorporated both systematic evidence synthesis and multidisciplinary consensus. The inclusion of stakeholders from multiple specialities and Nordic countries enhances the clinical relevance of the guideline in its intended context. Furthermore, the development of flexible pathways accommodating biomarker availability increases applicability across various healthcare settings.

However, the limitations of our evidence review should be acknowledged. Outcome definitions varied across studies and included intracranial haemorrhage, broader CT abnormalities, and clinically defined ICI. This heterogeneity was considered in the certainty grading. A single reviewer conducted title screening, introducing a risk of selection bias. Publication bias cannot fully be assessed for variables where fewer than 10 studies were available by using funnel plots, and in these cases the funnel plots were retained only for exploratory purposes (Appendix). The overall certainty of evidence for many predictors remains moderate to low, reflecting the predominance of observational studies and heterogeneity in study populations. Heterogeneity was also present to a certain extent in outcome definitions, potentially impacting the interpretability of the pooled results. Additionally, consensus-based recommendations were necessary in areas with limited evidence, which may introduce subjectivity. Implementation may also vary across healthcare systems with differing resources and clinical workflows.

Another limitation is the use of emergency neurosurgical intervention as a major outcome in the meta-analysis. This endpoint is not a purely objective measure of injury severity, as it is influenced by institutional practice, resource availability, and patient-level factors such as age, frailty, comorbidities, and goals of care. Consequently, the absence of intervention does not equate to the absence of an ICI, particularly in older or conservatively managed patients. This limitation is especially important given the present guideline's focus on clinically meaningful outcomes. Neurosurgical intervention was therefore interpreted contextually as a pragmatic marker of severe and actionable injury rather than a definitive gold standard during the guideline development process.

The open and delegated stakeholder recruitment strategy was intended to maximise Nordic multidisciplinary participation but prevented calculation of an overall response rate and did not ensure balanced representation across specialities or countries. Although a neuroradiologist contributed as a guideline workgroup member, no radiologist participated as an external voting stakeholder in the modified Delphi process. This distinction is relevant because workgroup expertise does not fully replace independent stakeholder representation. Future updates should include more direct, targeted recruitment of radiologists and other underrepresented professional groups.

The modified Delphi process highlighted several implementation challenges. Stakeholders expressed concerns regarding potential overtriage of patients based on risk factors in the low-risk group, contrary to the intent of the guidelines to reduce unnecessary screening. However, these concerns were based primarily upon experience, with limited evidence to support them. This is also partially founded on the basis that Nordic mTBI populations have been shown in existing studies to have a median age of approximately 70 years.^14^, ^36^, ^50^, ^51^, ^52^ These considerations provide guidance for areas of exploration for future updates. Despite this, the high level of agreement achieved across stakeholders supports the clinical acceptability of the updated recommendations.

Further studies assessing biomarker-guided strategies, particularly concerning negative prediction (rule-out) in elderly populations and patients receiving antithrombotic therapy, are warranted. In addition, optimal strategies for the management of antithrombotic medications in CT-positive or asymptomatic patients not undergoing CT require further investigation in prospective trials. Like the advent of DOACs after 2013, the arrival of new antithrombotic medications such as novel activated factor XI inhibitors may also significantly alter patient outcomes.^53^

### Conclusion

The SNC 2026 guidelines provide an updated framework for the initial management of head injury with GCS score 9–15. The revised recommendations reflect evolving epidemiology and provide an updated stance on antithrombotic therapy and biomarkers, emphasising flexible risk-adapted clinical decision-making. While areas of limited evidence remain, broad multidisciplinary consensus supports the clinical applicability of the updated guidelines. Future validation studies will be essential to evaluate their impact and inform revisions.

## Contributors

LJY, JU, and EPT conceived and planned the guideline update process. LJY, JU and EPT all contributed to the data collection and data curation for the systematic review and meta-analysis. LJY conducted the statistical analysis. LJY, JU, AFS and EPT draughted the guidelines, which were critically reviewed and approved by all authors. All authors contributed to the coordination and moderation of the modified Delphi process. LJY, JU, and EPT accessed and verified the underlying data. All authors had access to the data presented, approved the final manuscript, and accepted responsibility for the decision to submit for publication.

## Data sharing statement

Data from the study is available upon reasonable request after approval of a research proposal.

## Declaration of interests

No funding was provided for the purpose of this study. LJY acknowledges personal funding support from the Swedish Society of Medicine and travel expense reimbursement from the Swedish Society of Emergency Medicine. AFS acknowledges personal funding support from Region Stockholm ALF. EPT acknowledges personal funding support from Region Stockholm Combined Academic and Clinical Position and ALF Funding, Karolinska Institutet, Swedish Research Council, Swedish Brain Foundation, and donations from Erik Gut and Family. JPP acknowledges personal funding from the Research Council of Finland, Sigrid Jusélius Foundation, and State Research Funding (VTR) of Finland. JPP acknowledges expert fees from the National Institute of Neurological Disorders and Stroke and lecture honoraria from Sanofi S.A., personal funding from the Finnish Medical Association and the Finnish Association of Otorhinolaryngology, and travel expense reimbursement from the National Institute of Neurological Disorders and Stroke. JU acknowledges personal funding support from the Scientific Council, Region Halland. None of the funders had a role in the design, conduct, analysis, interpretation, manuscript preparation, or decision to submit this work. The authors declare no additional competing interests. All authors are members of the Scandinavian Neurotrauma Committee, a non-profit organisation independent from external financial support.
