# Supplementary material for: The Scandinavian Neurotrauma Committee 2026 guidelines for initial management of minimal, mild and moderate head injuries in adults: a systematic review, meta-analysis and modified Delphi consensus study
Source: Lancet Reg Health Eur. 2026 Nov 7;72:101911. doi: 10.1016/j.lanepe.2026.101911 (PMC13769473; doi:10.1016/j.lanepe.2026.101911)
Supplement: Supplementary file 1 [file mmc1.pdf]

Appendix

1. Scandinavian Neurotrauma Committee Members 2
2. Complete Literature Search Strategy 3
3. Forest Plots (Traumatic Intracranial Injury) 4
4. Sensitivity Analysis (Traumatic Intracranial Injury) 13
5. Funnel Plots (Traumatic Intracranial Injury) 28
6. Forest Plots (Neurosurgical Intervention) 38
7. Sensitivity Analysis (Neurosurgical Intervention) 40
8. Funnel Plots (Neurosurgical Intervention) 43
9. Quality Assessment of Diagnostic Accuracy Studies (QUADAS) Summary (Traumatic Intracranial Injury) 47
10. Quality Assessment of Diagnostic Accuracy Studies (QUADAS) Summary (Neurosurgical Intervention) 50
11. Summary of Evidence Grading (Outcomes) 53
12. Summary of Evidence Grading (Individual Risk Factors) 54
13. Summary of the Modified Delphi Process (Quantitative Data) 57
14. List of Modified Delphi Process Participants 58
15. Variable Definitions and Harmonisation 59
16. Evidentiary Table 1 62
17. Evidentiary Table 2 66
18. Notes from the Modified Delphi Process 67
19. Guideline Flowcharts, Help Sheets and Discharge Information 68

1. Scandinavian Neurotrauma Committee Members

First Names	Surnames
Johan	Ljungqvist
Anders Christian	Feyling
David	Nelson
András	Buki
Mads	Aarhus
Tor	Brommeland
Ramona	Åstrand
Johan	Undén
Niklas	Marklund
Teemu	Luoto
Olga	Calcagnile
Jussi	Posti
Eric	Thelin
Elham	Rostami
Fredrik	Ginstman
Li Jin	Yang
Karoline	Skogen
Shirin	Kordasti
Fredrick	Wickbom
Alexander	Fletcher-Sandersjöö
Kirsten	Møller

2. Complete Literature Search Strategy

Question 1

MeSH terms:

((head trauma) OR (brain injury) OR (head injury) OR (traumatic head injury) OR (traumatic brain injury)) AND (minimal OR mild OR minor OR moderate) AND (management OR predictors OR predictor)

Searching Embase and MEDLINE: date filter and "English", "article"

('head trauma'/exp OR 'head trauma' OR (('head'/exp OR head) AND ('trauma'/exp OR trauma)) OR 'brain injury'/exp OR 'brain injury' OR (('brain'/exp OR brain) AND ('injury'/exp OR injury)) OR 'head injury'/exp OR 'head injury' OR (('head'/exp OR head) AND ('injury'/exp OR injury)) OR 'traumatic head injury'/exp OR 'traumatic head injury' OR (traumatic AND ('head'/exp OR head) AND ('injury'/exp OR injury)) OR 'traumatic brain injury'/exp OR 'traumatic brain injury' OR (traumatic AND ('brain'/exp OR brain) AND ('injury'/exp OR injury))) AND (minimal OR mild OR 'minor'/exp OR minor OR moderate) AND ('management'/exp OR management OR 'predictors'/exp OR predictors OR predictor) AND [article]/lim AND [english]/lim AND [01-08-2012]/sd NOT [02-04-2024]/sd

Results: 6929, Embase 1627, Embase AND MEDLINE 4393, MEDLINE 901

Question 2

MeSH Terms:

((head trauma) OR (brain injury) OR (head injury) OR (traumatic head injury) OR (traumatic brain injury)) AND (minimal OR mild OR minor OR moderate) AND (hospitalization OR hospitalisation OR observation OR admission OR discharge OR delayed OR ((normal OR negative OR repeat OR multiple OR serial OR follow-up) AND (CT OR CCT OR computed tomography)))

Searching Embase and MEDLINE: date filter and "English", "article"

('head trauma'/exp OR 'head trauma' OR (('head'/exp OR head) AND ('trauma'/exp OR trauma)) OR 'brain injury'/exp OR 'brain injury' OR (('brain'/exp OR brain) AND ('injury'/exp OR injury)) OR 'head injury'/exp OR 'head injury' OR (('head'/exp OR head) AND ('injury'/exp OR injury)) OR 'traumatic head injury'/exp OR 'traumatic head injury' OR (traumatic AND ('head'/exp OR head) AND ('injury'/exp OR injury)) OR 'traumatic brain injury'/exp OR 'traumatic brain injury' OR (traumatic AND ('brain'/exp OR brain) AND ('injury'/exp OR injury))) AND (minimal OR mild OR 'minor'/exp OR minor OR moderate) AND ('hospitalization'/exp OR hospitalization OR hospitalisation OR 'observation'/exp OR observation OR 'admission'/exp OR admission OR 'discharge'/exp OR discharge OR delayed OR ((normal OR negative OR repeat OR multiple OR 'serial'/exp OR serial OR 'follow up'/exp OR 'follow up') AND ('ct'/exp OR ct OR cct OR 'computed tomography'/exp OR 'computed tomography' OR (computed AND ('tomography'/exp OR tomography))))) AND [article]/lim AND [english]/lim AND [01-08-2012]/sd NOT [02-04-2024]/sd

Results: 6793, Embase 1794, Embase and MEDLINE 4383, MEDLINE 612

3. Forest Plots (Traumatic Intracranial Injury)

Age

Study	Age Under Threshold Events	Age Under Threshold Total	Age Over Threshold Events	Age Over Threshold Total	Weight	Odds Ratio MH, Random, 95% CI
Borczuk 1995	80	1165	39	283	4.7%	0.46 [0.31; 0.69]
Dunham 1996	35	220	128	2032	4.7%	2.81 [1.88; 4.21]
Arienta 1997	5	174	16	625	2.7%	1.13 [0.41; 3.12]
Haydel 2000	6	42	30	476	2.9%	2.48 [0.97; 6.34]
Stiell 2001	75	356	179	2690	5.1%	3.74 [2.78; 5.04]
Ibanez 2004	38	324	45	777	4.6%	2.16 [1.37; 3.40]
Munoz-Sanchez 2005	10	17	48	139	2.6%	2.71 [0.97; 7.57]
Stiell 2 2005	63	293	168	2414	5.0%	3.66 [2.66; 5.04]
Saboori 2006	5	40	41	642	2.7%	2.09 [0.78; 5.63]
Turedi 2008	0	30	8	90	0.6%	0.16 [0.01; 2.84]
Fabbri 2010	430	3377	450	14228	5.4%	4.47 [3.89; 5.13]
Kotlyar 2010	7	106	15	240	2.9%	1.06 [0.42; 2.68]
Kisat 2011	6661	13644	17105	67683	5.5%	2.82 [2.72; 2.93]
Zongo 2012	48	690	63	870	4.8%	0.96 [0.65; 1.41]
Barrow 2012	2	21	9	476	1.5%	5.46 [1.10; 27.04]
Nishijima 2013	50	800	10	182	3.7%	1.15 [0.57; 2.31]
Jame 2014	7	29	47	371	3.0%	2.19 [0.89; 5.42]
Mishra 2017	19	33	176	420	3.6%	1.88 [0.92; 3.85]
Zhang 2017	377	593	699	2549	5.3%	4.62 [3.83; 5.58]
Yuksen 2018	58	504	42	204	4.6%	0.50 [0.32; 0.78]
Galliazzo 2019	49	1042	19	804	4.3%	2.04 [1.19; 3.49]
Vaniyapong 2020	42	262	202	902	4.9%	0.66 [0.46; 0.95]
Yamada 2020	35	562	20	550	4.2%	1.76 [1.00; 3.09]
Leitner 2021	507	786	344	965	5.3%	3.28 [2.69; 3.99]
Singh 2023	3	13	11	87	1.8%	2.07 [0.49; 8.72]
Sakkas 2024	21	416	15	280	3.7%	0.94 [0.48; 1.86]
**Total (95% CI)**		**25539**		**100979**	**100.0%**	**1.85 [1.47; 2.33]**
**Prediction interval**						**[0.64; 5.36]**

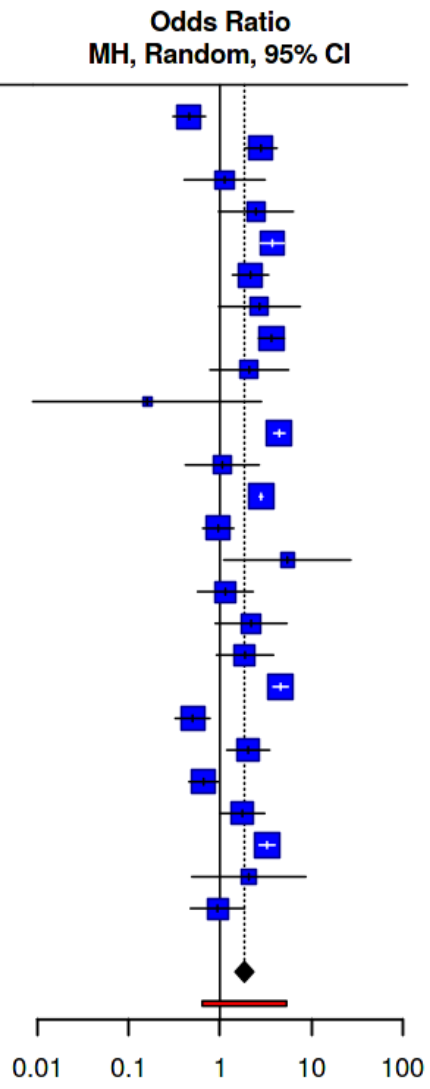

Heterogeneity: Tau^2 = 0.2524; Chi^2 = 339.94, df = 25 (P < 0.0001); I^2 = 92.6%
Test for overall effect: Z = 5.22 (P < 0.0001)

Anticoagulation

Study	Anticoagulation Events	Anticoagulation Total	No Anticoagulation Events	No Anticoagulation Total	Weight	Odds Ratio MH, Random, 95% CI
Borczuk 1995	8	51	111	1397	9.8%	2.16 [0.99; 4.70]
Mack 2003	3	18	16	115	7.1%	1.24 [0.32; 4.76]
Ibanez 2004	27	126	56	975	11.0%	4.48 [2.70; 7.41]
Kotlyar 2010	0	21	22	325	2.9%	0.31 [0.02; 5.35]
Claudia 2010	12	75	77	1335	10.3%	3.11 [1.61; 6.01]
Zongo 2012	78	390	33	1170	11.3%	8.61 [5.63; 13.19]
Asadollahi 2015	4	9	75	149	7.1%	0.79 [0.20; 3.06]
Vaniyapong 2020	4	24	240	1140	8.3%	0.75 [0.25; 2.21]
Yamada 2020	2	60	53	1052	6.7%	0.65 [0.15; 2.73]
Leitner 2021	384	573	467	1178	11.8%	3.09 [2.51; 3.81]
Martínez-Rivas 2023	20	271	77	636	10.9%	0.58 [0.35; 0.97]
Shafie 2023	0	3	100	495	2.7%	0.56 [0.03; 10.97]
**Total (95% CI)**		**1621**		**9967**	**100.0%**	**1.77 [1.01; 3.09]**
**Prediction interval**						**[0.26; 12.04]**

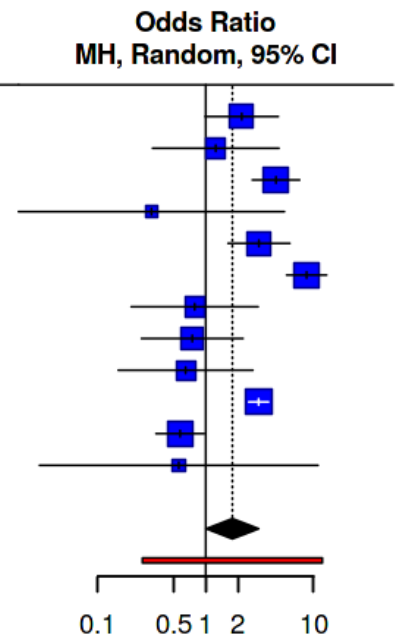

Heterogeneity: Tau^2 = 0.6789; Chi^2 = 86.18, df = 11 (P < 0.0001); I^2 = 87.2%
Test for overall effect: Z = 1.99 (P = 0.0468)

Antiplatelet Therapy

Study	APT Events	APT Total	No APT Events	No APT Total	Weight	Odds Ratio MH, Random, 95% CI
Mack 2003	2	29	17	104	1.8%	0.38 [0.08; 1.75]
Fabbri 2010	180	1366	700	12682	9.8%	2.60 [2.18; 3.09]
Kotlyar 2010	3	36	19	310	2.5%	1.39 [0.39; 4.96]
Nishijima 2013	30	279	30	703	6.7%	2.70 [1.60; 4.58]
Levine 2014	3	10	14	648	2.0%	19.41 [4.54; 82.95]
Cipriano 2018	0	10	23	196	0.6%	0.35 [0.02; 6.20]
Yuksen 2018	30	144	70	564	7.2%	1.86 [1.16; 2.98]
Galliazzo 2019	22	407	36	1222	6.5%	1.88 [1.09; 3.24]
Yamada 2020	13	125	42	997	5.6%	2.64 [1.37; 5.07]
Hofmann 2021	25	749	14	728	5.5%	1.76 [0.91; 3.42]
Leitner 2021	301	431	550	1321	9.4%	3.25 [2.57; 4.10]
Turcato 2022	23	148	267	2616	7.3%	1.62 [1.02; 2.57]
Colas 2023	29	495	17	297	5.9%	1.02 [0.55; 1.90]
Martínez-Rivas 2023	34	278	63	629	7.5%	1.25 [0.80; 1.95]
Schindler 2023	12	174	5	53	3.1%	0.71 [0.24; 2.12]
Shafie 2023	12	23	88	465	4.3%	4.67 [2.00; 10.94]
Sakkas 2024	20	380	16	316	5.4%	1.04 [0.53; 2.05]
Niklasson 2024	74	673	153	3136	8.9%	2.41 [1.80; 3.22]
**Total (95% CI)**		**5757**		**26987**	**100.0%**	**1.96 [1.56; 2.47]**
**Prediction interval**						**[0.88; 4.37]**

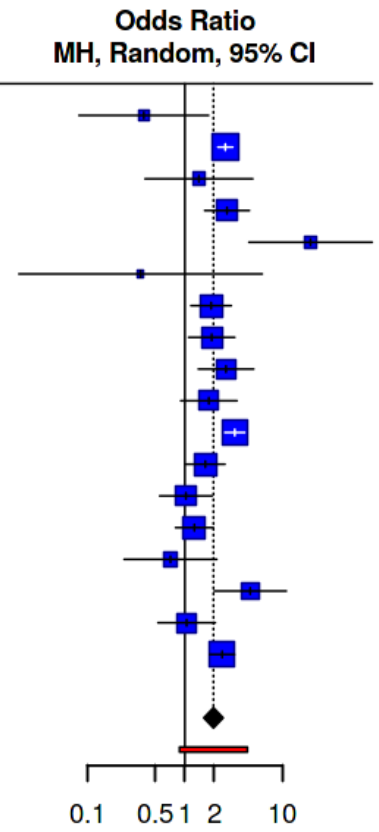

Heterogeneity: $Tau^2 = 0.1304$; $Chi^2 = 57.31$, df = 17 ($P < 0.0001$); $I^2 = 70.3\%$
Test for overall effect: $Z = 5.79$ ($P < 0.0001$)

Dual Antiplatelet Therapy (DAPT)

Study	DAPT Events	DAPT Total	No DAPT Events	No DAPT Total	Weight	Odds Ratio MH, Random, 95% CI
Galliazzo 2019	3	46	36	1222	19.2%	2.30 [0.68; 7.76]
Leitner 2021	20	31	831	1720	51.7%	1.95 [0.93; 4.08]
Niklasson 2024	2	7	153	3136	10.5%	7.80 [1.50; 40.52]
Sakkas 2024	3	34	33	662	18.6%	1.84 [0.54; 6.35]
**Total (95% CI)**		**118**		**6740**	**100.0%**	**2.30 [1.35; 3.92]**
**Prediction interval**						**[0.97; 5.47]**

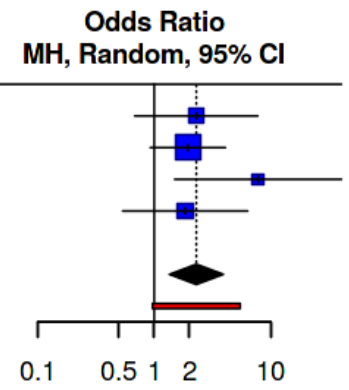

Heterogeneity: $Tau^2 = 0$; $Chi^2 = 2.49$, df = 3 ($P = 0.4779$); $I^2 = 0.0\%$
Test for overall effect: $Z = 3.06$ ($P = 0.0022$)

Direct Oral Anticoagulation (DOAC)

Study	DOAC Events	DOAC Total	No DOAC Events	No DOAC Total	Weight	Odds Ratio MH, Random, 95% CI
Cipriano 2018	4	85	19	121	6.6%	0.27 [0.09; 0.81]
Galliazzo 2019	2	49	36	1222	4.6%	1.40 [0.33; 6.00]
Savioli 2020	3	78	86	1599	6.2%	0.70 [0.22; 2.28]
Hofmann 2021	7	498	32	979	9.2%	0.42 [0.18; 0.96]
Leitner 2021	21	45	830	1706	11.9%	0.92 [0.51; 1.67]
Turcato 2022	90	1212	200	1842	16.0%	0.66 [0.51; 0.85]
Colas 2023	6	98	40	694	8.5%	1.07 [0.44; 2.58]
Martínez-Rivas 2023	4	55	93	852	7.2%	0.64 [0.23; 1.81]
Schindler 2023	3	34	14	193	5.4%	1.24 [0.34; 4.56]
Sakkas 2024	8	188	28	408	9.4%	0.60 [0.27; 1.35]
Niklasson 2024	53	676	153	3136	15.3%	1.66 [1.20; 2.29]
**Total (95% CI)**		**3018**		**12752**	**100.0%**	**0.79 [0.55; 1.14]**
**Prediction interval**						**[0.27; 2.31]**

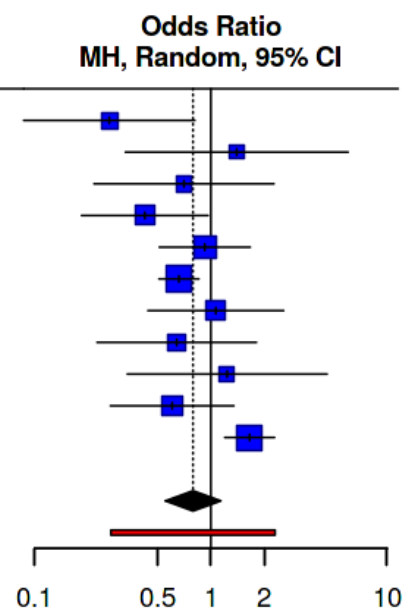

Heterogeneity: $Tau^2 = 0.1970$; $Chi^2 = 29.39$, df = 10 ($P = 0.0011$); $I^2 = 66.0\%$
Test for overall effect: $Z = -1.26$ ($P = 0.2070$)

Glasgow Coma Scale Score <15

Study	GCS<15 Events	GCS<15 Total	GCS=15 Events	GCS=15 Total	Weight	Odds Ratio MH, Random, 95% CI
Livingston 1991	6	11	9	79	2.3%	9.33 [2.36; 36.91]
Shackford 1992	186	712	282	1454	2.9%	1.47 [1.19; 1.82]
Harad 1992	12	51	43	251	2.7%	1.49 [0.72; 3.07]
Stein 1992	118	421	73	1117	2.9%	5.57 [4.05; 7.66]
Schynoll 1993	17	32	34	232	2.7%	6.60 [3.01; 14.45]
Borczuk 1995	47	237	72	1211	2.9%	3.91 [2.63; 5.83]
Culotta 1995	166	928	95	2179	2.9%	4.78 [3.66; 6.23]
Gomez 1996	58	97	37	90	2.8%	2.13 [1.19; 3.82]
Dunham 1996	83	551	45	1481	2.9%	5.66 [3.88; 8.26]
Hsiang 1997	75	183	218	1177	2.9%	3.05 [2.20; 4.25]
Stiell 2001	135	632	119	2489	2.9%	5.41 [4.15; 7.05]
Mussack 2002	9	10	10	129	1.7%	107.10 [12.30; 932.79]
Abdul Latip 2003	37	50	13	44	2.6%	6.79 [2.75; 16.77]
Mack 2003	3	20	16	113	2.3%	1.07 [0.28; 4.07]
Ibanez 2004	15	47	36	287	2.8%	3.27 [1.61; 6.62]
Stiell 2 2005	165	1119	66	1423	2.9%	3.56 [2.64; 4.79]
Biberthaler 2006	30	157	63	1152	2.9%	4.08 [2.55; 6.55]
Muller 2007	10	46	11	180	2.6%	4.27 [1.69; 10.80]
Turedi 2008	19	26	22	94	2.6%	8.88 [3.30; 23.89]
Fabbri 2010	565	818	315	12845	2.9%	88.83 [73.78; 106.96]
Jacobs 2010	436	472	1563	2312	2.9%	5.80 [4.09; 8.24]
Kotlyar 2010	7	35	15	303	2.6%	4.80 [1.81; 12.76]
Zongo 2012	37	374	74	1186	2.9%	1.65 [1.09; 2.49]
Barrow 2012	1	17	10	480	1.8%	2.94 [0.35; 24.35]
Nishijima 2013	15	103	45	879	2.8%	3.16 [1.69; 5.90]
Alrajhi 2015	14	56	14	92	2.7%	1.86 [0.81; 4.26]
Sugimoto 2017	19	26	35	47	2.5%	0.93 [0.31; 2.76]
Yuksen 2018	29	80	71	628	2.8%	4.46 [2.66; 7.49]
Galliazzo 2019	11	35	57	1811	2.7%	14.10 [6.59; 30.18]
Vaniyapong 2020	54	122	190	1042	2.9%	3.56 [2.41; 5.26]
Turcato 2022	88	365	202	2399	2.9%	3.46 [2.61; 4.57]
Colas 2023	4	62	45	778	2.5%	1.12 [0.39; 3.23]
Dupuis 2023	42	44	53	957	2.2%	358.19 [84.42; 1519.86]
Hosseininejad 2023	5	6	13	68	1.7%	21.15 [2.27; 196.82]
Shafie 2023	28	29	72	469	1.8%	154.39 [20.68; 1152.71]
Singh 2023	4	20	10	80	2.4%	1.75 [0.49; 6.30]
Chayoua 2024	32	101	27	152	2.8%	2.15 [1.19; 3.88]
Sakkas 2024	7	55	29	641	2.7%	3.08 [1.28; 7.39]
**Total (95% CI)**		**8150**		**42351**	**100.0%**	**4.89 [3.14; 7.61]**
**Prediction interval**						**[0.33; 72.74]**

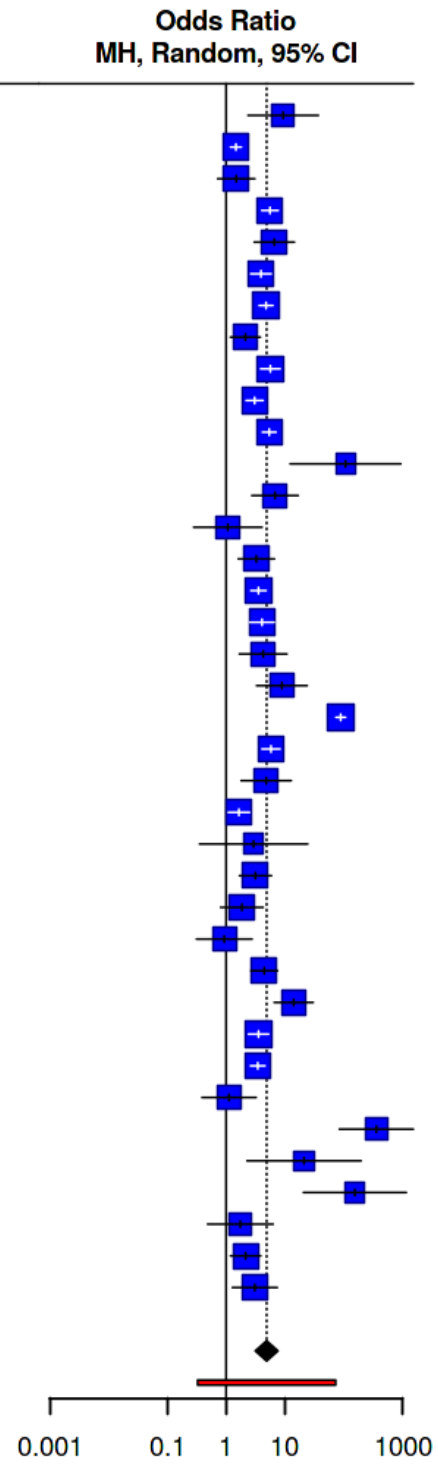

Heterogeneity: $Tau^2 = 1.7254$; $Chi^2 = 1226.64$, df = 37 ($P < 0.0001$); $I^2 = 97.0\%$
Test for overall effect: Z = 7.02 ($P < 0.0001$)

Headache

Study	Headache Events	Headache Total	No Headache Events	No Headache Total	Weight	Odds Ratio MH, Random, 95% CI
Jeret 1992	32	403	35	309	3.7%	0.68 [0.41; 1.12]
Schynoll 1993	9	22	151	206	3.1%	0.25 [0.10; 0.62]
Borczuk 1995	60	576	59	872	3.9%	1.60 [1.10; 2.33]
Miller 1996	75	692	63	1451	3.9%	2.68 [1.89; 3.79]
Holmes 1997	16	72	19	205	3.4%	2.80 [1.35; 5.80]
Haydel 2000	12	123	24	397	3.4%	1.68 [0.81; 3.47]
Stiell 2001	61	822	193	2299	4.0%	0.87 [0.65; 1.18]
Mack 2003	6	29	13	104	2.8%	1.83 [0.63; 5.32]
Falimirski 2003	9	52	32	279	3.2%	1.62 [0.72; 3.62]
Ibanez 2004	58	612	25	489	3.8%	1.94 [1.20; 3.16]
Stiell 2 2005	36	643	61	1179	3.9%	1.09 [0.71; 1.66]
Mower 2005	110	2334	807	11394	4.1%	0.65 [0.53; 0.80]
Saboori 2006	2	94	44	588	2.2%	0.27 [0.06; 1.13]
Turedi 2008	3	10	44	230	2.2%	1.81 [0.45; 7.29]
Fabbri 2010	156	1901	880	12327	4.1%	1.16 [0.97; 1.39]
Kotlyar 2010	18	148	4	198	2.7%	6.72 [2.22; 20.29]
Zongo 2012	24	273	87	1287	3.8%	1.33 [0.83; 2.13]
Barrow 2012	6	199	5	298	2.5%	1.82 [0.55; 6.05]
Nishijima 2013	30	349	30	633	3.7%	1.89 [1.12; 3.19]
Alrajhi 2015	14	56	14	92	3.2%	1.86 [0.81; 4.26]
Asadollahi 2015	48	84	31	74	3.5%	1.85 [0.98; 3.48]
Lagerstedt 2018	7	62	14	70	2.9%	0.51 [0.19; 1.36]
Yuksen 2018	63	189	37	519	3.8%	6.51 [4.15; 10.22]
Galliazzo 2019	1	31	67	1815	1.5%	0.87 [0.12; 6.47]
Vaniyapong 2020	171	377	73	787	4.0%	8.12 [5.93; 11.12]
Turcato 2022	27	112	263	2652	3.8%	2.89 [1.84; 4.53]
Hosseininejad 2023	8	42	10	32	2.8%	0.52 [0.18; 1.51]
Martínez-Rivas 2023	11	84	86	823	3.5%	1.29 [0.66; 2.53]
Shafie 2023	14	52	86	446	3.5%	1.54 [0.80; 2.97]
Sakkas 2024	10	42	26	654	3.2%	7.55 [3.35; 16.99]
**Total (95% CI)**		**10485**		**42709**	**100.0%**	**1.60 [1.18; 2.17]**
**Prediction interval**						**[0.32; 7.88]**

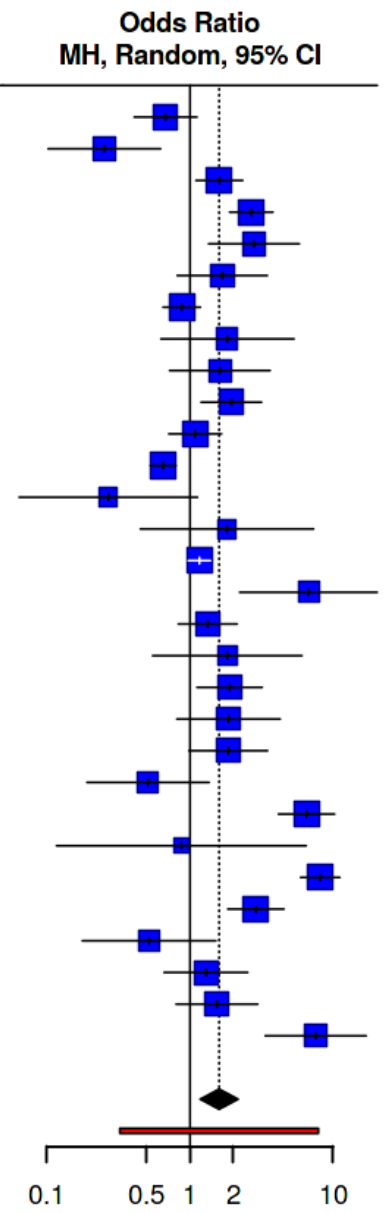

Heterogeneity: $Tau^2 = 0.5850$; $Chi^2 = 324.74$, df = 29 ($P < 0.0001$); $I^2 = 91.1\%$
Test for overall effect: Z = 3.00 (P = 0.0027)

Intoxication

Study	Intoxication Events	Intoxication Total	No Intoxication Events	No Intoxication Total	Weight	Odds Ratio MH, Random, 95% CI
Schynoll 1993	6	41	44	445	3.4%	1.56 [0.62; 3.92]
Borczuk 1995	30	448	89	1000	4.9%	0.73 [0.48; 1.13]
Dunham 1996	45	776	82	1256	5.0%	0.88 [0.61; 1.28]
Nagy 1999	6	247	33	923	3.5%	0.67 [0.28; 1.62]
Haydel 2000	22	180	14	340	4.1%	3.24 [1.62; 6.51]
Stiell 2001	46	383	208	2738	5.1%	1.66 [1.18; 2.33]
Mack 2003	3	9	16	124	2.1%	3.38 [0.77; 14.86]
Ibanez 2004	5	66	78	1035	3.4%	1.01 [0.39; 2.58]
Mower 2005	174	3295	743	10433	5.4%	0.73 [0.61; 0.86]
Stiell 2 2005	13	282	84	1527	4.4%	0.83 [0.46; 1.51]
Fabbri 2010	149	867	731	13361	5.4%	3.59 [2.96; 4.34]
Kotlyar 2010	7	84	15	262	3.4%	1.50 [0.59; 3.81]
Zongo 2012	28	374	83	1186	4.9%	1.08 [0.69; 1.68]
Barrow 2012	6	130	5	367	2.7%	3.50 [1.05; 11.68]
Nishijima 2013	4	34	56	948	3.0%	2.12 [0.72; 6.24]
Asadollahi 2015	11	18	68	140	3.2%	1.66 [0.61; 4.54]
Innocenti 2017	9	28	140	450	3.7%	1.05 [0.46; 2.38]
Zhang 2017	677	2021	399	1121	5.5%	0.91 [0.78; 1.06]
Yuksen 2018	6	32	94	676	3.4%	1.43 [0.57; 3.56]
Galliazzo 2019	3	76	65	1770	2.7%	1.08 [0.33; 3.51]
Vaniyapong 2020	73	399	171	765	5.2%	0.78 [0.57; 1.06]
Yamada 2020	13	255	42	867	4.3%	1.06 [0.56; 2.00]
Turcato 2022	6	65	284	2699	3.6%	0.86 [0.37; 2.02]
Colas 2023	6	66	43	774	3.5%	1.70 [0.70; 4.16]
Chayoua 2024	12	60	47	193	4.0%	0.78 [0.38; 1.58]
**Total (95% CI)**		**10236**		**45400**	**100.0%**	**1.24 [0.94; 1.64]**
**Prediction interval**						**[0.35; 4.39]**

Heterogeneity: $Tau^2 = 0.3544$; $Chi^2 = 214.17$, df = 24 ($P < 0.0001$); $I^2 = 88.8\%$
Test for overall effect: Z = 1.54 (P = 0.1226)

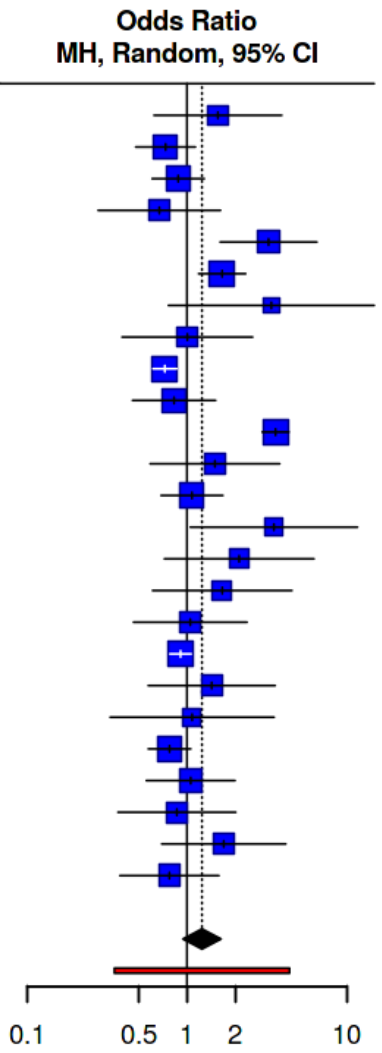

Loss of Consciousness (LOC)

Study	LOC Events	LOC Total	No LOC Events	No LOC Total	Weight	Odds Ratio MH, Random, 95% CI
Livingston 1991	12	91	3	20	1.6%	0.86 [0.22; 3.39]
Nelson 1992	18	105	2	26	1.4%	2.48 [0.54; 11.46]
Schynoll 1993	16	32	93	264	3.0%	1.84 [0.88; 3.84]
Moran 1994	8	93	0	103	0.5%	20.58 [1.17; 361.69]
Borczuk 1995	92	932	28	516	3.8%	1.91 [1.23; 2.96]
Hung 1996	1280	6821	723	9643	4.4%	2.85 [2.59; 3.14]
Stiell 2001	132	1435	122	1686	4.2%	1.30 [1.00; 1.68]
Mussack 2002	10	58	9	81	2.4%	1.67 [0.63; 4.40]
Mack 2003	8	39	11	94	2.3%	1.95 [0.72; 5.29]
Ibanez 2004	61	364	22	737	3.6%	6.54 [3.95; 10.85]
Mower 2005	578	6589	339	7139	4.3%	1.93 [1.68; 2.22]
Stiell 2 2005	123	1289	108	1295	4.1%	1.16 [0.88; 1.52]
Saboori 2007	4	78	42	604	2.2%	0.72 [0.25; 2.07]
Turedi 2008	7	17	40	223	2.3%	3.20 [1.15; 8.92]
Fabbri 2010	629	4230	251	9118	4.3%	6.17 [5.30; 7.18]
Brewer 2010	15	35	17	93	2.7%	3.35 [1.43; 7.85]
Kotlyar 2010	12	125	10	221	2.6%	2.24 [0.94; 5.35]
Zongo 2012	62	627	49	933	3.9%	1.98 [1.34; 2.92]
Barrow 2012	8	113	3	384	1.7%	9.68 [2.52; 37.12]
Alrajhi 2015	8	16	20	132	2.1%	5.60 [1.88; 16.65]
Asadollahi 2015	53	90	26	68	3.2%	2.31 [1.21; 4.41]
Mishra 2017	90	194	105	259	3.9%	1.27 [0.87; 1.85]
Cipriano 2018	1	12	22	194	0.9%	0.71 [0.09; 5.77]
Lagerstedt 2018	20	117	1	15	0.9%	2.89 [0.36; 23.22]
Yuksen 2018	54	150	46	558	3.7%	6.26 [3.99; 9.82]
Foks 2019	147	1184	99	1925	4.2%	2.61 [2.00; 3.41]
Galliazzo 2019	5	68	63	1778	2.5%	2.16 [0.84; 5.56]
Vaniyapong 2020	161	742	83	422	4.1%	1.13 [0.84; 1.52]
Teeratakulpisarn 2021	14	44	10	56	2.5%	2.15 [0.84; 5.46]
Turcato 2022	60	172	230	2592	4.0%	5.50 [3.91; 7.74]
Colas 2023	8	114	41	726	2.8%	1.26 [0.58; 2.76]
Shafie 2023	24	79	76	419	3.5%	1.97 [1.15; 3.38]
Chayoua 2024	42	132	17	121	3.3%	2.85 [1.52; 5.36]
Sakkas 2024	12	89	24	607	3.0%	3.79 [1.82; 7.88]
**Total (95% CI)**		**26276**		**43052**	**100.0%**	**2.40 [1.92; 3.00]**
**Prediction interval**						**[0.78; 7.36]**

Heterogeneity: $Tau^2 = 0.2903$; $Chi^2 = 328.25$, df = 33 ($P < 0.0001$); $I^2 = 89.9\%$
Test for overall effect: Z = 7.73 ($P < 0.0001$)

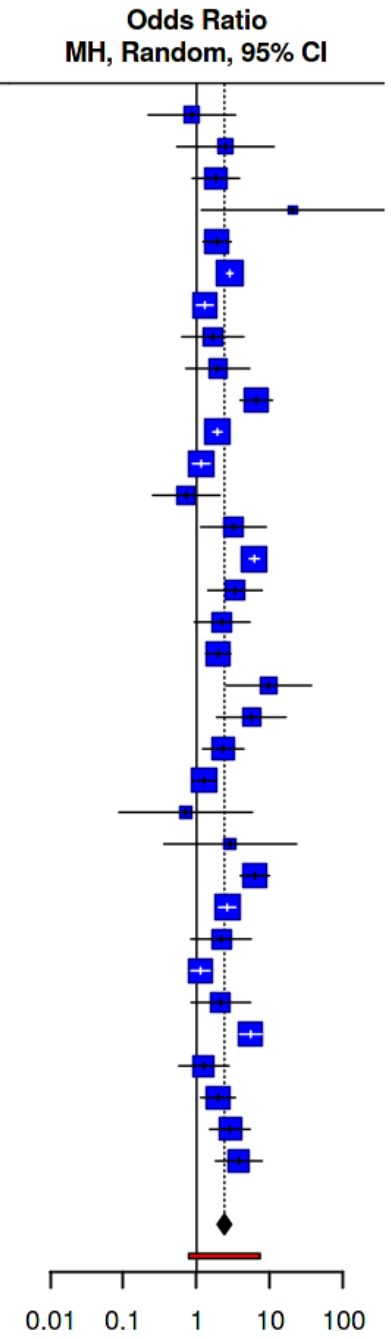

Male Sex

Study	Male Sex Events	Male Sex Total	Female Sex Events	Female Sex Total	Weight	Odds Ratio MH, Random, 95% CI
Alrajhi 2015	14	56	14	92	3.0%	1.86 [0.81; 4.26]
Asadollahi 2015	52	110	27	48	3.9%	0.70 [0.35; 1.38]
Innocenti 2017	97	333	52	145	6.0%	0.74 [0.49; 1.11]
Mishra 2017	160	367	35	86	5.4%	1.13 [0.70; 1.81]
Sugimoto 2017	28	44	26	29	1.5%	0.20 [0.05; 0.77]
Zhang 2017	679	2019	397	1123	8.3%	0.93 [0.79; 1.08]
Cipriano 2018	12	84	11	122	2.8%	1.68 [0.70; 4.02]
Gill 2018	45	163	24	114	4.7%	1.43 [0.81; 2.52]
Yuksen 2018	64	291	36	417	5.7%	2.98 [1.92; 4.63]
Galliazzo 2019	36	926	32	920	5.3%	1.12 [0.69; 1.82]
Vaniyapong 2020	171	764	73	400	7.0%	1.29 [0.95; 1.75]
Yamada 2020	30	591	25	531	4.8%	1.08 [0.63; 1.87]
Leitner 2021	450	990	401	761	8.0%	0.75 [0.62; 0.90]
Teeratakulpisarn 2021	13	49	11	27	2.4%	0.53 [0.19; 1.42]
Turcato 2022	146	1414	144	1640	7.6%	1.20 [0.94; 1.52]
Colas 2023	14	315	35	525	4.2%	0.65 [0.34; 1.23]
Dupuis 2023	44	413	51	588	5.9%	1.26 [0.82; 1.92]
Hosseininejad 2023	5	43	3	31	1.2%	1.23 [0.27; 5.57]
Shafie 2023	89	393	11	105	3.9%	2.50 [1.28; 4.88]
Chayoua 2024	37	146	22	107	4.4%	1.31 [0.72; 2.39]
Sakkas 2024	17	316	19	380	3.9%	1.08 [0.55; 2.12]
**Total (95% CI)**		**9827**		**8191**	**100.0%**	**1.12 [0.93; 1.33]**
**Prediction interval**						**[0.57; 2.17]**

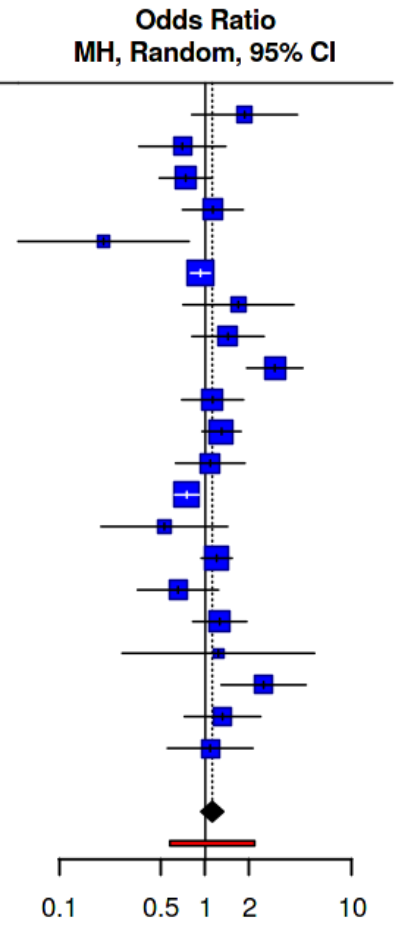

Heterogeneity: $Tau^2 = 0.0939$; $Chi^2 = 65.02$, df = 20 ($P < 0.0001$); $I^2 = 69.2\%$
Test for overall effect: Z = 1.20 (P = 0.2282)

Neurological Deficit

Study	Neurological Deficit Events	Neurological Deficit Total	No Deficit Events	No Deficit Total	Weight	Odds Ratio MH, Random, 95% CI
Schynoll 1993	6	9	26	255	5.6%	17.62 [4.16; 74.65]
Borczuk 1995	4	192	115	1256	6.0%	0.21 [0.08; 0.58]
Livingston 2000	135	698	201	1454	6.4%	1.49 [1.18; 1.90]
Vilke 2000	1	1	2	57	3.5%	66.60 [2.13; 2081.91]
Abdul Latip 2003	2	2	48	92	3.9%	4.59 [0.21; 98.19]
Falimirski 2003	0	7	40	324	4.1%	0.47 [0.03; 8.36]
Ibanez 2004	4	11	79	1090	5.8%	7.31 [2.10; 25.51]
Mower 2005	596	3844	321	9884	6.4%	5.47 [4.75; 6.30]
Saboori 2007	1	1	45	681	3.7%	41.97 [1.69; 1044.81]
Fabbri 2010	485	534	395	13209	6.3%	321.09 [235.41; 437.96]
Barrow 2012	2	18	9	479	5.4%	6.53 [1.30; 32.70]
Yuksen 2018	31	74	69	634	6.3%	5.90 [3.49; 9.98]
Galliazzo 2019	1	15	67	1831	5.0%	1.88 [0.24; 14.51]
Vaniyapong 2020	7	9	237	1155	5.5%	13.56 [2.80; 65.68]
Yamada 2020	6	40	49	1082	6.0%	3.72 [1.49; 9.28]
Turcato 2022	15	38	275	2726	6.2%	5.81 [3.00; 11.27]
Colas 2023	0	20	49	820	4.1%	0.38 [0.02; 6.38]
Dupuis 2023	30	30	65	971	4.1%	844.22 [51.05; 13961.78]
Sakkas 2024	3	45	33	651	5.8%	1.34 [0.39; 4.54]
**Total (95% CI)**		**5588**		**38651**	**100.0%**	**6.22 [2.38; 16.27]**
**Prediction interval**						**[0.09; 416.42]**

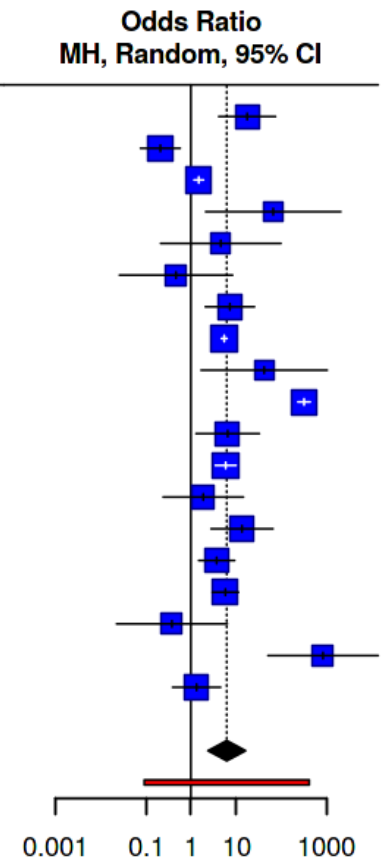

Heterogeneity: $Tau^2 = 3.7622$; $Chi^2 = 834.87$, df = 18 ($P < 0.0001$); $I^2 = 97.8\%$
Test for overall effect: Z = 3.73 (P = 0.0002)

Post-traumatic Amnesia (PTA)

Study	PTA Events	PTA Total	No PTA Events	No PTA Total	Weight	Odds Ratio MH, Random, 95% CI
Schynoll 1993	11	22	80	240	3.0%	2.00 [0.83; 4.81]
Borczuk 1995	37	280	82	1168	4.6%	2.02 [1.34; 3.05]
Haydel 2000	5	9	31	511	1.9%	19.35 [4.95; 75.71]
Stiell 2001	236	2722	18	399	4.3%	2.01 [1.23; 3.28]
Mussack 2002	12	83	7	56	2.7%	1.18 [0.43; 3.22]
Mack 2003	3	26	16	107	2.0%	0.74 [0.20; 2.76]
Ibanez 2004	18	109	65	992	4.0%	2.82 [1.60; 4.96]
Stiell 2 2005	53	552	44	1270	4.6%	2.96 [1.96; 4.47]
Saboori 2007	4	44	42	638	2.5%	1.42 [0.48; 4.16]
Turedi 2008	9	29	32	91	3.0%	0.83 [0.34; 2.03]
Fabbri 2010	457	5801	423	7547	5.2%	1.44 [1.26; 1.65]
Kotlyar 2010	8	75	14	271	2.9%	2.19 [0.88; 5.44]
Zongo 2012	61	552	50	1008	4.6%	2.38 [1.61; 3.51]
Barrow 2012	0	21	11	476	0.6%	0.94 [0.05; 16.51]
Jame 2014	0	7	54	393	0.6%	0.42 [0.02; 7.38]
Alrajhi 2015	4	16	24	132	2.2%	1.50 [0.45; 5.06]
Asadollahi 2015	46	76	33	82	3.8%	2.28 [1.20; 4.31]
Cipriano 2018	5	20	18	186	2.4%	3.11 [1.01; 9.56]
Lagerstedt 2018	15	83	6	49	2.6%	1.58 [0.57; 4.39]
Yuksen 2018	48	154	52	554	4.5%	4.37 [2.80; 6.82]
Foks 2019	135	904	111	2205	5.0%	3.31 [2.54; 4.31]
Galliazzo 2019	25	140	43	1706	4.2%	8.41 [4.96; 14.25]
Vaniyapong 2020	153	661	91	503	4.9%	1.36 [1.02; 1.82]
Turcato 2022	89	297	201	2467	4.9%	4.82 [3.62; 6.43]
Colas 2023	16	193	33	647	3.9%	1.68 [0.90; 3.13]
Martínez-Rivas 2023	18	102	79	805	4.1%	1.97 [1.13; 3.45]
Shafie 2023	38	79	62	419	4.2%	5.34 [3.18; 8.95]
Chayoua 2024	49	187	10	66	3.4%	1.99 [0.94; 4.20]
Sakkas 2024	14	140	22	556	3.6%	2.70 [1.34; 5.42]
**Total (95% CI)**		**13384**		**25544**	**100.0%**	**2.39 [1.89; 3.02]**
**Prediction interval**						**[0.81; 7.06]**

Heterogeneity: Tau^2 = 0.2655; Chi^2 = 158.07, df = 28 (P < 0.0001); I^2 = 82.3%
Test for overall effect: Z = 7.34 (P < 0.0001)

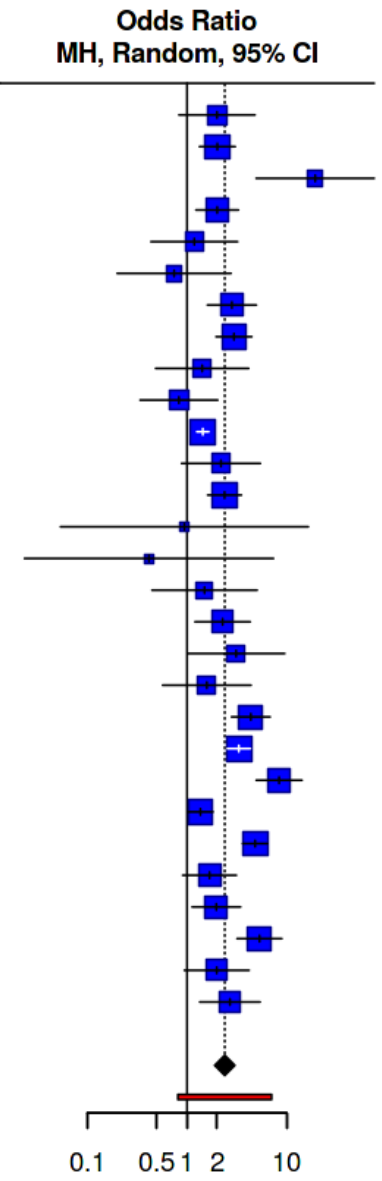

Repeated Vomiting

Study	Repeated Vomiting Events	Repeated Vomiting Total	No Repeated Vomiting Events	No Repeated Vomiting Total	Weight	Odds Ratio MH, Random, 95% CI
Stiell 2001	77	301	177	2743	17.5%	4.98 [3.69; 6.73]
Stiell 2 2005	63	393	268	2414	17.5%	1.53 [1.13; 2.06]
Mower 2005	92	824	825	12904	18.2%	1.84 [1.47; 2.31]
Barrow 2012	3	64	8	433	6.2%	2.61 [0.67; 10.12]
Jame 2014	13	40	41	360	11.9%	3.75 [1.79; 7.83]
Asadollahi 2015	34	57	45	101	12.9%	1.84 [0.95; 3.55]
Vaniyapong 2020	37	93	207	1071	15.8%	2.76 [1.77; 4.29]
**Total (95% CI)**		**1772**		**20026**	**100.0%**	**2.51 [1.67; 3.78]**
**Prediction interval**						**[0.71; 8.92]**

Heterogeneity: Tau^2 = 0.2244; Chi^2 = 39.32, df = 6 (P < 0.0001); I^2 = 84.7%
Test for overall effect: Z = 4.42 (P < 0.0001)

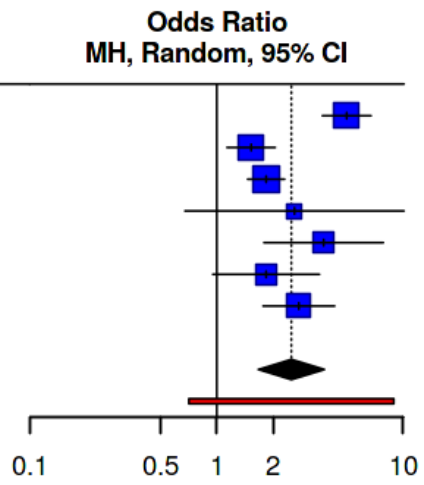

Scalp Wound

Study	Scalp Wound Events	Scalp Wound Total	No Scalp Wound Events	No Scalp Wound Total	Weight	Odds Ratio MH, Random, 95% CI
Mower 2005	431	4530	486	9198	28.1%	1.88 [1.65; 2.16]
Kotlyar 2010	13	136	9	210	13.4%	2.36 [0.98; 5.69]
Galliazzo 2019	59	1207	9	639	16.5%	3.60 [1.77; 7.30]
Vaniyapong 2020	190	620	54	544	24.9%	4.01 [2.89; 5.57]
Yamada 2020	44	816	11	306	17.2%	1.53 [0.78; 3.00]
**Total (95% CI)**		**7309**		**10897**	**100.0%**	**2.51 [1.62; 3.90]**
**Prediction interval**						**[0.68; 9.32]**

Heterogeneity: Tau^2 = 0.1728; Chi^2 = 20.58, df = 4 (P = 0.0004); I^2 = 80.6%
Test for overall effect: Z = 4.13 (P < 0.0001)

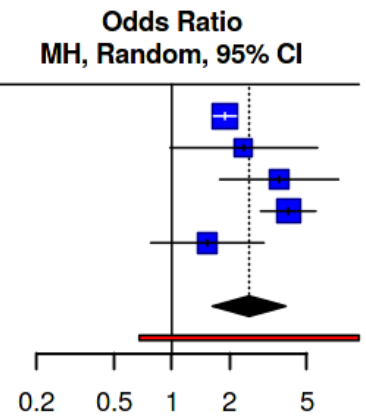

Post-traumatic Seizure

Study	Seizure Events	Seizure Total	No Seizure Events	No Seizure Total	Weight	Odds Ratio MH, Random, 95% CI
Schynoll 1993	1	32	9	222	3.3%	0.76 [0.09; 6.23]
Haydel 2000	4	24	32	496	6.1%	2.90 [0.94; 8.99]
Stiell 2001	1	4	253	3116	3.0%	3.77 [0.39; 36.40]
Falimirski 2003	1	3	39	328	2.7%	3.71 [0.33; 41.82]
Ibanez 2004	1	7	82	1094	3.3%	2.06 [0.24; 17.29]
Mower 2005	49	550	868	13178	9.0%	1.39 [1.03; 1.87]
Stiell 2 2005	4	36	93	1782	6.4%	2.27 [0.79; 6.55]
Saboori 2007	0	2	46	680	1.9%	2.73 [0.13; 57.68]
Turedi 2008	1	1	46	239	1.8%	12.48 [0.50; 311.38]
Fabbri 2010	77	262	803	13966	9.1%	6.82 [5.18; 8.98]
Kotlyar 2010	1	3	21	322	2.7%	7.17 [0.62; 82.30]
Zongo 2012	2	26	109	1534	5.0%	1.09 [0.25; 4.67]
Barrow 2012	0	3	11	494	2.0%	6.01 [0.29; 123.12]
Jame 2014	1	9	53	391	3.3%	0.80 [0.10; 6.50]
Asadollahi 2015	11	20	68	138	6.9%	1.26 [0.49; 3.23]
Mishra 2017	10	20	185	433	7.0%	1.34 [0.55; 3.29]
Yuksen 2018	3	20	97	688	5.7%	1.08 [0.31; 3.74]
Galliazzo 2019	0	4	68	1842	2.1%	2.88 [0.15; 54.00]
Vaniyapong 2020	15	50	229	1114	8.1%	1.66 [0.89; 3.09]
Turcato 2022	3	9	287	2755	5.2%	4.30 [1.07; 17.28]
Martínez-Rivas 2023	1	2	96	905	2.2%	8.43 [0.52; 135.82]
Sakkas 2024	1	5	35	691	3.1%	4.69 [0.51; 43.04]
**Total (95% CI)**		**1092**		**46408**	**100.0%**	**2.31 [1.43; 3.72]**
**Prediction interval**						**[0.41; 13.11]**

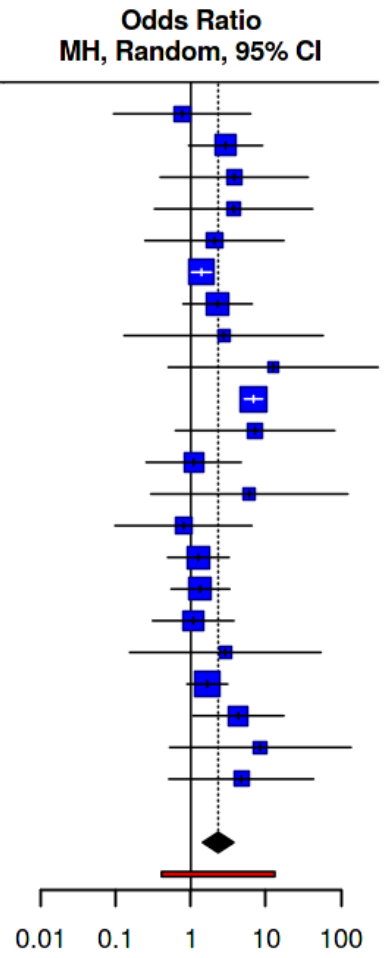

Heterogeneity: $Tau^2 = 0.6379$; $Chi^2 = 83.51$, df = 21 ($P < 0.0001$); $I^2 = 74.9\%$
Test for overall effect: Z = 3.43 (P = 0.0006)

Signs of Injury Above Clavicle

Study	Signs of Injury Above Clavicle Events	Signs of Injury Above Clavicle Total	No Signs Events	No Signs Total	Weight	Odds Ratio MH, Random, 95% CI
Holmes 1997	33	185	2	76	5.9%	8.03 [1.88; 34.38]
Haydel 2000	32	338	4	182	8.3%	4.65 [1.62; 13.37]
Stiell 2005	55	928	42	839	13.6%	1.20 [0.79; 1.81]
Fabbri 2010	629	6819	251	6780	15.1%	2.64 [2.27; 3.07]
Brewer 2010	28	108	13	33	10.2%	0.54 [0.24; 1.22]
Kotlyar 2010	13	126	9	220	9.7%	2.70 [1.12; 6.50]
Barrow 2012	1	224	10	273	3.6%	0.12 [0.01; 0.93]
Nishijima 2013	41	696	19	286	12.4%	0.88 [0.50; 1.54]
Jame 2014	8	12	46	388	7.1%	14.87 [4.31; 51.34]
Turcato 2022	243	2023	47	741	14.2%	2.02 [1.46; 2.79]
**Total (95% CI)**		**11459**		**9818**	**100.0%**	**1.92 [1.22; 3.01]**
**Prediction interval**						**[0.46; 8.00]**

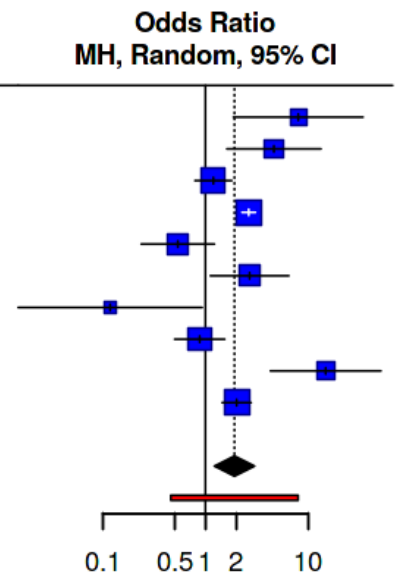

Heterogeneity: $Tau^2 = 0.3454$; $Chi^2 = 57.91$, df = 9 ($P < 0.0001$); $I^2 = 84.5\%$
Test for overall effect: Z = 2.84 (P = 0.0046)

Signs of Skull Fracture

Study	Signs of Skull Fracture Events	Signs of Skull Fracture Total	No Signs Events	No Signs Total	Weight	Odds Ratio MH, Random, 95% CI
Schynoll 1993	7	21	25	243	5.5%	4.36 [1.61; 11.82]
Borczuk 1995	28	48	91	1400	6.6%	20.14 [10.92; 37.13]
Stiell 2001	77	210	177	2911	7.2%	8.94 [6.50; 12.31]
Ibanez 2004	16	37	67	1064	6.4%	11.34 [5.65; 22.74]
Mower 2005	193	549	724	12179	7.4%	8.58 [7.09; 10.38]
Stiell 2 2005	69	158	162	2549	7.1%	11.42 [8.03; 16.25]
Turedi 2008	19	40	28	200	6.3%	5.56 [2.66; 11.63]
Fabbri 2010	286	372	594	13570	7.3%	72.65 [56.31; 93.72]
Kotlyar 2010	3	6	19	340	3.8%	16.89 [3.19; 89.38]
Barrow 2012	0	1	11	496	1.6%	14.07 [0.54; 364.24]
Jame 2014	40	88	14	312	6.4%	17.74 [8.98; 35.04]
Zhang 2017	546	805	530	2337	7.4%	7.19 [6.02; 8.58]
Yuksen 2018	17	20	83	688	4.8%	41.31 [11.85; 143.97]
Galliazzo 2019	5	12	63	1834	5.0%	20.08 [6.20; 65.01]
Vaniyapong 2020	50	81	194	1083	6.9%	7.39 [4.60; 11.88]
Colas 2023	1	21	48	819	3.1%	0.80 [0.11; 6.11]
Shafie 2023	36	41	64	457	5.6%	44.21 [16.73; 116.86]
Sakkas 2024	1	1	35	695	1.6%	55.82 [2.23; 1394.72]
**Total (95% CI)**		**2511**		**43177**	**100.0%**	**12.98 [8.16; 20.63]**
**Prediction interval**						**[1.95; 86.44]**

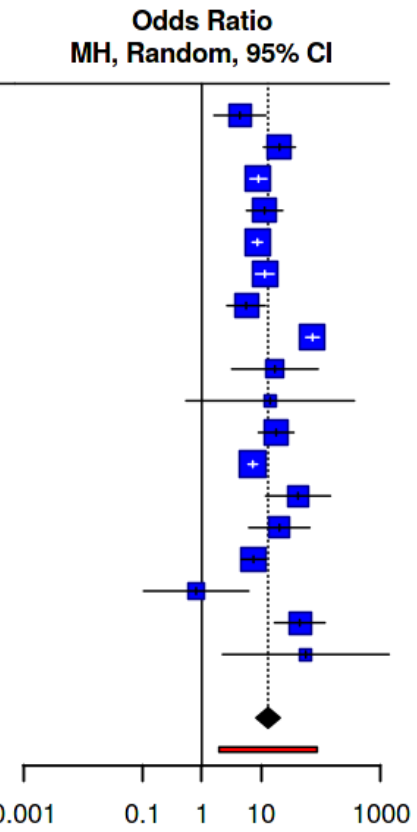

Heterogeneity: $Tau^2 = 0.7519$; $Chi^2 = 277.09$, df = 17 ($P < 0.0001$); $I^2 = 93.9\%$
Test for overall effect: Z = 10.83 ($P < 0.0001$)

Vitamin K Antagonist

Study	VKA Events	VKA Total	No VKA Events	No VKA Total	Weight	Odds Ratio MH, Random, 95% CI
Borczuk 1995	8	51	111	1397	6.0%	2.16 [0.99; 4.70]
Mack 2003	3	18	16	115	4.4%	1.24 [0.32; 4.76]
Ibanez 2004	27	126	56	975	6.8%	4.48 [2.70; 7.41]
Kotlyar 2010	0	21	22	325	1.8%	0.31 [0.02; 5.35]
Claudia 2010	12	75	77	1335	6.4%	3.11 [1.61; 6.01]
Zongo 2012	78	390	33	1170	6.9%	8.61 [5.63; 13.19]
Nishijima 2013	31	714	29	268	6.7%	0.37 [0.22; 0.63]
Yuksen 2018	2	23	98	685	4.1%	0.57 [0.13; 2.47]
Galliazzo 2019	5	120	36	1222	5.5%	1.43 [0.55; 3.72]
Savioli 2020	22	156	67	1521	6.7%	3.56 [2.13; 5.95]
Hofmann 2021	7	185	32	1292	5.9%	1.55 [0.67; 3.56]
Leitner 2021	82	126	769	1625	7.0%	2.07 [1.42; 3.03]
Turcato 2022	200	1842	90	1212	7.2%	1.52 [1.17; 1.97]
Colas 2023	11	199	35	593	6.3%	0.93 [0.46; 1.87]
Martínez-Rivas 2023	16	216	81	691	6.6%	0.60 [0.34; 1.05]
Sakkas 2024	4	88	32	608	5.2%	0.86 [0.30; 2.48]
Niklasson 2024	13	299	153	3136	6.6%	0.89 [0.50; 1.58]
**Total (95% CI)**		**4649**		**18170**	**100.0%**	**1.53 [0.99; 2.38]**
**Prediction interval**						**[0.25; 9.29]**

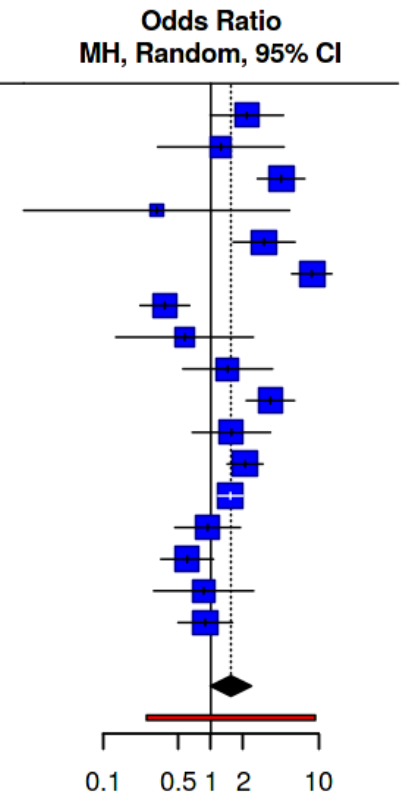

Heterogeneity: $Tau^2 = 0.6722$; $Chi^2 = 141.35$, df = 16 ($P < 0.0001$); $I^2 = 88.7\%$
Test for overall effect: Z = 1.91 ($P = 0.0556$)

Vomiting Once

Study	Vomiting Events	Vomiting Total	No Vomiting Events	No Vomiting Total	Weight	Odds Ratio MH, Random, 95% CI
Jeret 1992	8	51	59	661	4.8%	1.90 [0.85; 4.23]
Ando 1992	5	26	6	121	3.8%	4.56 [1.28; 16.33]
Schynoll 1993	2	32	77	261	3.5%	0.16 [0.04; 0.68]
Holmes 1997	5	10	35	261	3.8%	6.46 [1.78; 23.45]
Hsiang 1997	42	186	120	550	5.5%	1.05 [0.70; 1.56]
Miller 1997	21	122	117	2021	5.3%	3.38 [2.04; 5.61]
Haydel 2000	4	47	32	473	4.2%	1.28 [0.43; 3.80]
Mussack 2002	4	14	15	125	3.8%	2.93 [0.82; 10.54]
Abdul Latip 2003	27	40	23	54	4.7%	2.80 [1.19; 6.57]
Falimirski 2003	4	19	37	271	4.1%	1.69 [0.53; 5.36]
Ibanez 2004	19	88	64	1013	5.2%	4.08 [2.32; 7.20]
Saboori 2006	8	39	38	643	4.7%	4.11 [1.77; 9.55]
Turedi 2008	28	63	19	177	5.0%	6.65 [3.34; 13.24]
Fabbri 2010	173	377	707	13851	5.7%	15.77 [12.70; 19.57]
Nishijima 2013	8	41	52	941	4.8%	4.14 [1.82; 9.42]
Mishra 2017	88	166	107	287	5.5%	1.90 [1.29; 2.80]
Zhang 2017	537	1105	539	2037	5.7%	2.63 [2.25; 3.06]
Lagerstedt 2018	6	33	15	99	4.3%	1.24 [0.44; 3.53]
Yuksen 2018	13	34	87	674	4.9%	4.18 [2.02; 8.64]
Turcato 2022	31	84	259	2680	5.4%	5.47 [3.45; 8.67]
Shafie 2023	42	90	58	408	5.3%	5.28 [3.21; 8.69]
**Total (95% CI)**		**2667**		**27608**	**100.0%**	**2.98 [1.94; 4.60]**
**Prediction interval**						**[0.41; 21.48]**

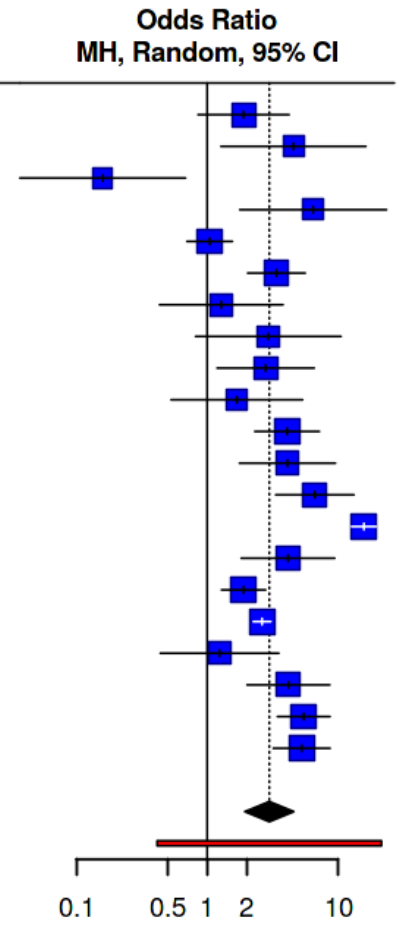

Heterogeneity: $Tau^2 = 0.8464$; $Chi^2 = 294.66$, df = 20 ($P < 0.0001$); $I^2 = 93.2\%$
Test for overall effect: $Z = 4.96$ ($P < 0.0001$)

4. Sensitivity Analysis (Traumatic Intracranial Injury)

Age

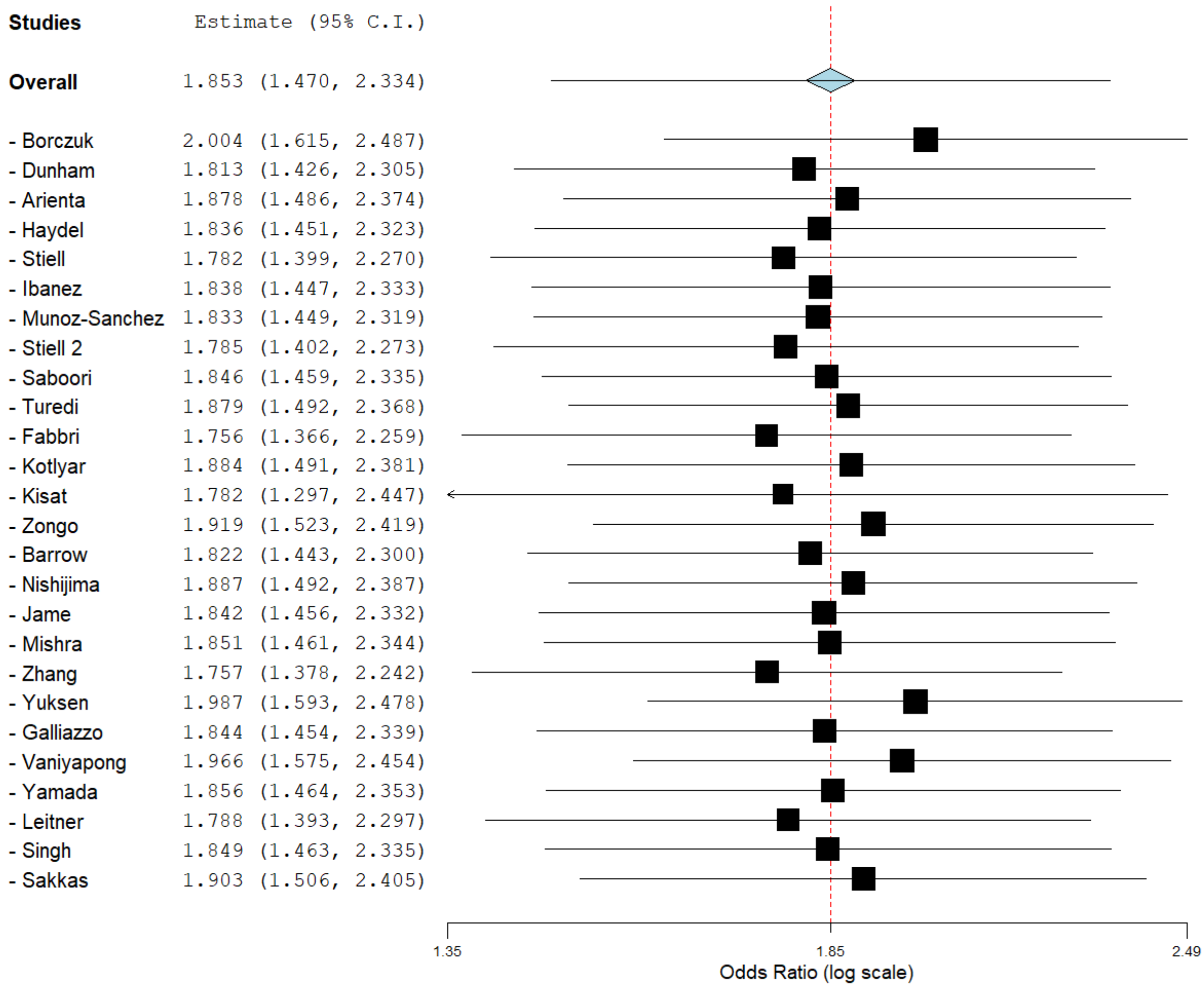

Anticoagulation

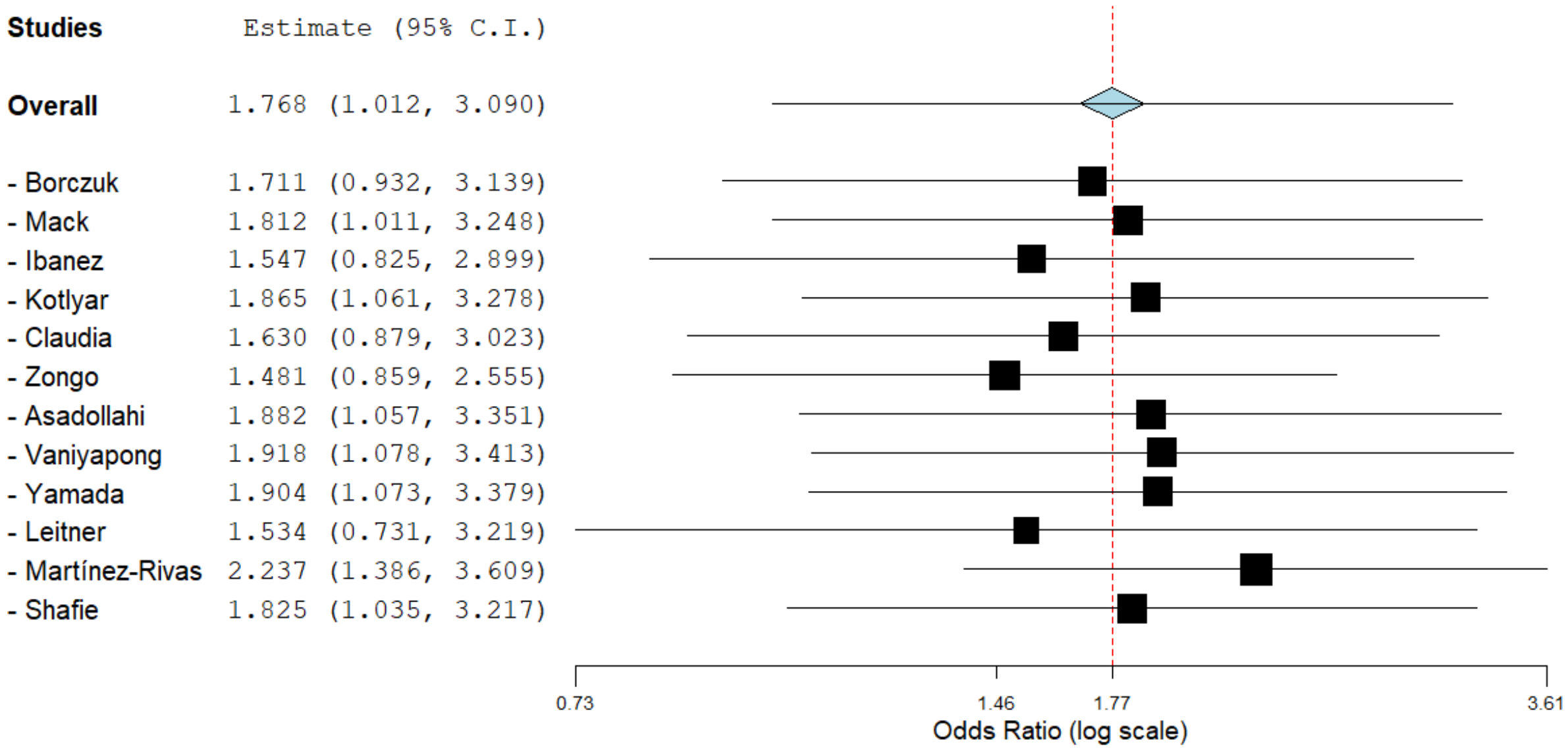

Antiplatelet Therapy

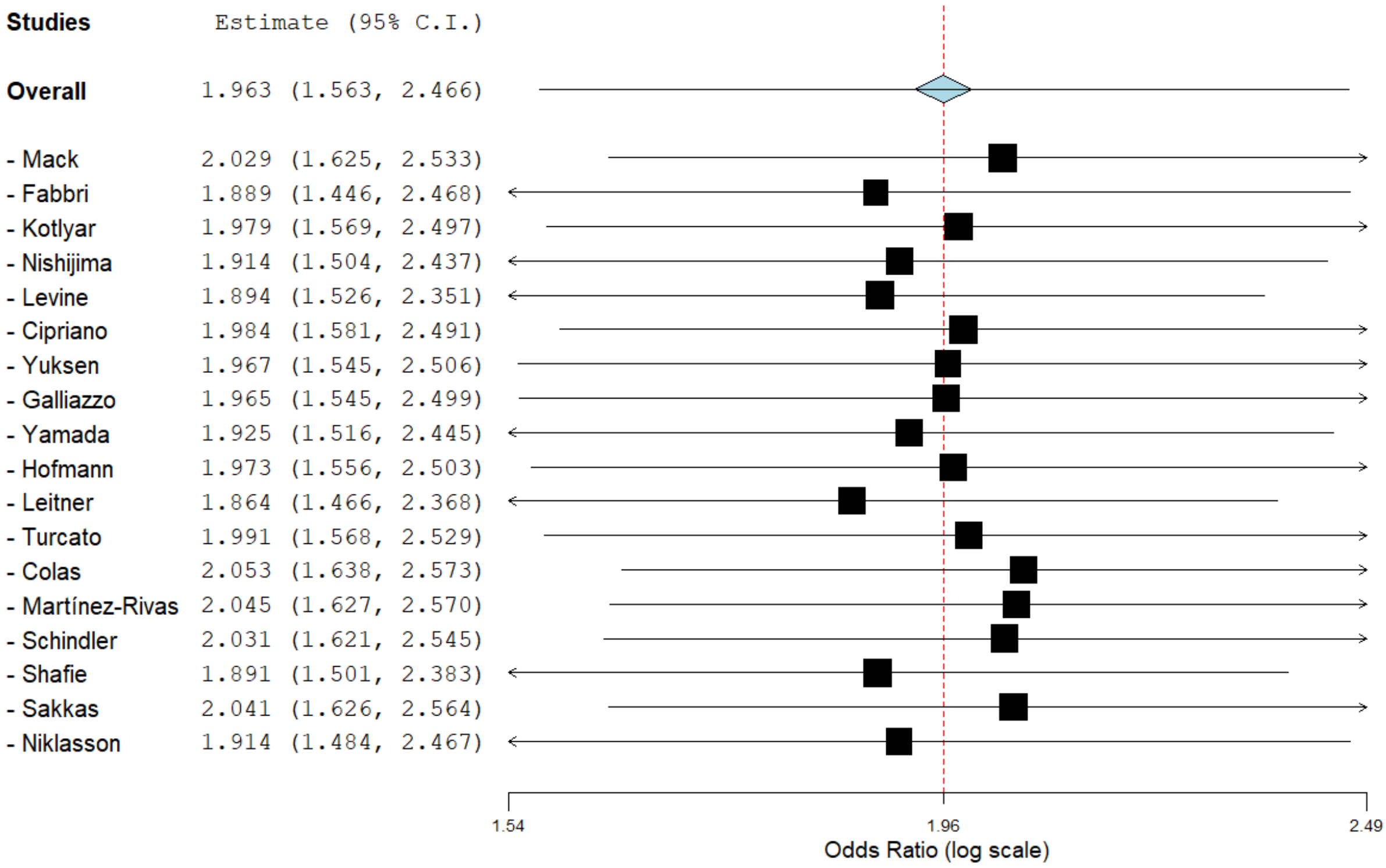

Dual Antiplatelet Therapy (DAPT)

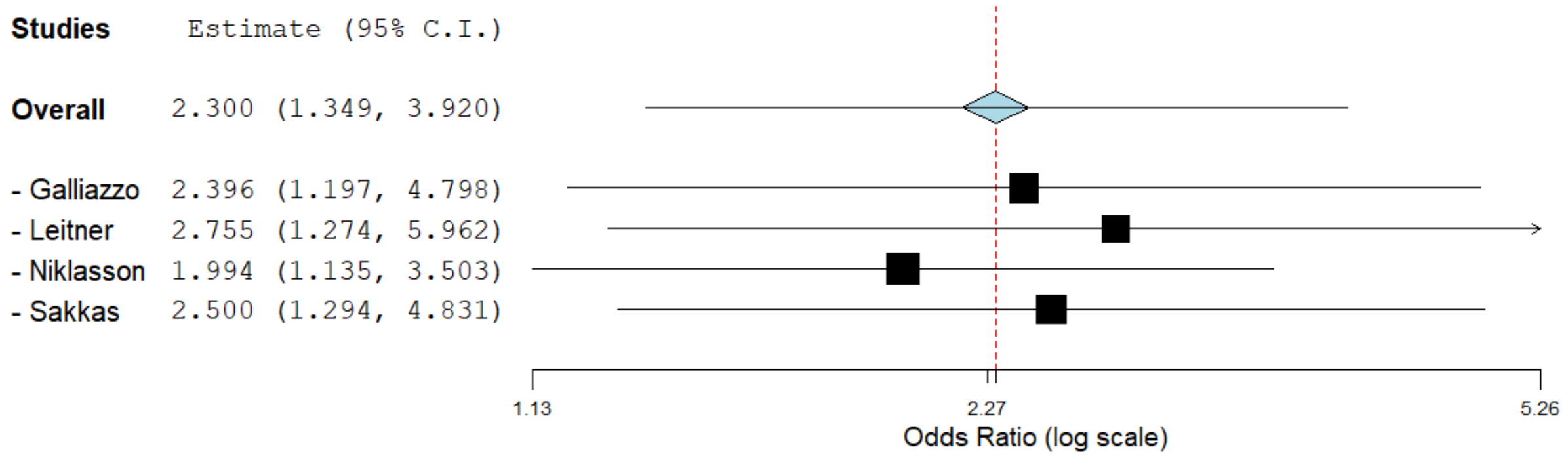

Direct Oral Anticoagulation (DOAC)

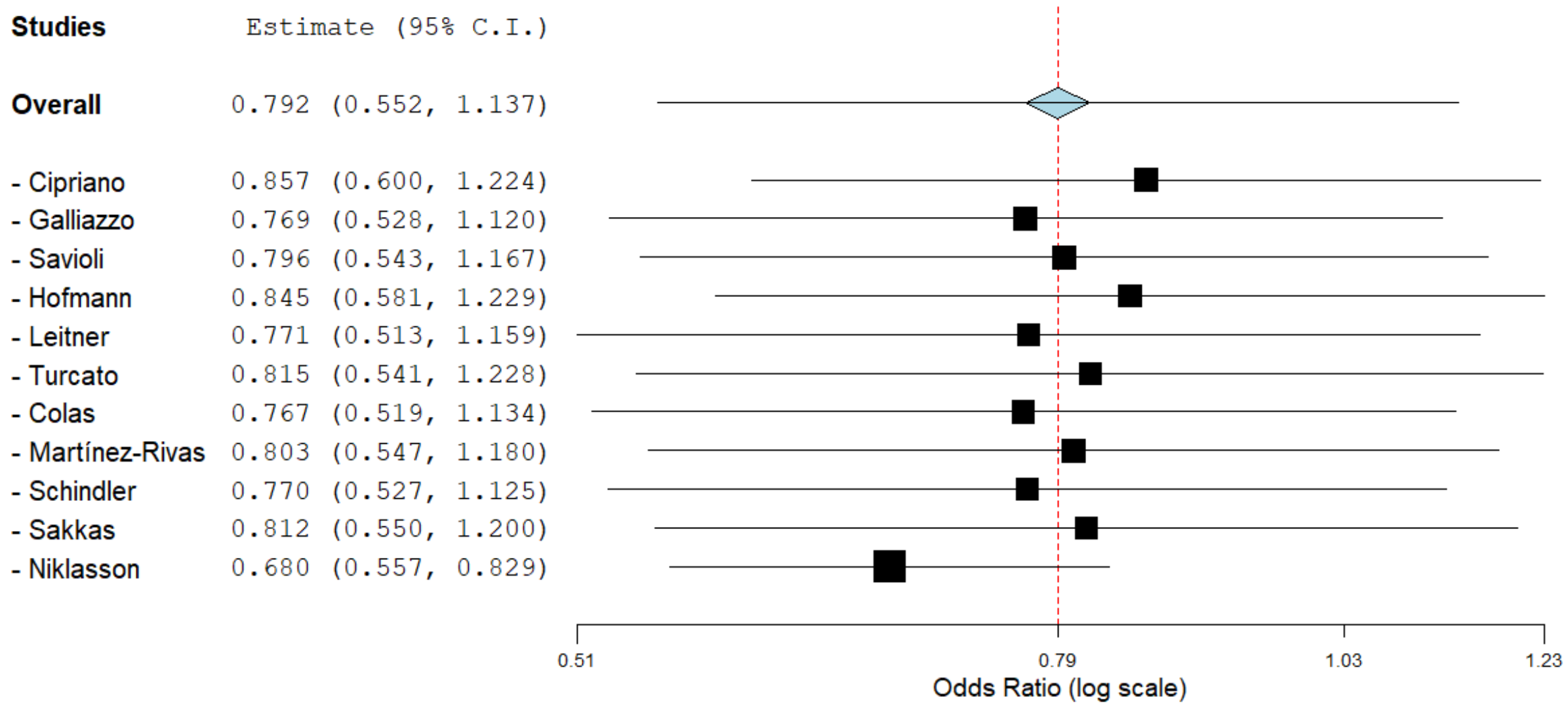

Glasgow Coma Scale Score <15

Studies	Estimate (95% C.I.)
**Overall**	4.884 (3.141, 7.594)
- Livingston	4.811 (3.076, 7.525)
- Shackford	5.056 (3.255, 7.852)
- Harad	5.051 (3.228, 7.903)
- Stein	4.880 (3.081, 7.729)
- Schynoll	4.846 (3.091, 7.597)
- Borczuk	4.925 (3.125, 7.763)
- Culotta	4.908 (3.084, 7.811)
- Gomez	5.005 (3.193, 7.846)
- Dunham	4.873 (3.087, 7.693)
- Hsiang	4.964 (3.144, 7.836)
- Stiell	4.890 (3.071, 7.786)
- Mussack	4.625 (2.966, 7.214)
- Abdul Latip	4.843 (3.091, 7.587)
- Mack	5.064 (3.241, 7.914)
- Ibanez	4.943 (3.152, 7.750)
- Stiell 2	4.945 (3.123, 7.833)
- Biberthaler	4.917 (3.125, 7.734)
- Muller	4.904 (3.131, 7.681)
- Turedi	4.809 (3.071, 7.531)
- Fabbri	4.027 (3.211, 5.050)
- Jacobs	4.871 (3.082, 7.700)
- Kotlyar	4.888 (3.121, 7.656)
- Zongo	5.045 (3.221, 7.903)
- Barrow	4.930 (3.157, 7.698)
- Nishijima	4.949 (3.155, 7.764)
- Alrajhi	5.017 (3.205, 7.853)
- Sugimoto	5.096 (3.261, 7.964)
- Yuksen	4.903 (3.119, 7.707)
- Galliazzo	4.743 (3.028, 7.430)
- Vaniyapong	4.939 (3.134, 7.784)
- Turcato	4.951 (3.122, 7.851)
- Colas	5.074 (3.246, 7.932)
- Dupuis	4.421 (2.843, 6.877)
- Hosseininejad	4.763 (3.051, 7.435)
- Shafie	4.578 (2.936, 7.138)
- Singh	5.008 (3.203, 7.831)
- Chayoua	5.004 (3.192, 7.844)
- Sakkas	4.948 (3.159, 7.750)

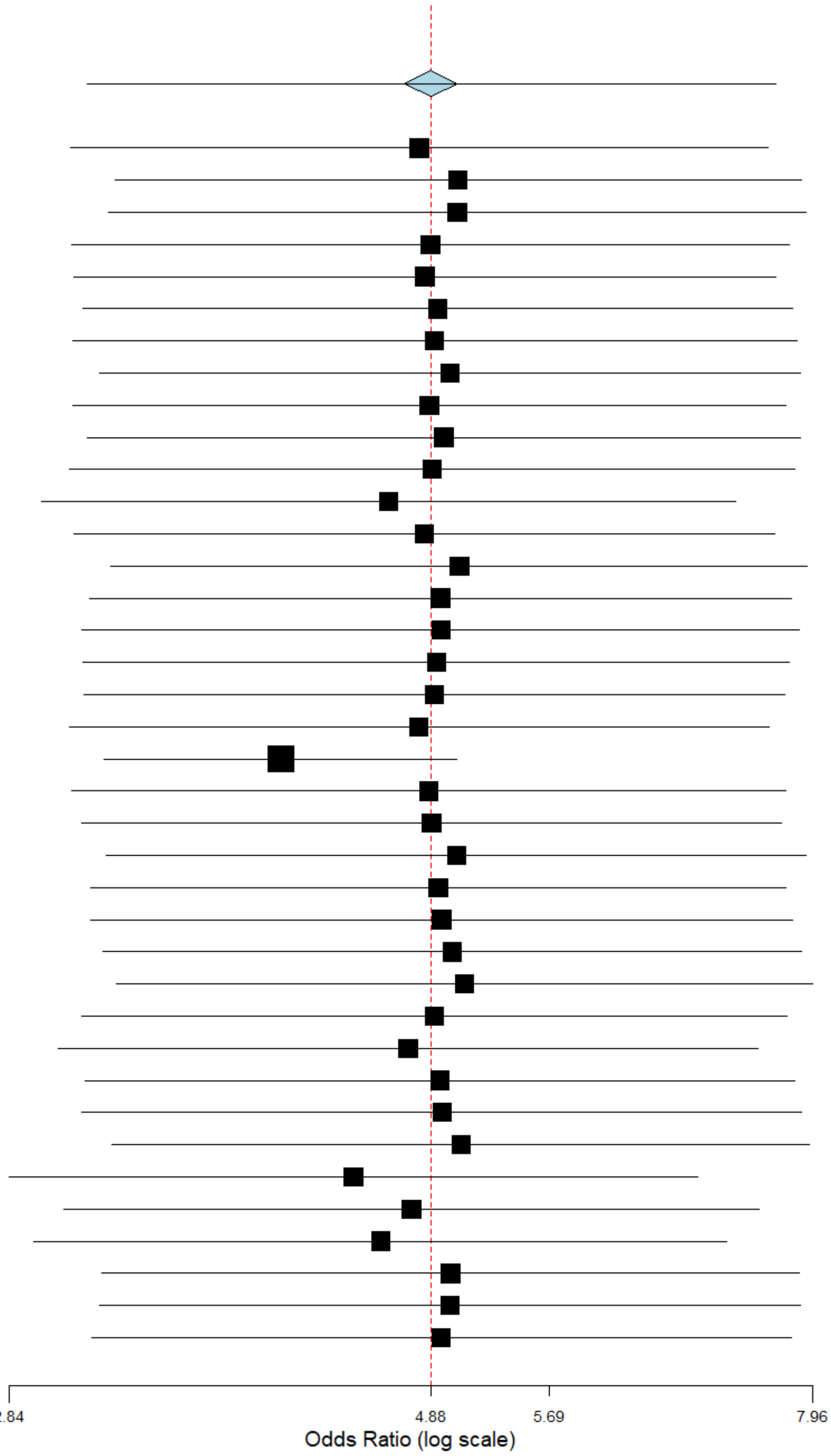

Headache

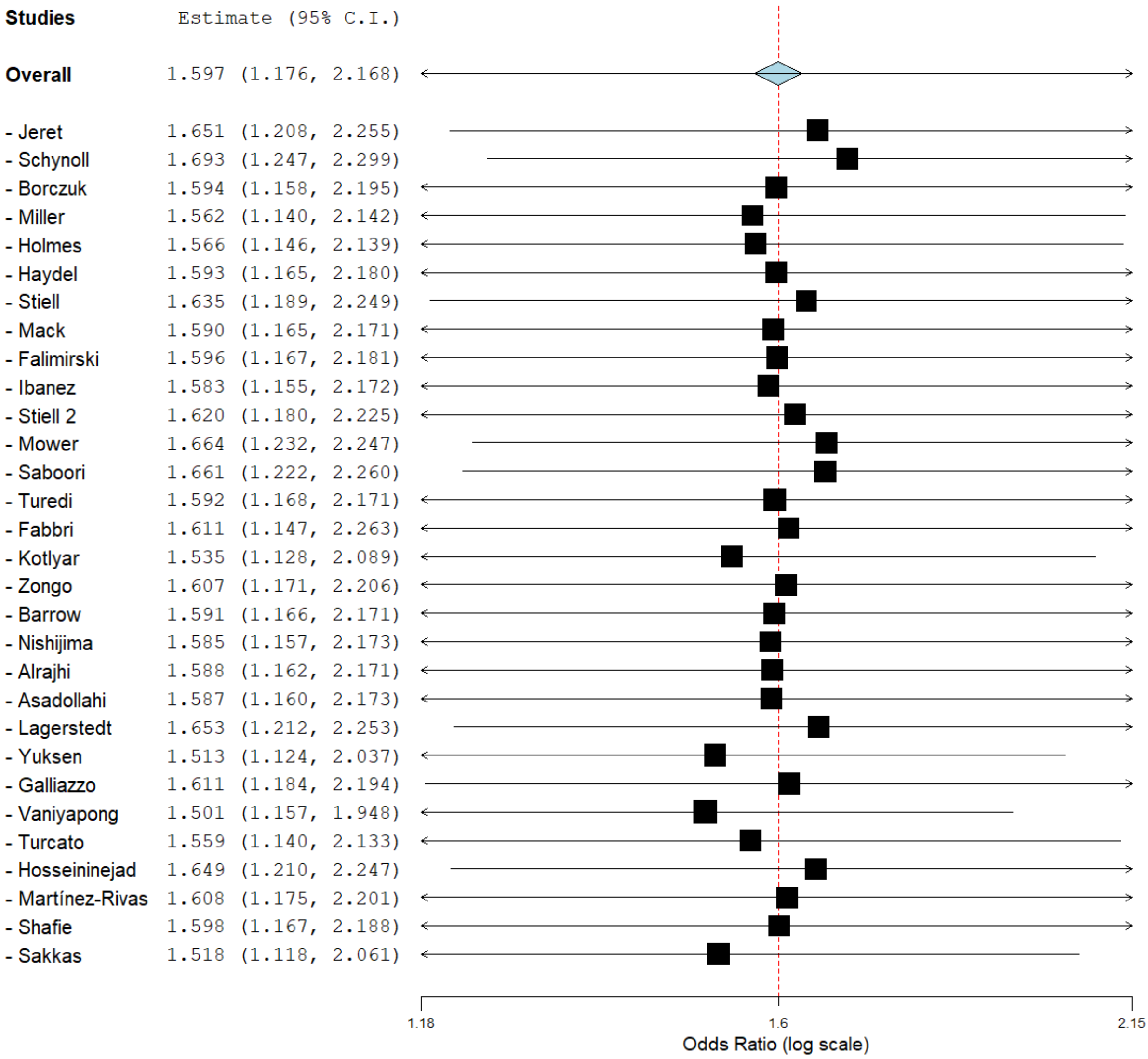
Studies Estimate (95% C.I.)
Overall 1.597 (1.176, 2.168)
- Jeret 1.651 (1.208, 2.255)
- Schynoll 1.693 (1.247, 2.299)
- Borczuk 1.594 (1.158, 2.195)
- Miller 1.562 (1.140, 2.142)
- Holmes 1.566 (1.146, 2.139)
- Haydel 1.593 (1.165, 2.180)
- Stiell 1.635 (1.189, 2.249)
- Mack 1.590 (1.165, 2.171)
- Falimirski 1.596 (1.167, 2.181)
- Ibanez 1.583 (1.155, 2.172)
- Stiell 2 1.620 (1.180, 2.225)
- Mower 1.664 (1.232, 2.247)
- Saboori 1.661 (1.222, 2.260)
- Turedi 1.592 (1.168, 2.171)
- Fabbri 1.611 (1.147, 2.263)
- Kotlyar 1.535 (1.128, 2.089)
- Zongo 1.607 (1.171, 2.206)
- Barrow 1.591 (1.166, 2.171)
- Nishijima 1.585 (1.157, 2.173)
- Alrajhi 1.588 (1.162, 2.171)
- Asadollahi 1.587 (1.160, 2.173)
- Lagerstedt 1.653 (1.212, 2.253)
- Yuksen 1.513 (1.124, 2.037)
- Galliazzo 1.611 (1.184, 2.194)
- Vaniyapong 1.501 (1.157, 1.948)
- Turcato 1.559 (1.140, 2.133)
- Hosseininejad 1.649 (1.210, 2.247)
- Martínez-Rivas 1.608 (1.175, 2.201)
- Shafie 1.598 (1.167, 2.188)
- Sakkas 1.518 (1.118, 2.061)
1.18
1.6
2.15
Odds Ratio (log scale)

Intoxication

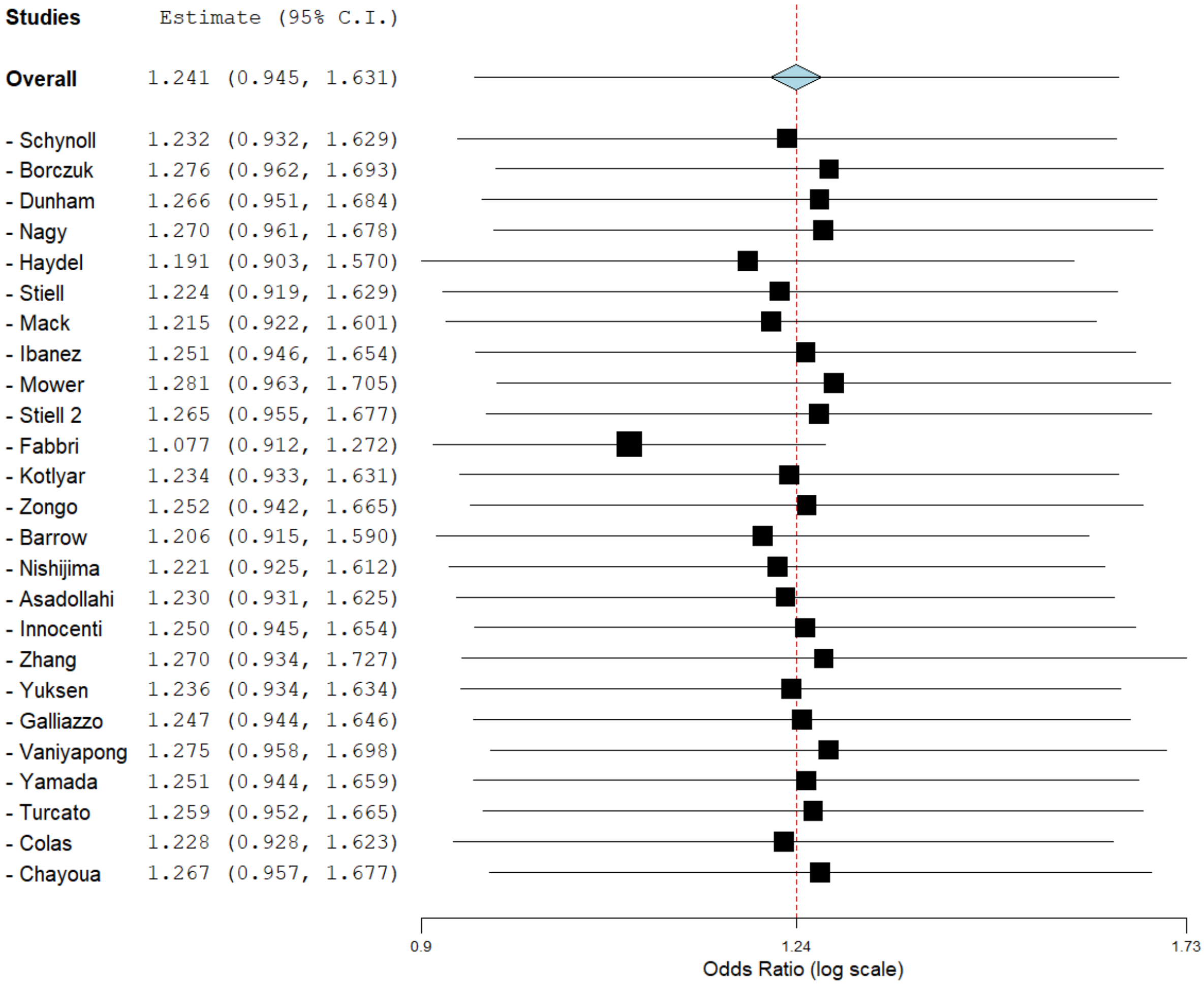

Loss of Consciousness (LOC)

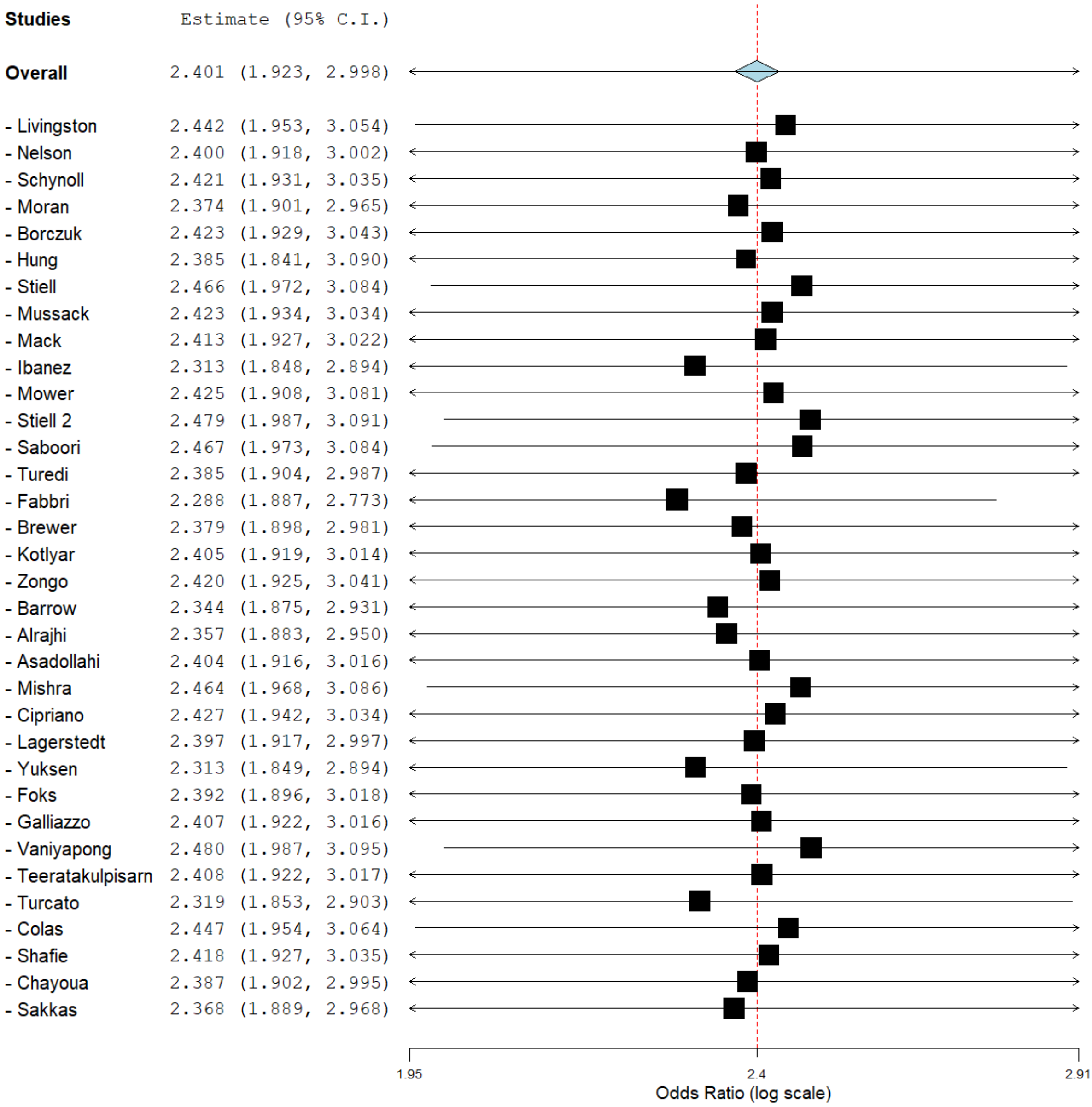

Male Sex

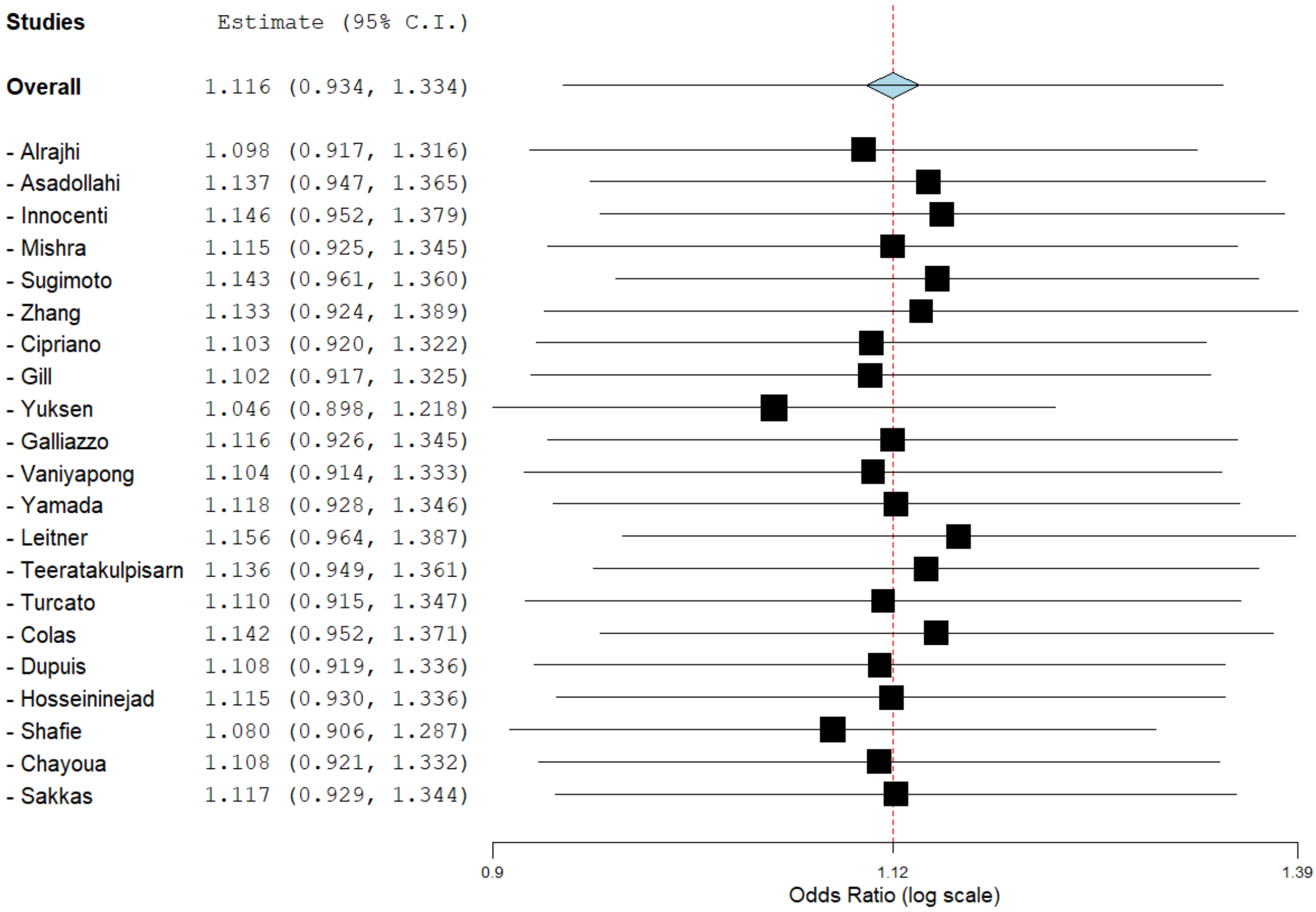

Studies
Estimate (95% C.I.)
Overall 1.116 (0.934, 1.334)
- Alrajhi 1.098 (0.917, 1.316)
- Asadollahi 1.137 (0.947, 1.365)
- Innocenti 1.146 (0.952, 1.379)
- Mishra 1.115 (0.925, 1.345)
- Sugimoto 1.143 (0.961, 1.360)
- Zhang 1.133 (0.924, 1.389)
- Cipriano 1.103 (0.920, 1.322)
- Gill 1.102 (0.917, 1.325)
- Yuksen 1.046 (0.898, 1.218)
- Galliazzo 1.116 (0.926, 1.345)
- Vaniyapong 1.104 (0.914, 1.333)
- Yamada 1.118 (0.928, 1.346)
- Leitner 1.156 (0.964, 1.387)
- Teeratakulpisarn 1.136 (0.949, 1.361)
- Turcato 1.110 (0.915, 1.347)
- Colas 1.142 (0.952, 1.371)
- Dupuis 1.108 (0.919, 1.336)
- Hosseininejad 1.115 (0.930, 1.336)
- Shafie 1.080 (0.906, 1.287)
- Chayoua 1.108 (0.921, 1.332)
- Sakkas 1.117 (0.929, 1.344)
0.9
1.12
1.39
Odds Ratio (log scale)

Neurological Deficit

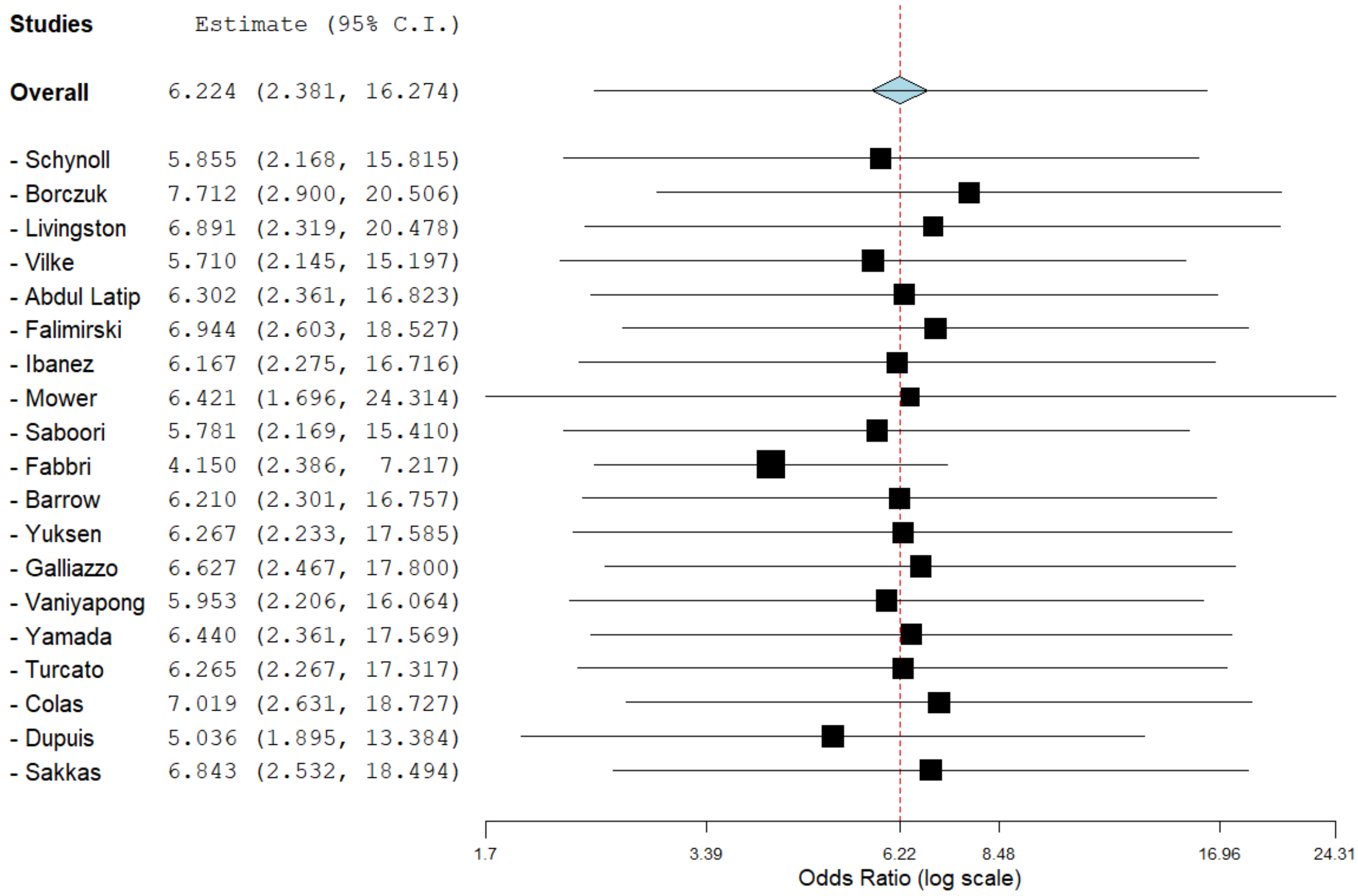

Post-traumatic Amnesia (PTA)

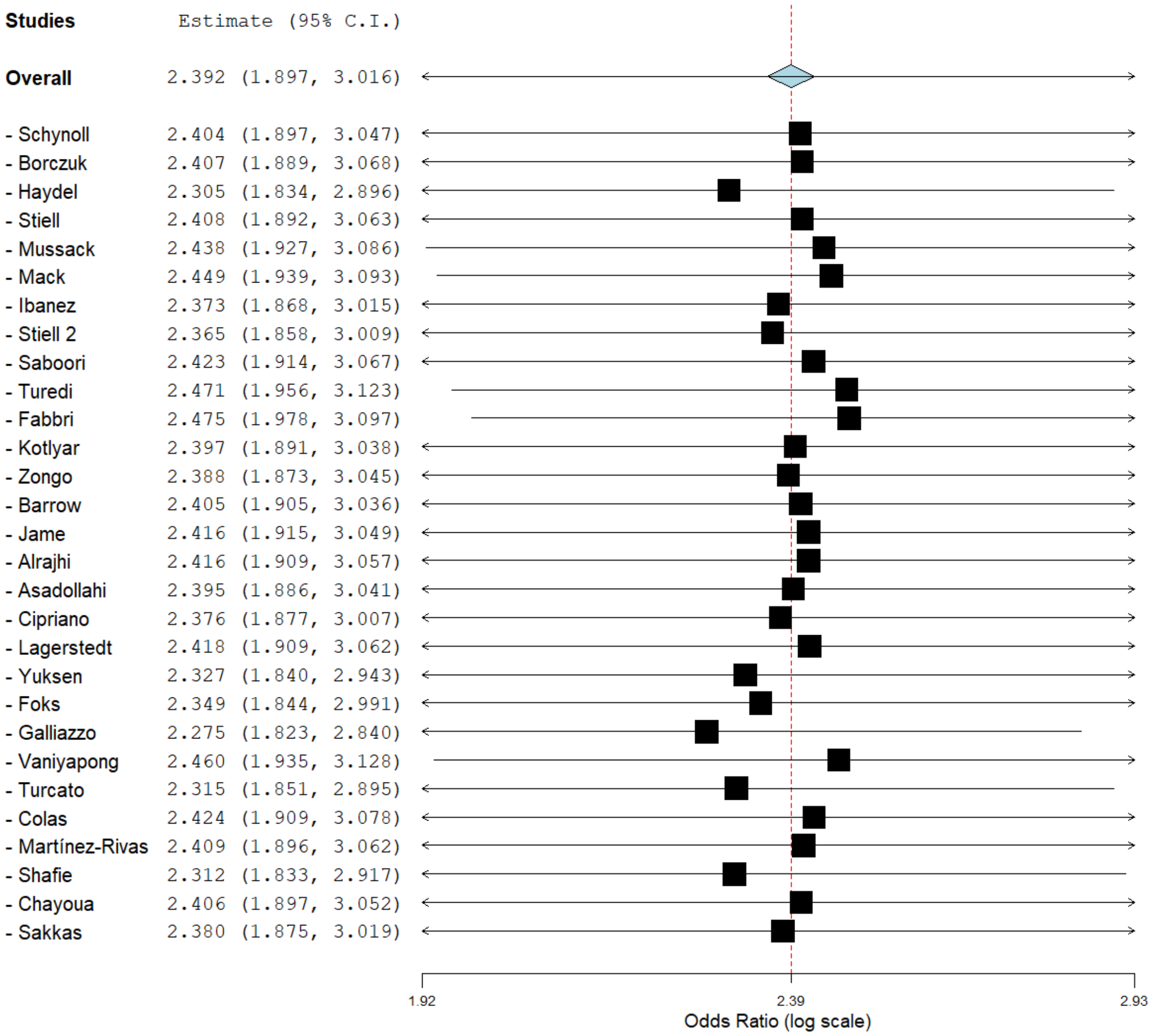

Repeated Vomiting

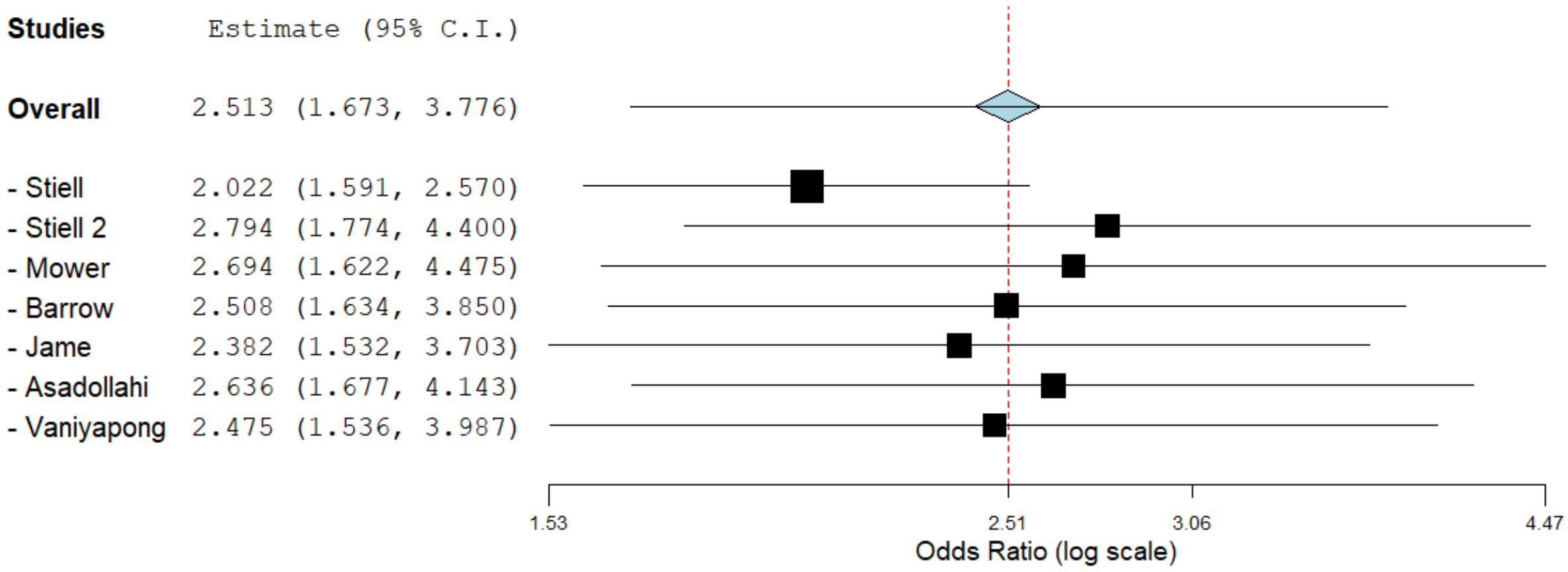

Scalp Wound

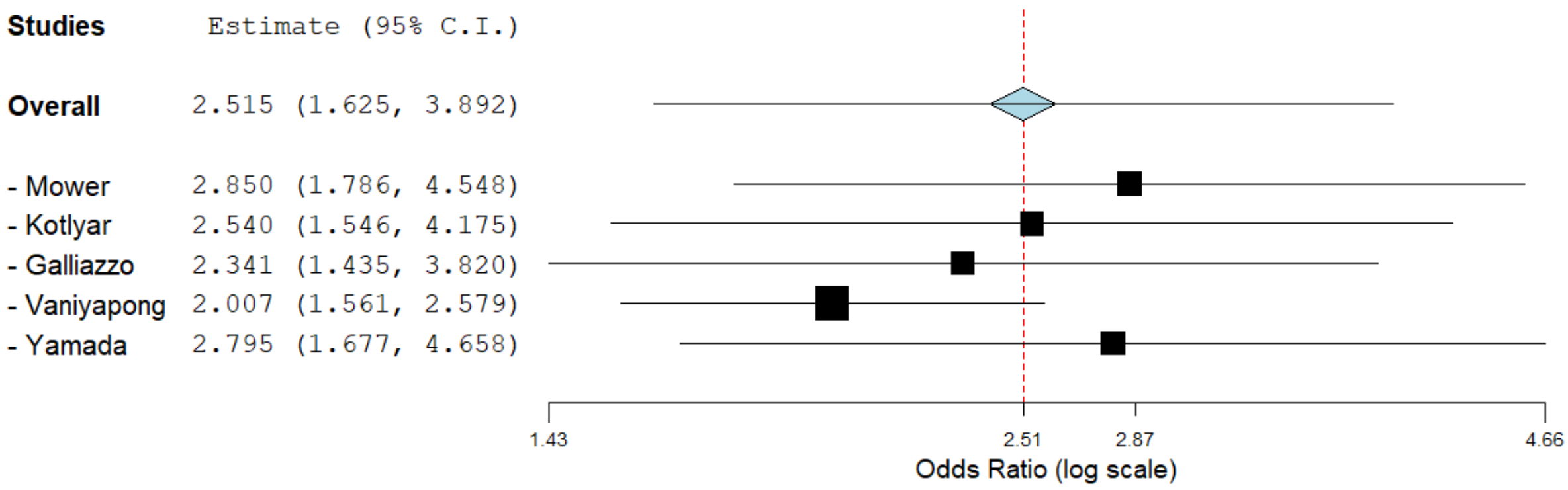

Post-traumatic Seizure

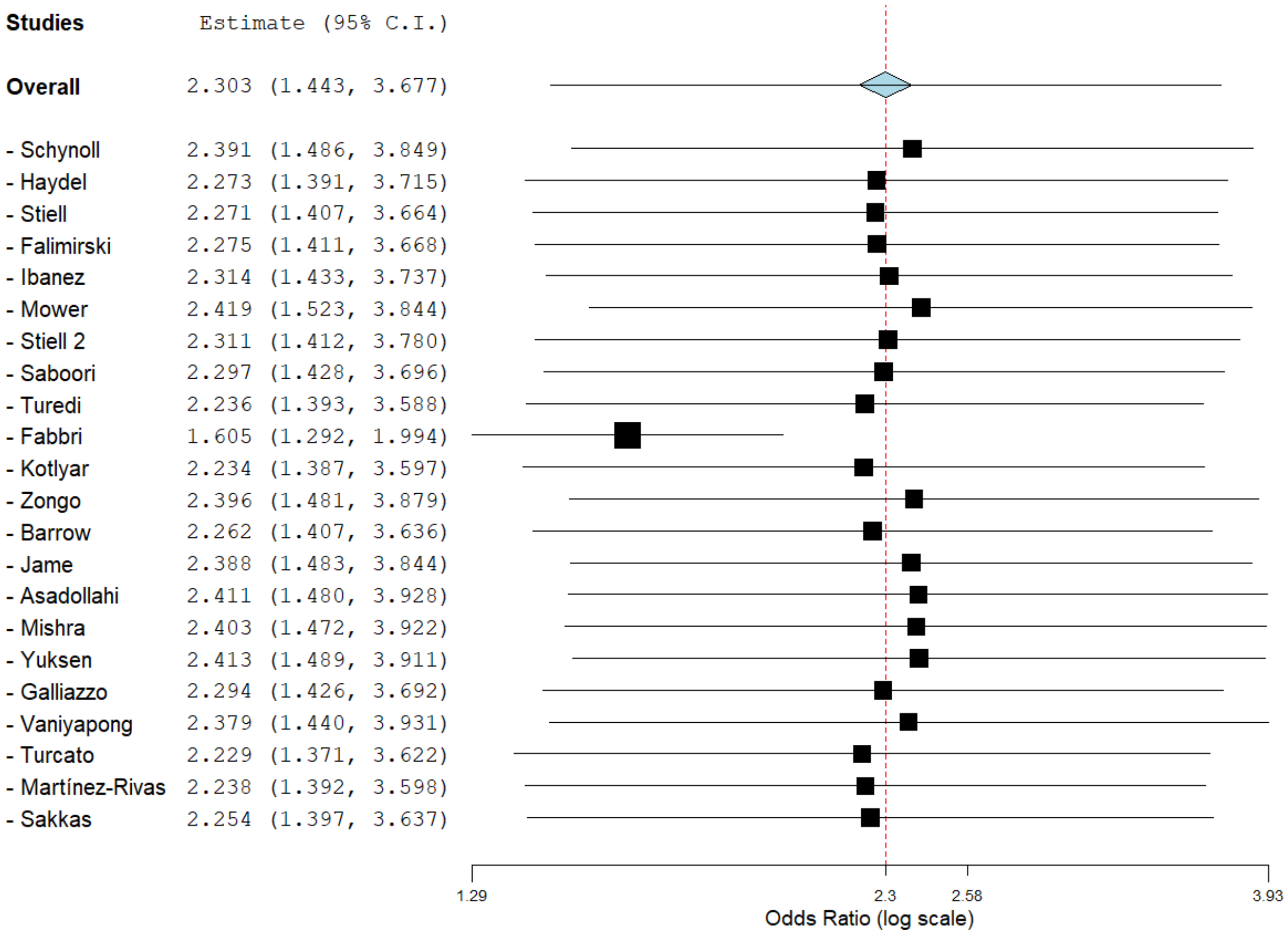

Signs of Injury Above Clavicle

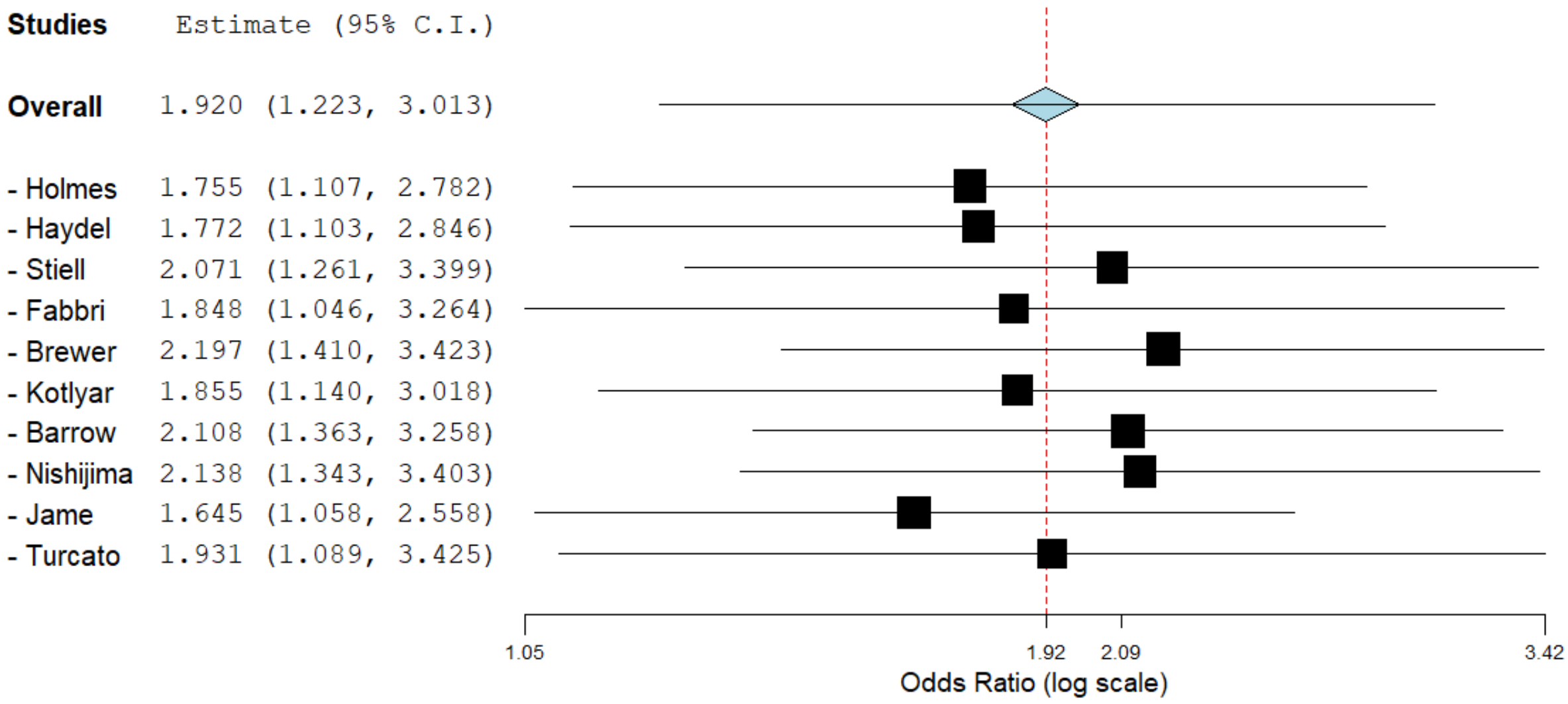

Signs of Skull Fracture

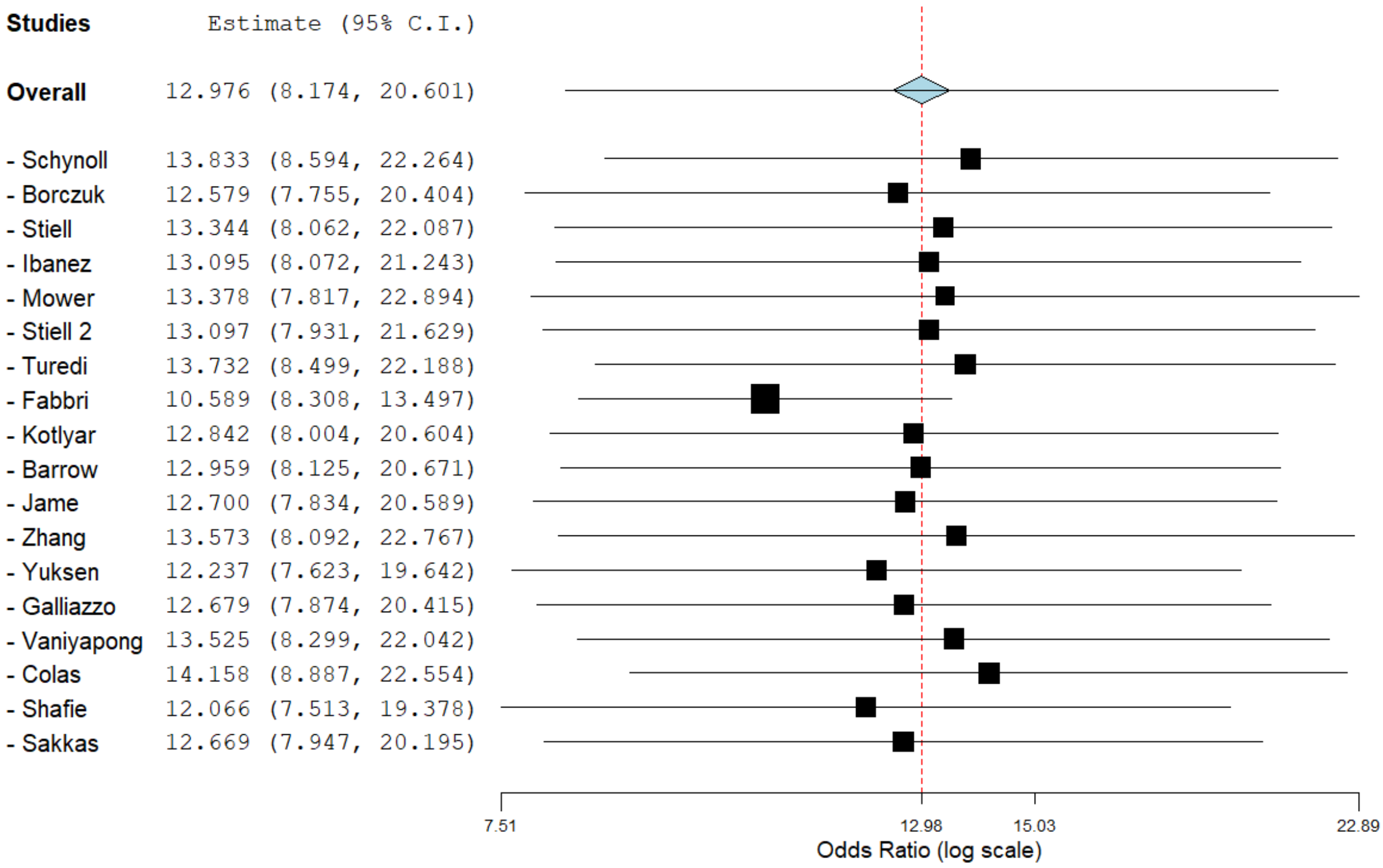

Vitamin K Antagonist

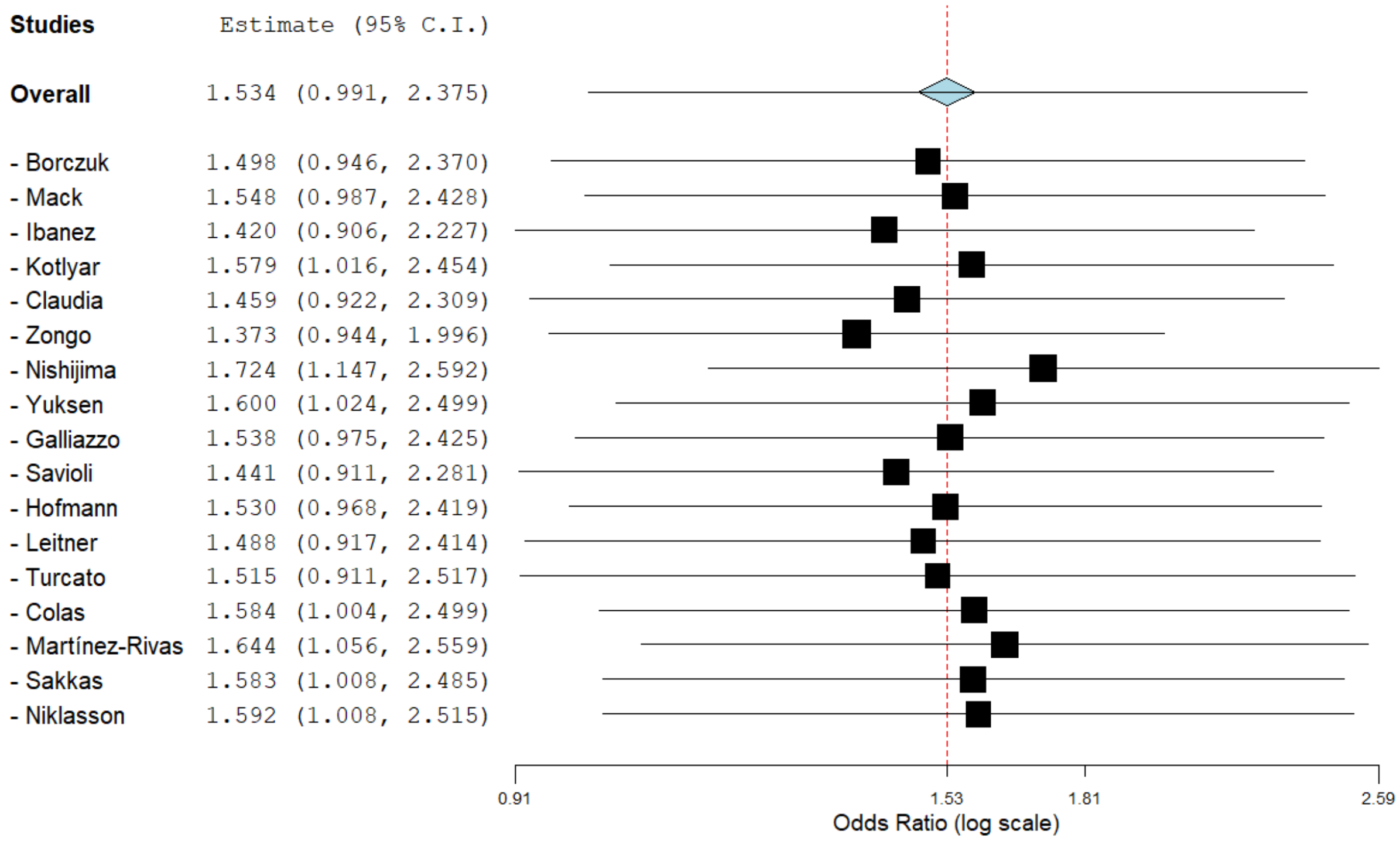

Vomiting Once

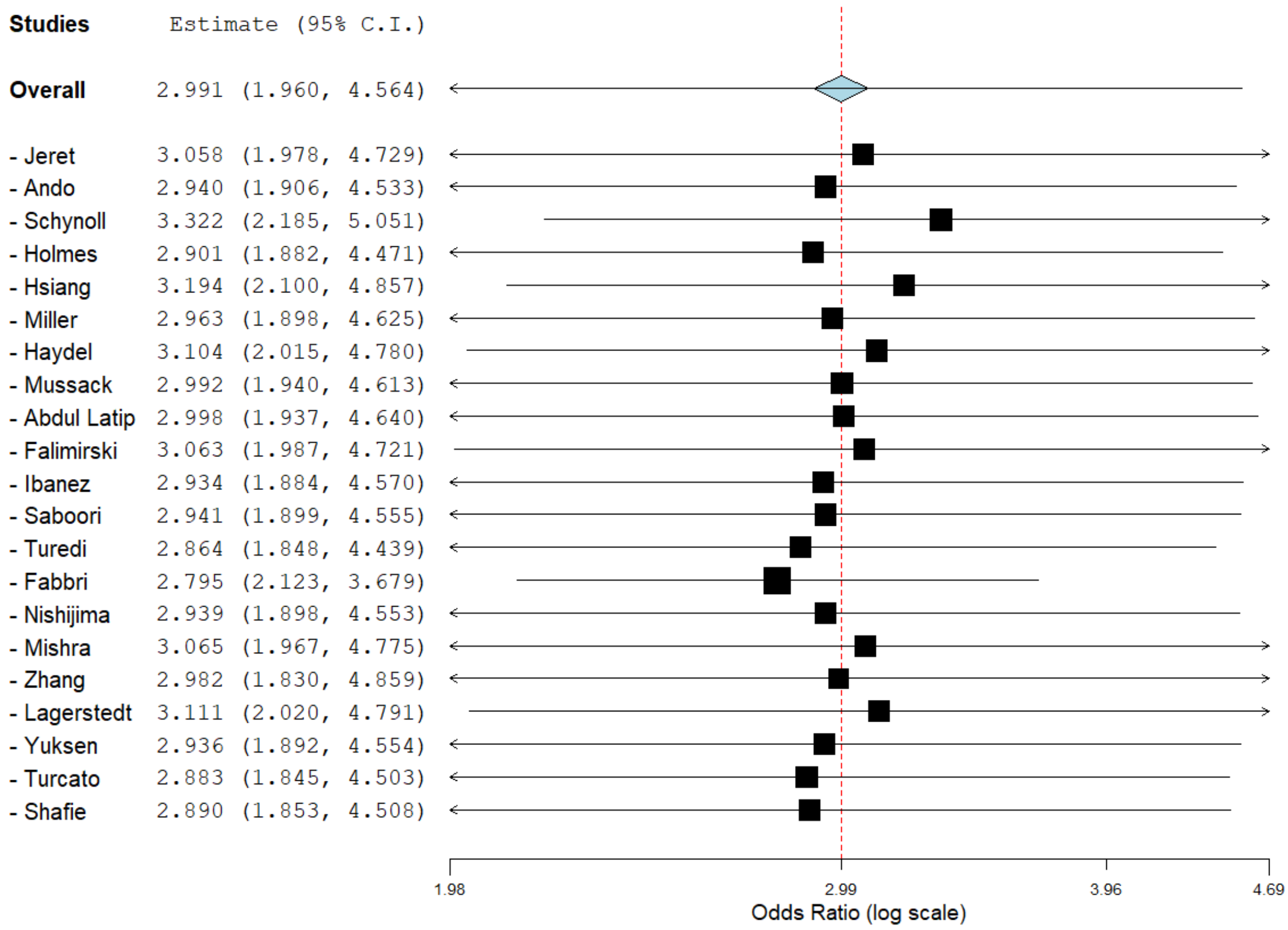

5. Funnel Plots (Traumatic Intracranial Injury)

Age

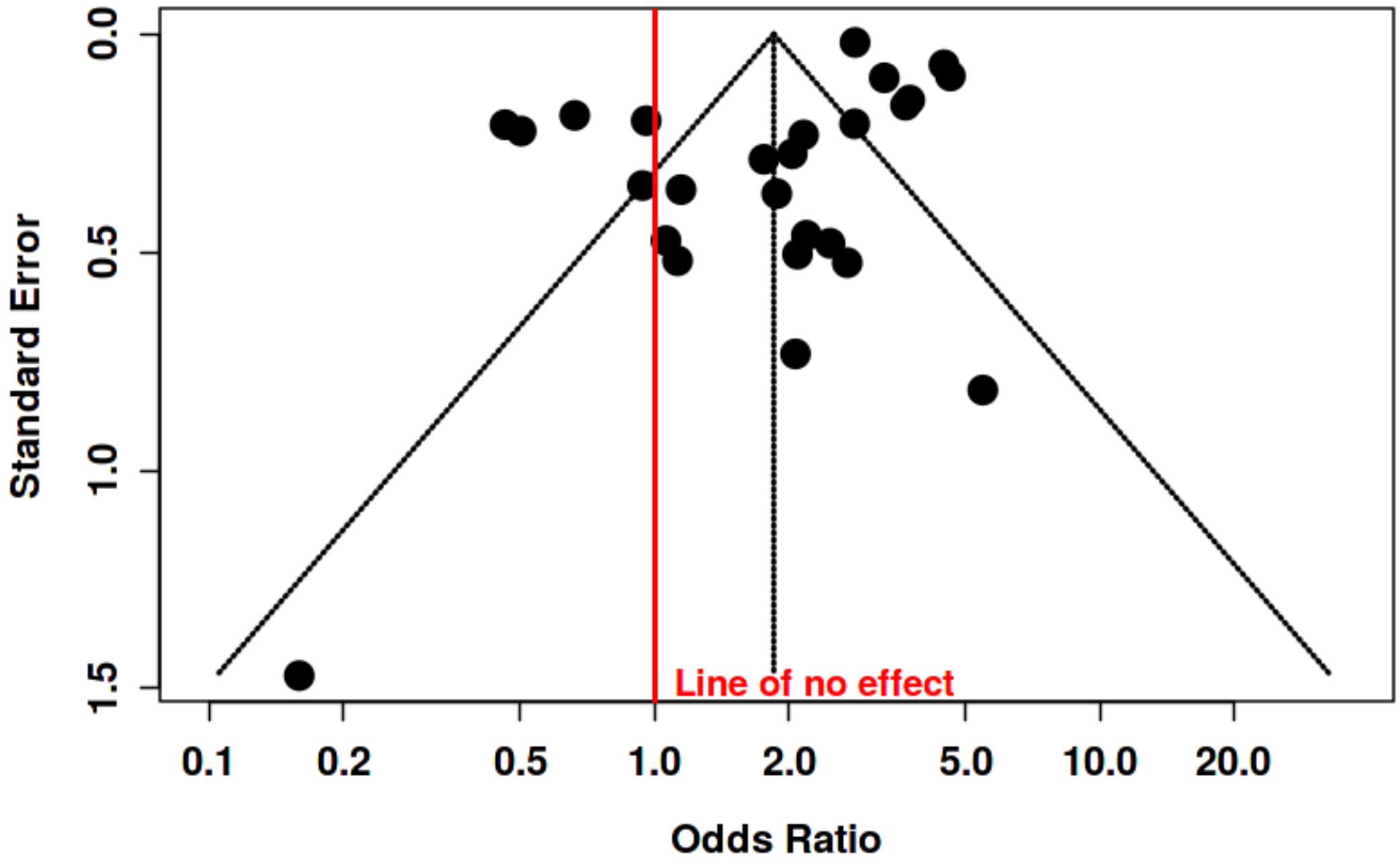

Anticoagulation

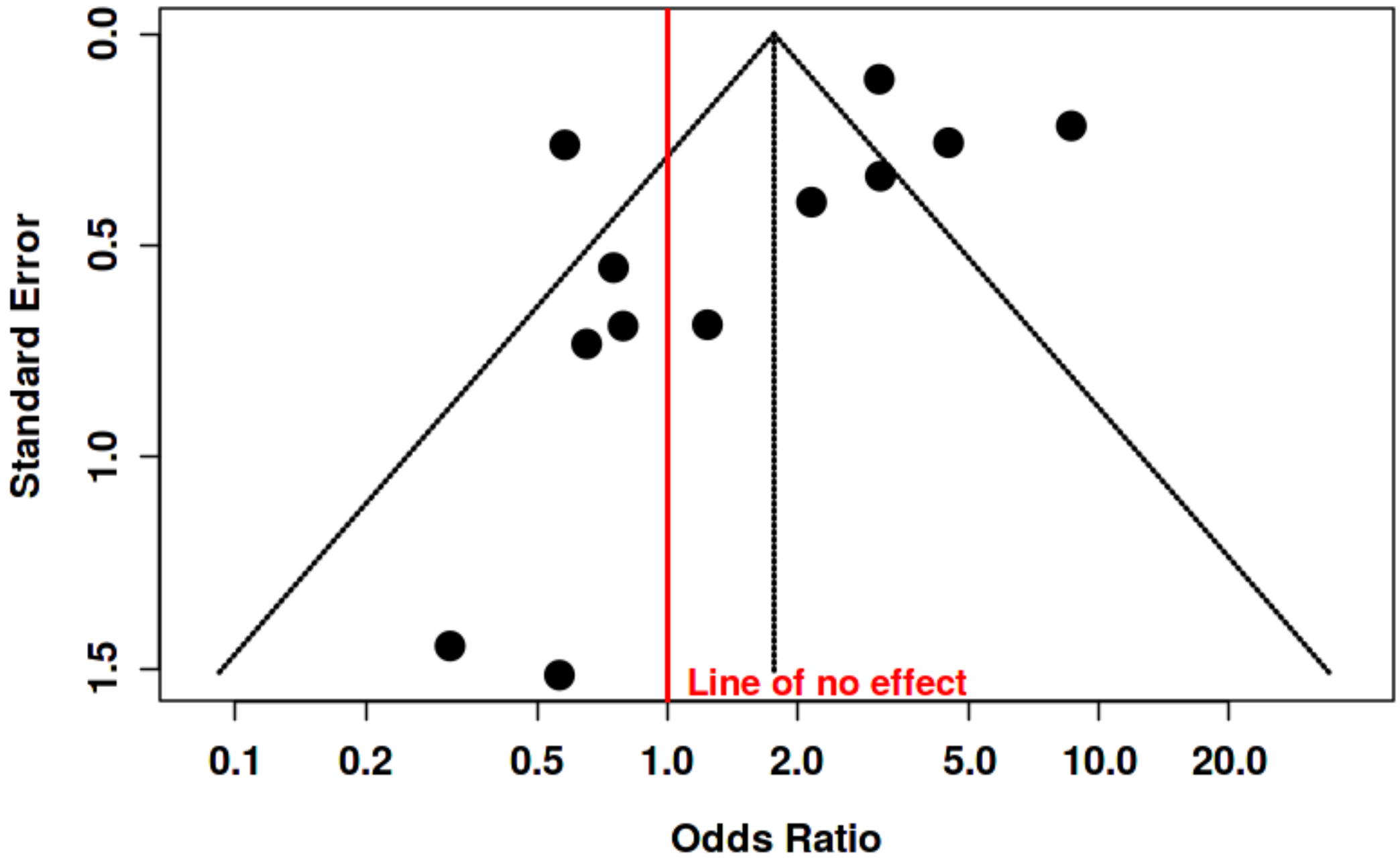

Antiplatelet Therapy

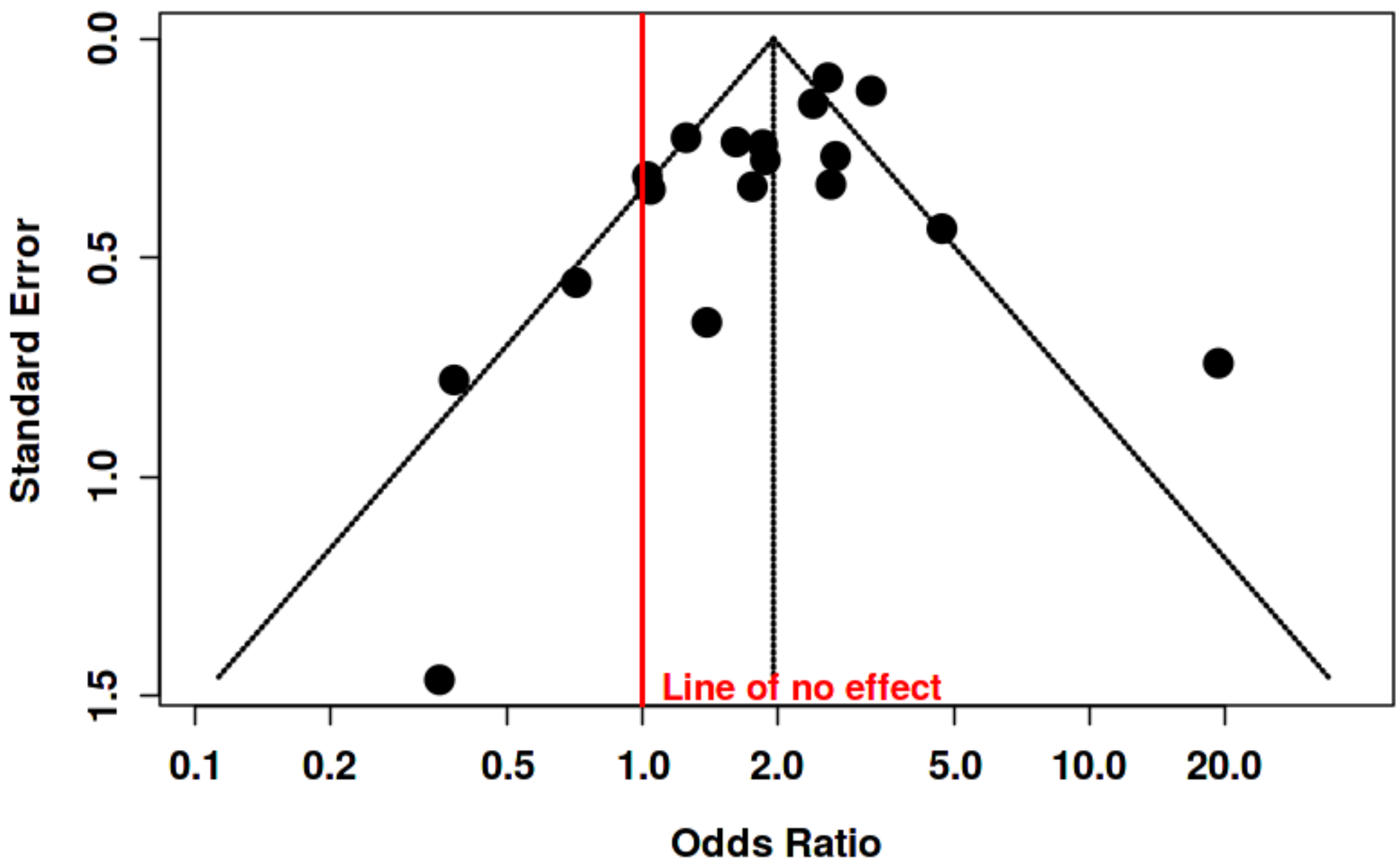

Dual Antiplatelet Therapy (DAPT)

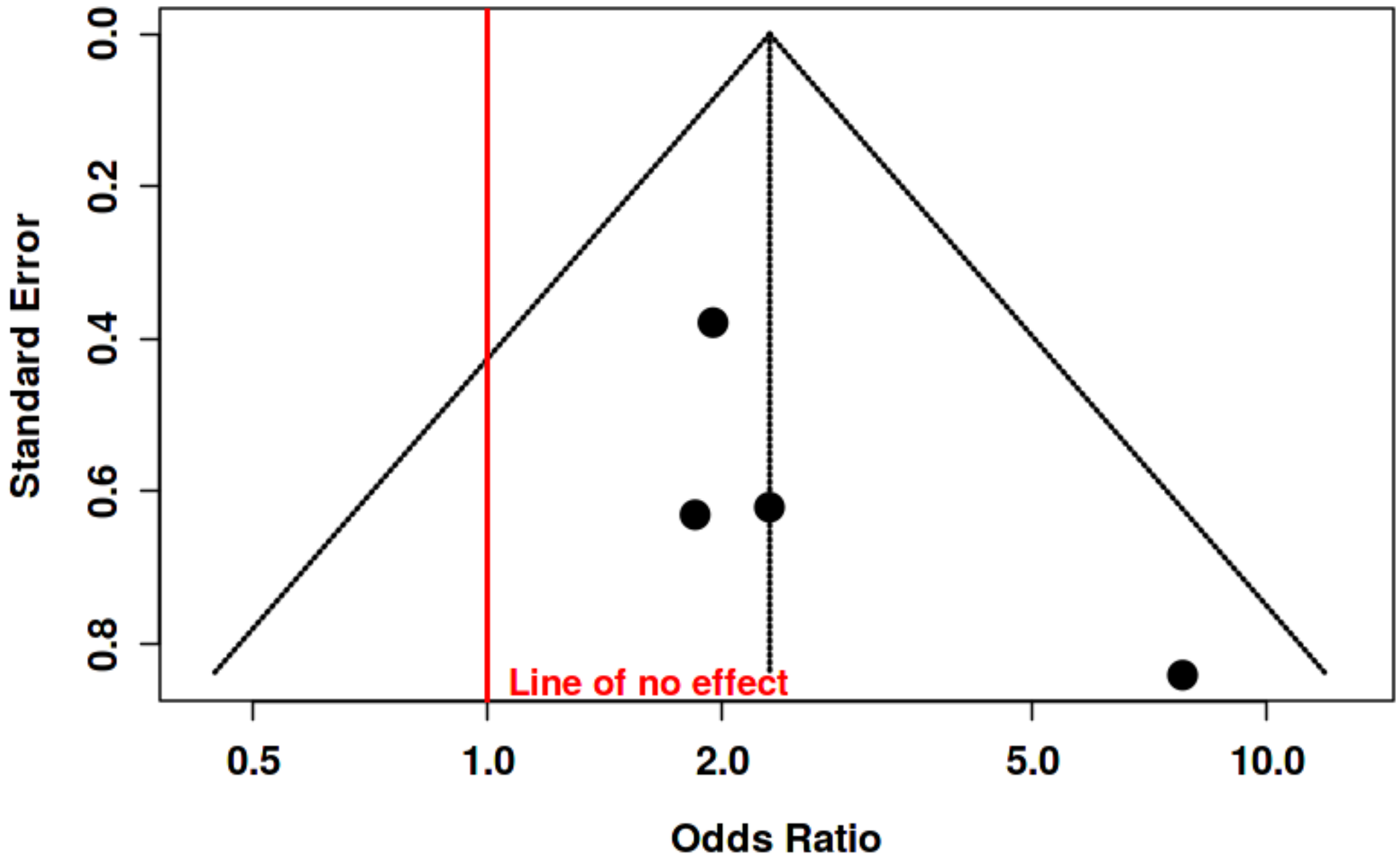

Direct Oral Anticoagulation (DOAC)

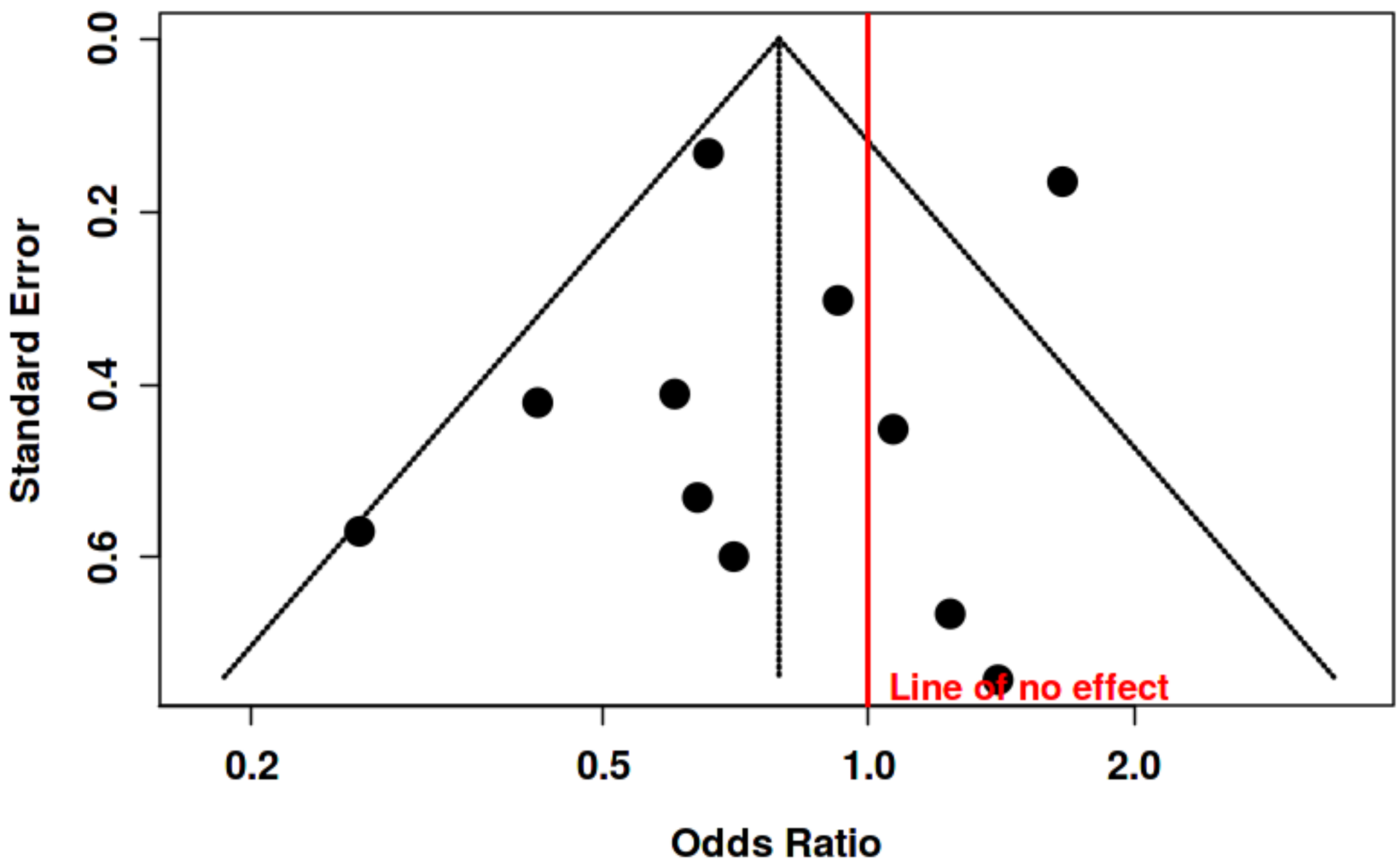

Glasgow Coma Scale Score <15

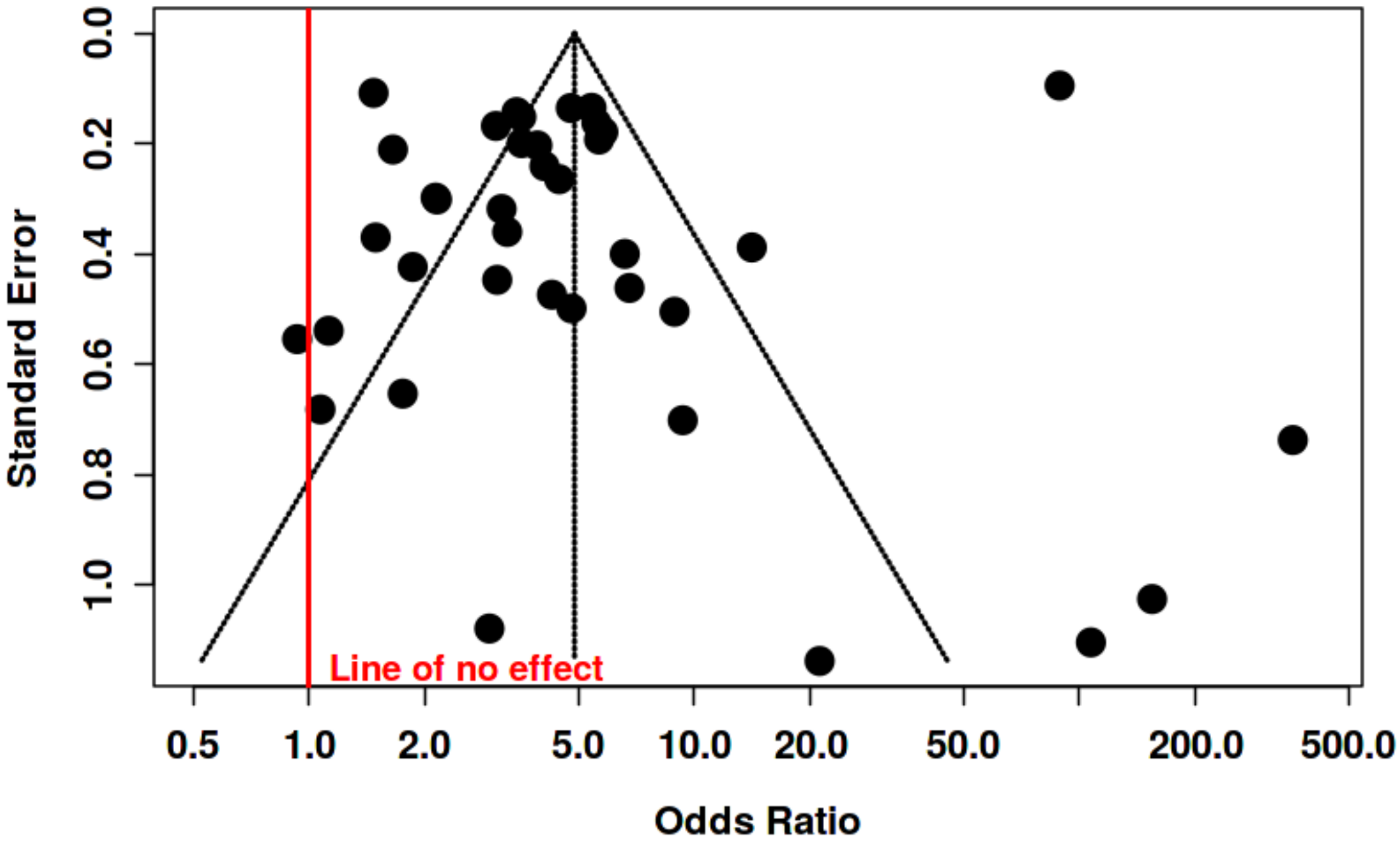

Headache

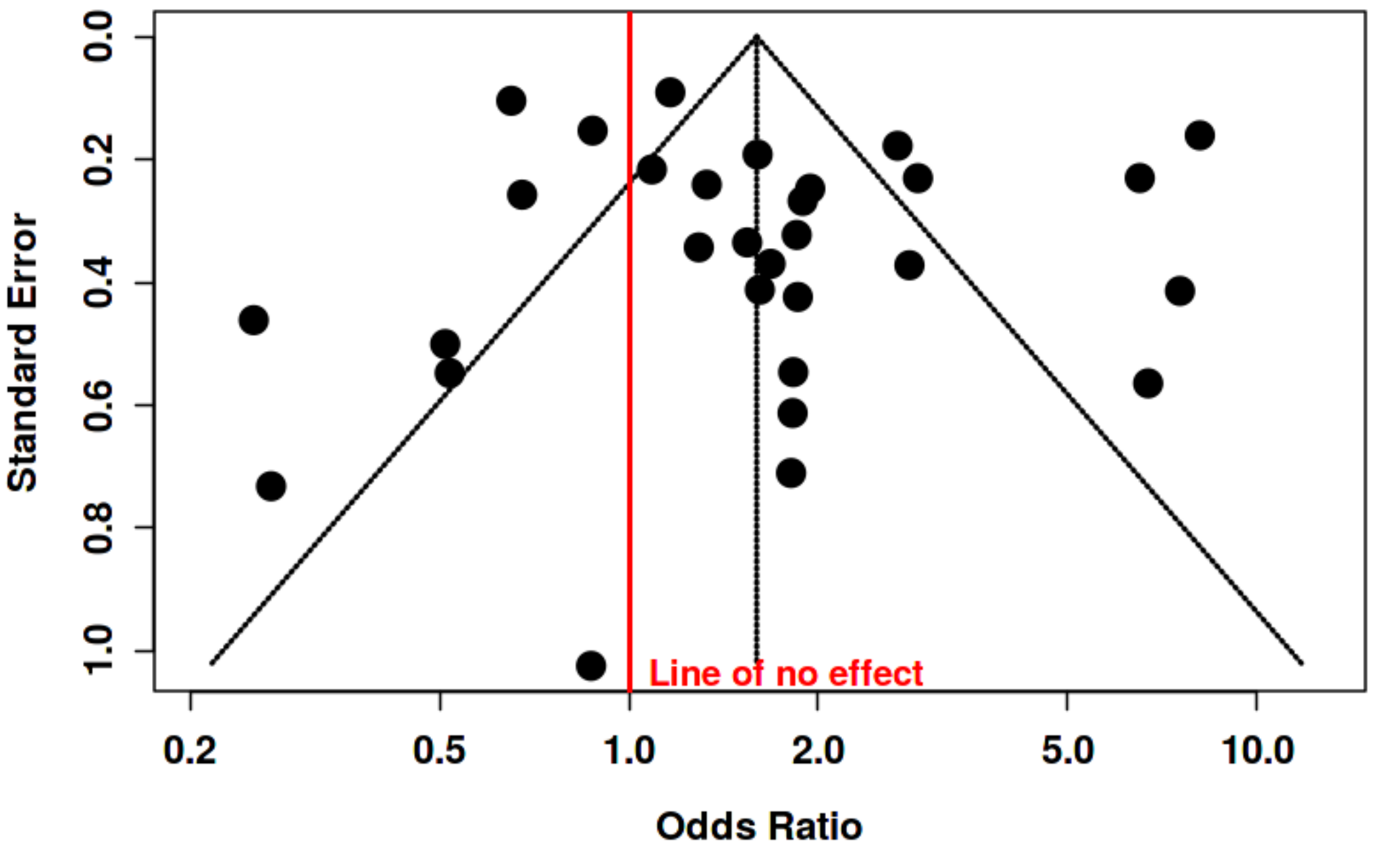

Intoxication

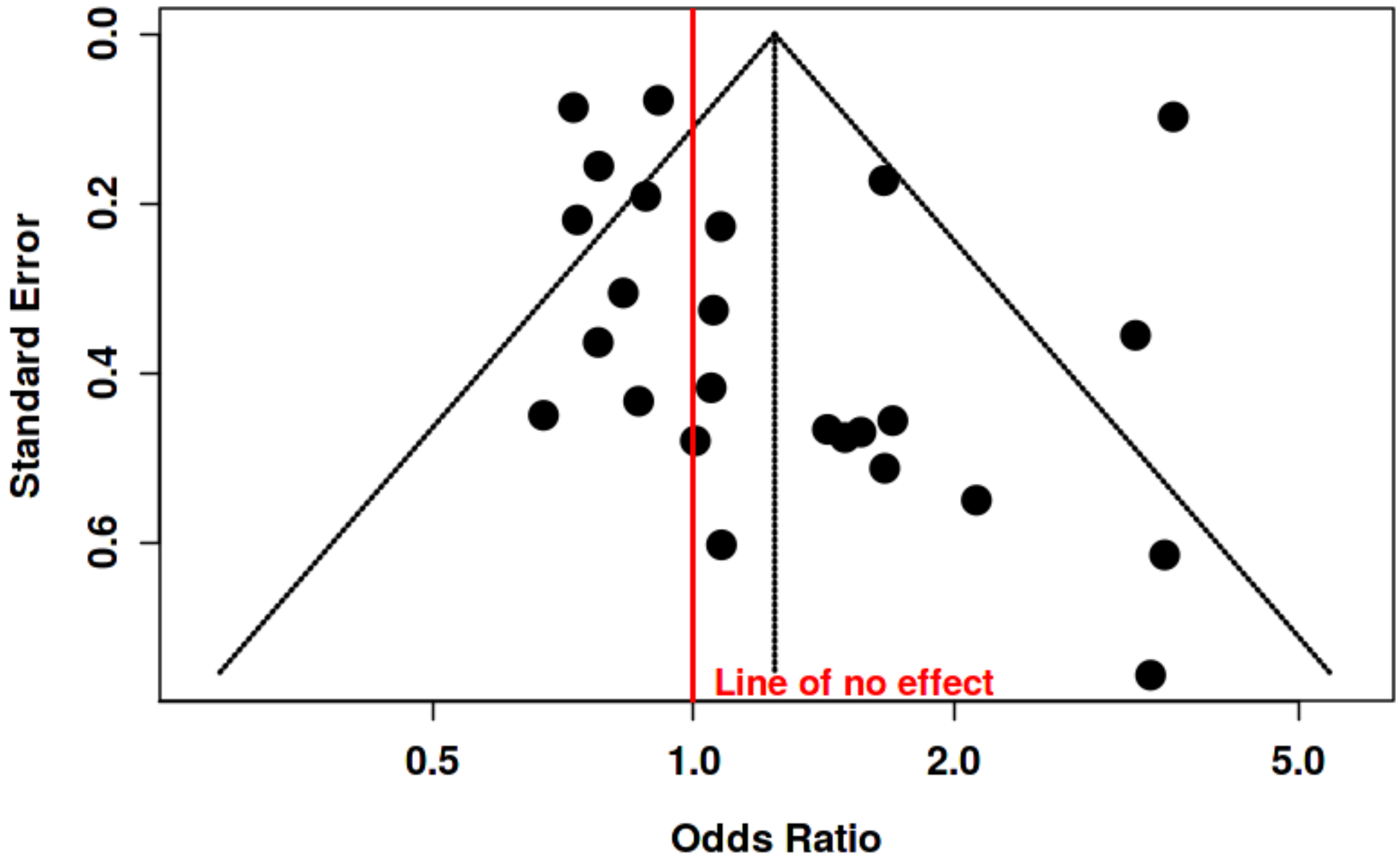

Loss of Consciousness

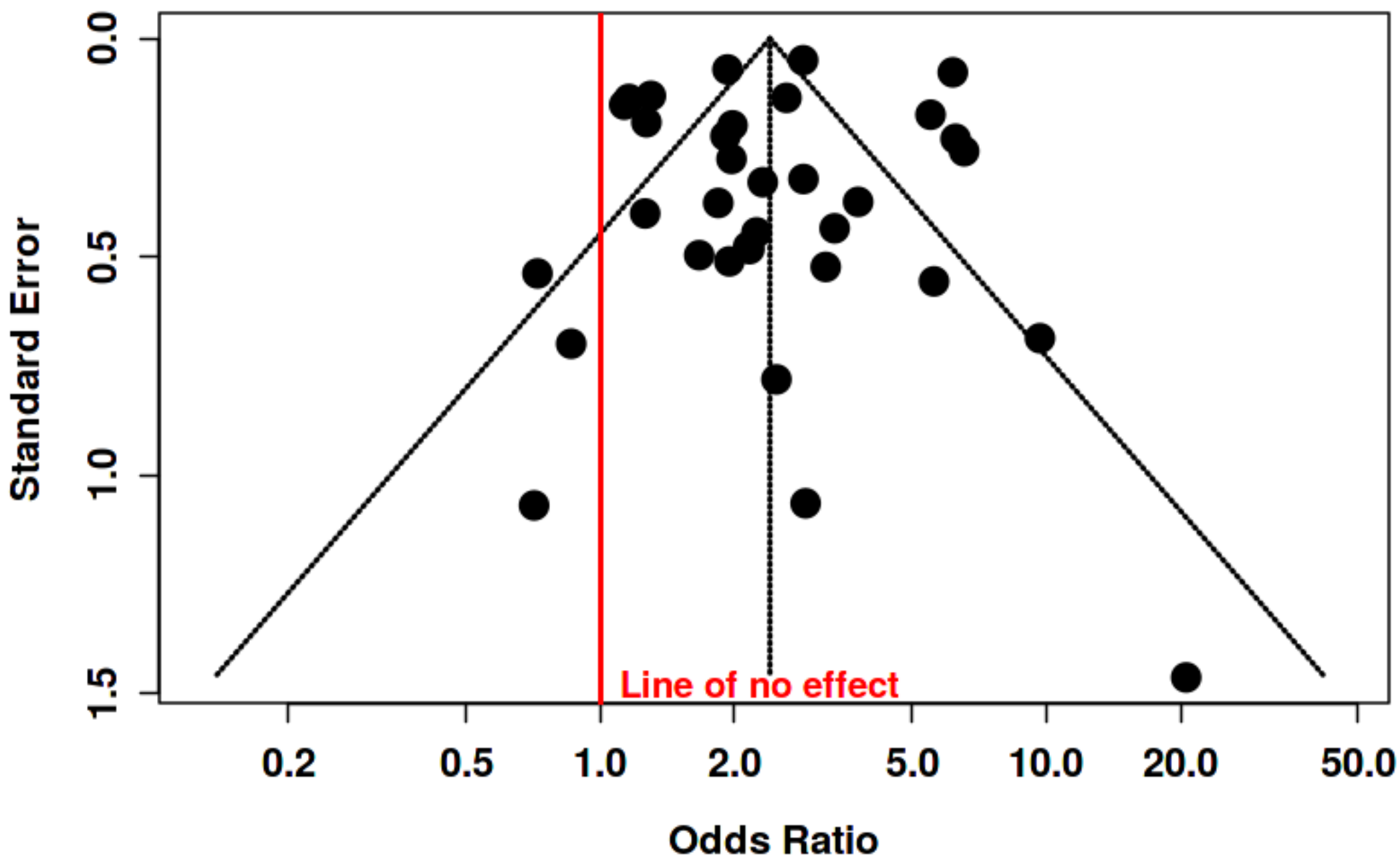

Male Sex

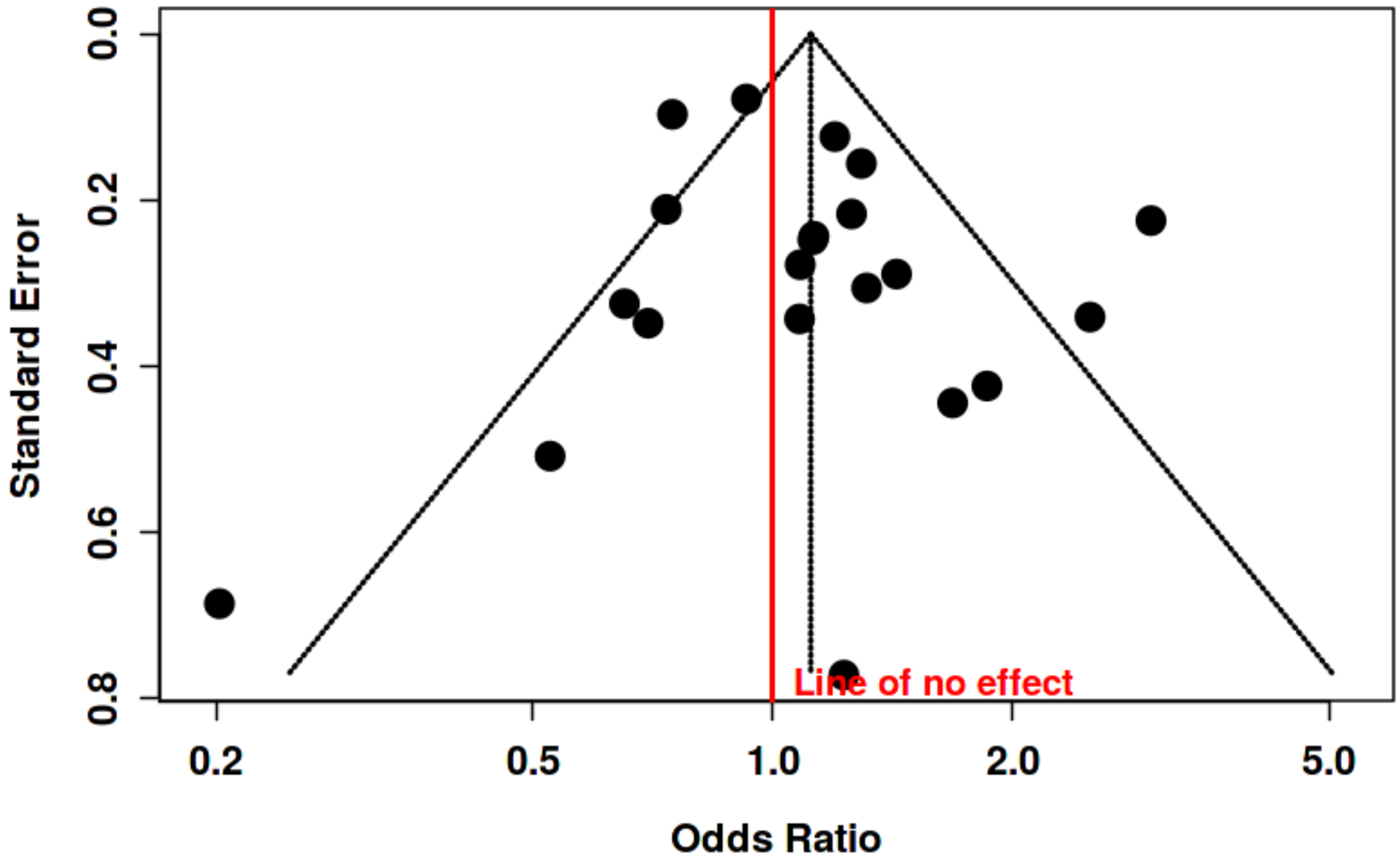

Neurological Deficit

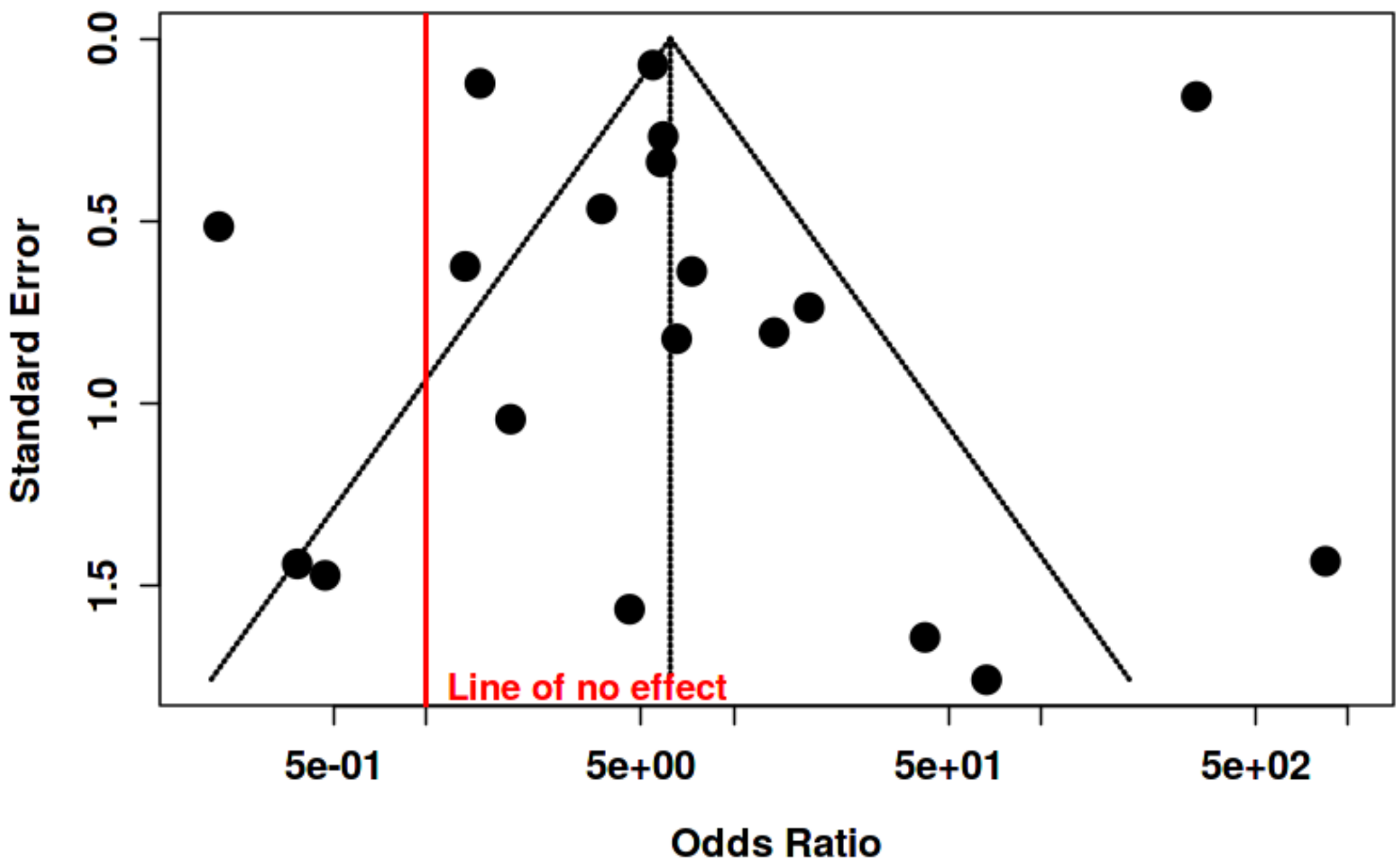

Post-traumatic Amnesia

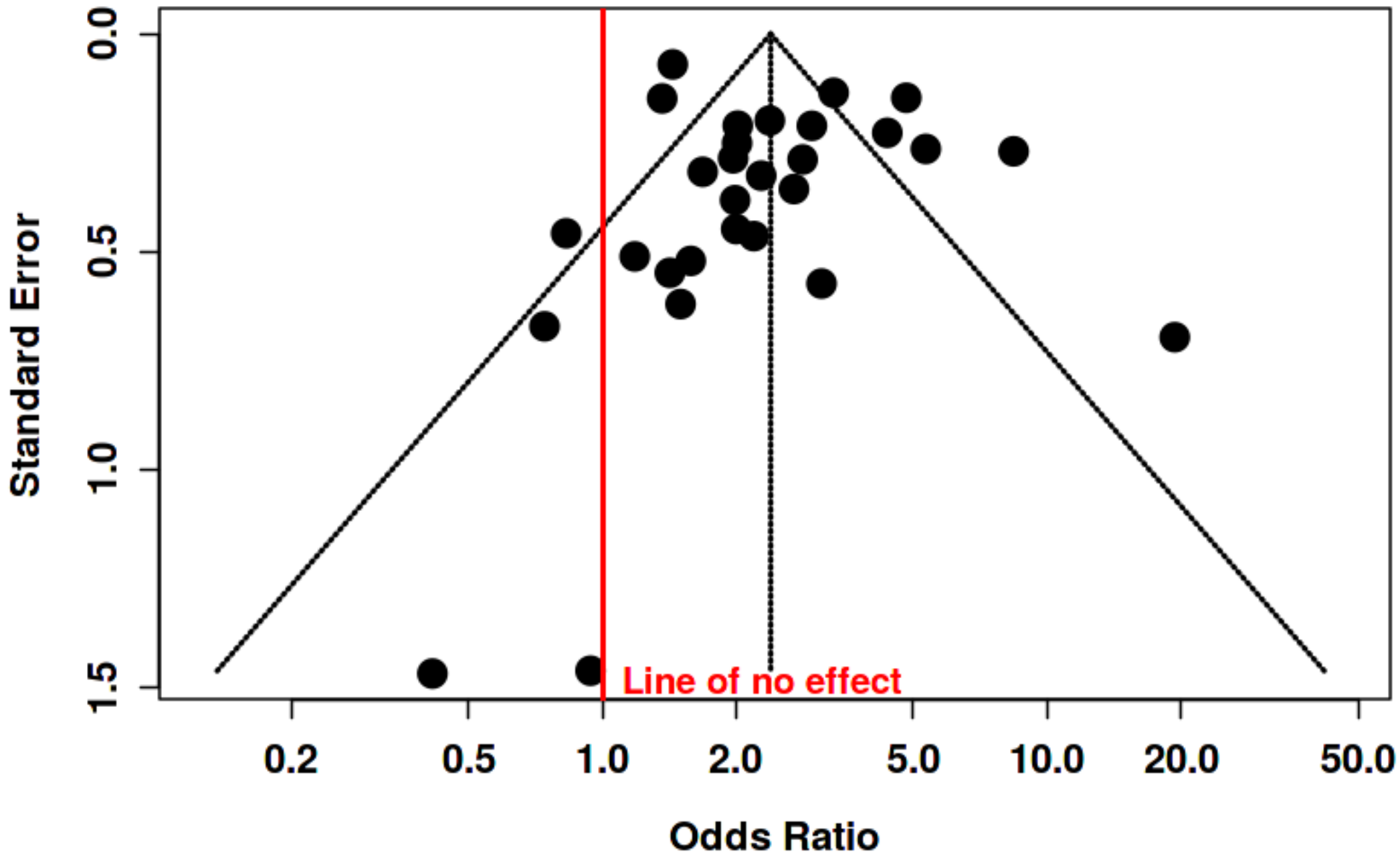

Repeated Vomiting

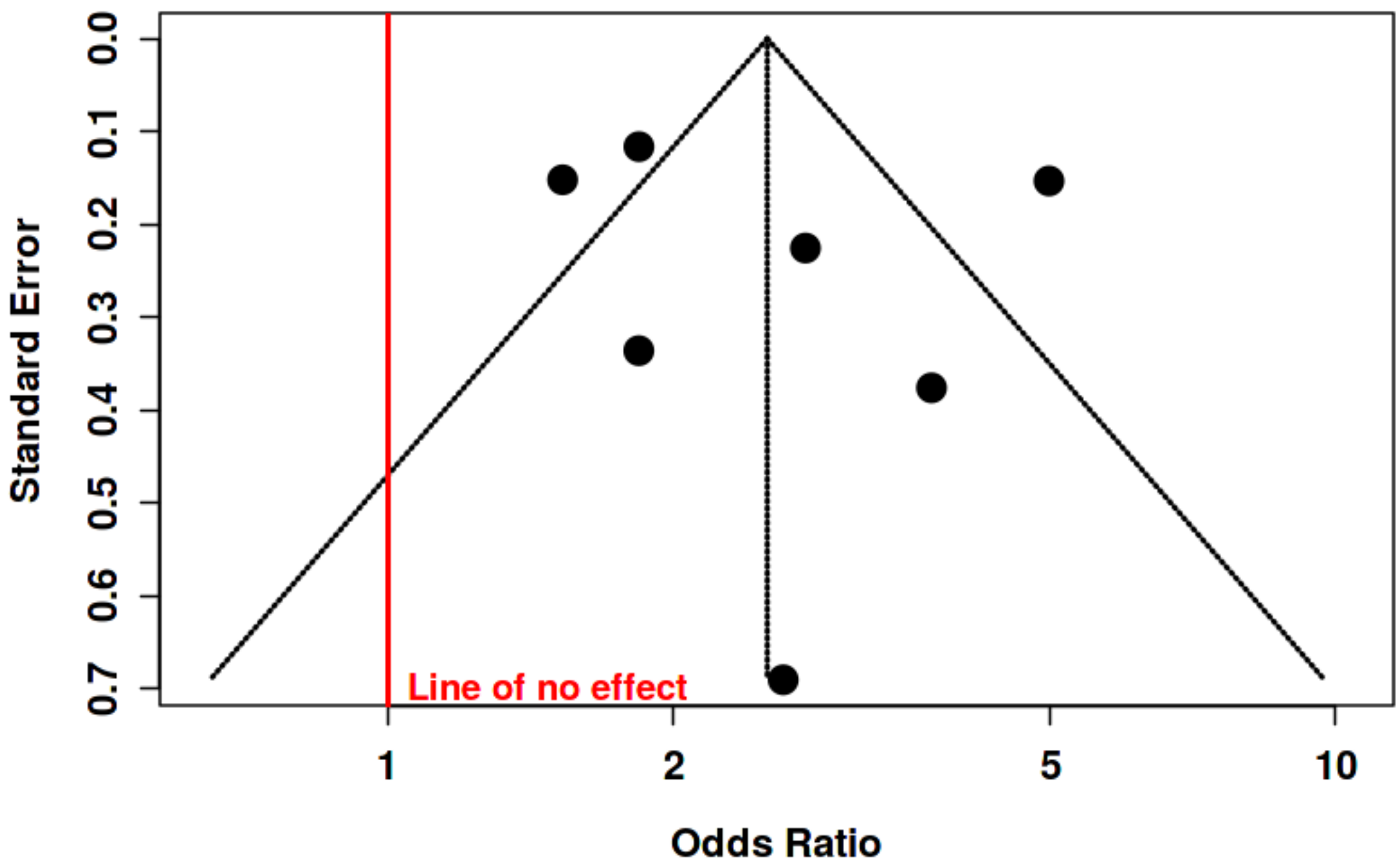

Scalp Wound

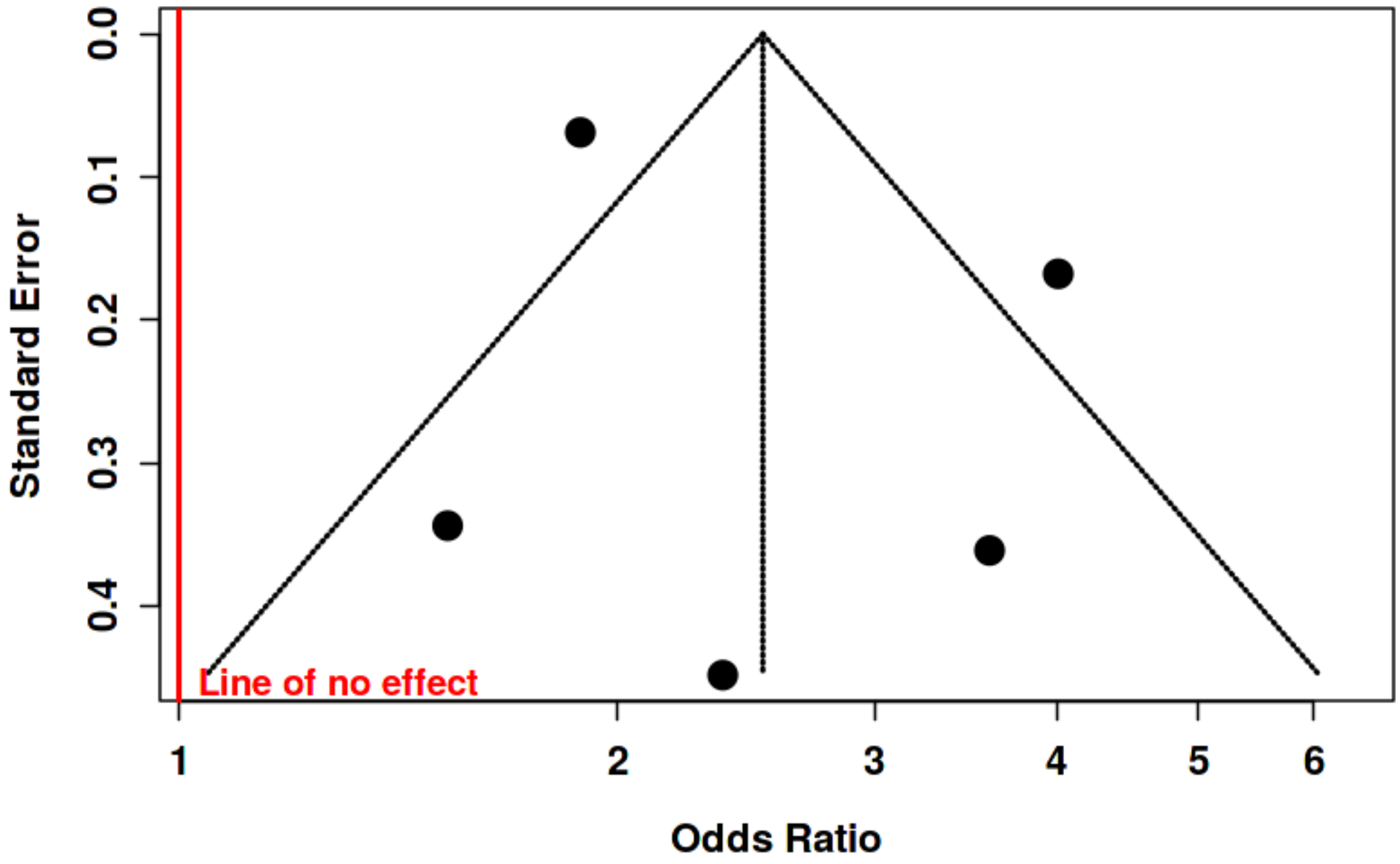

Seizure

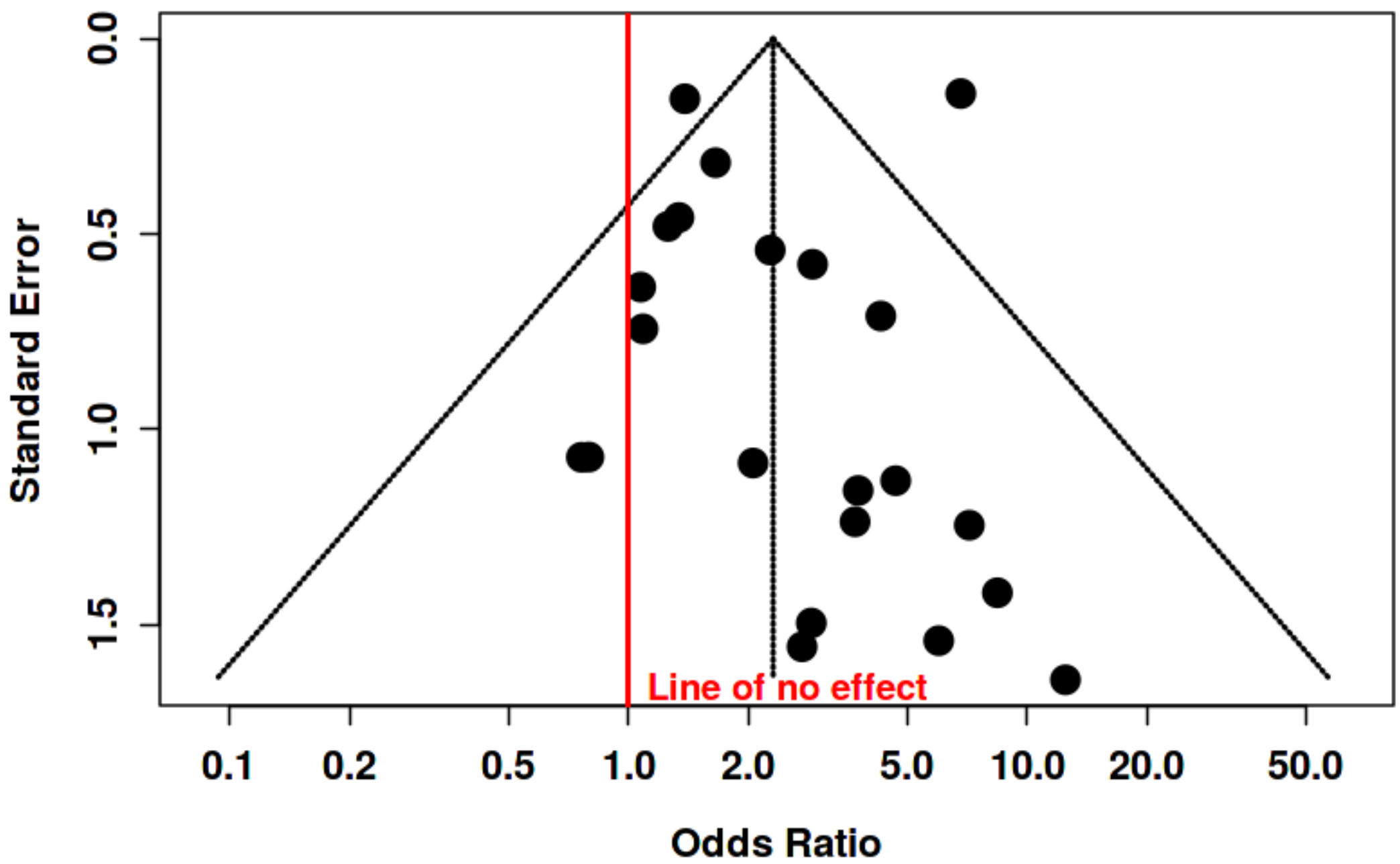

Signs of Injury Above Clavicle

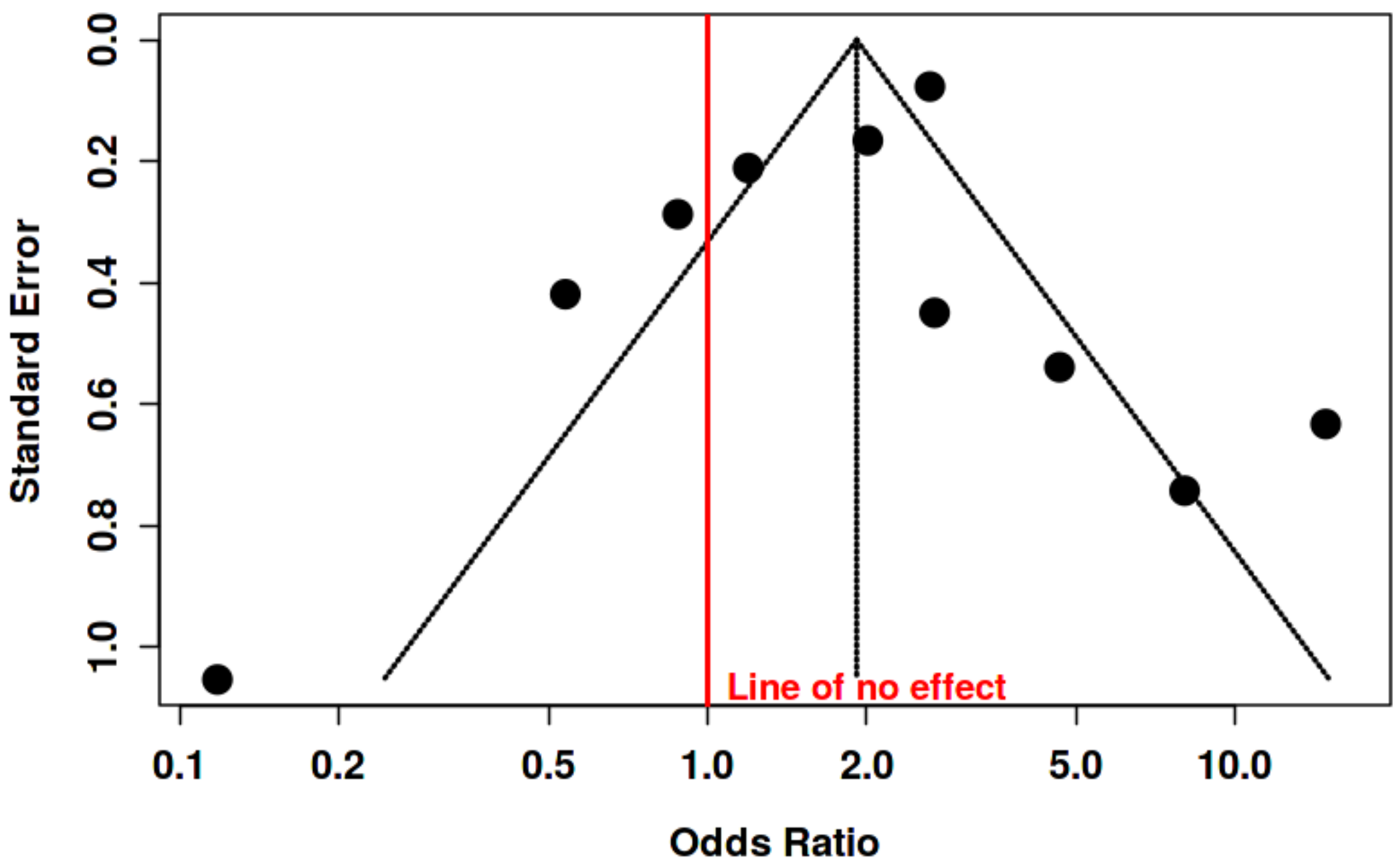

Signs of Skull Fracture

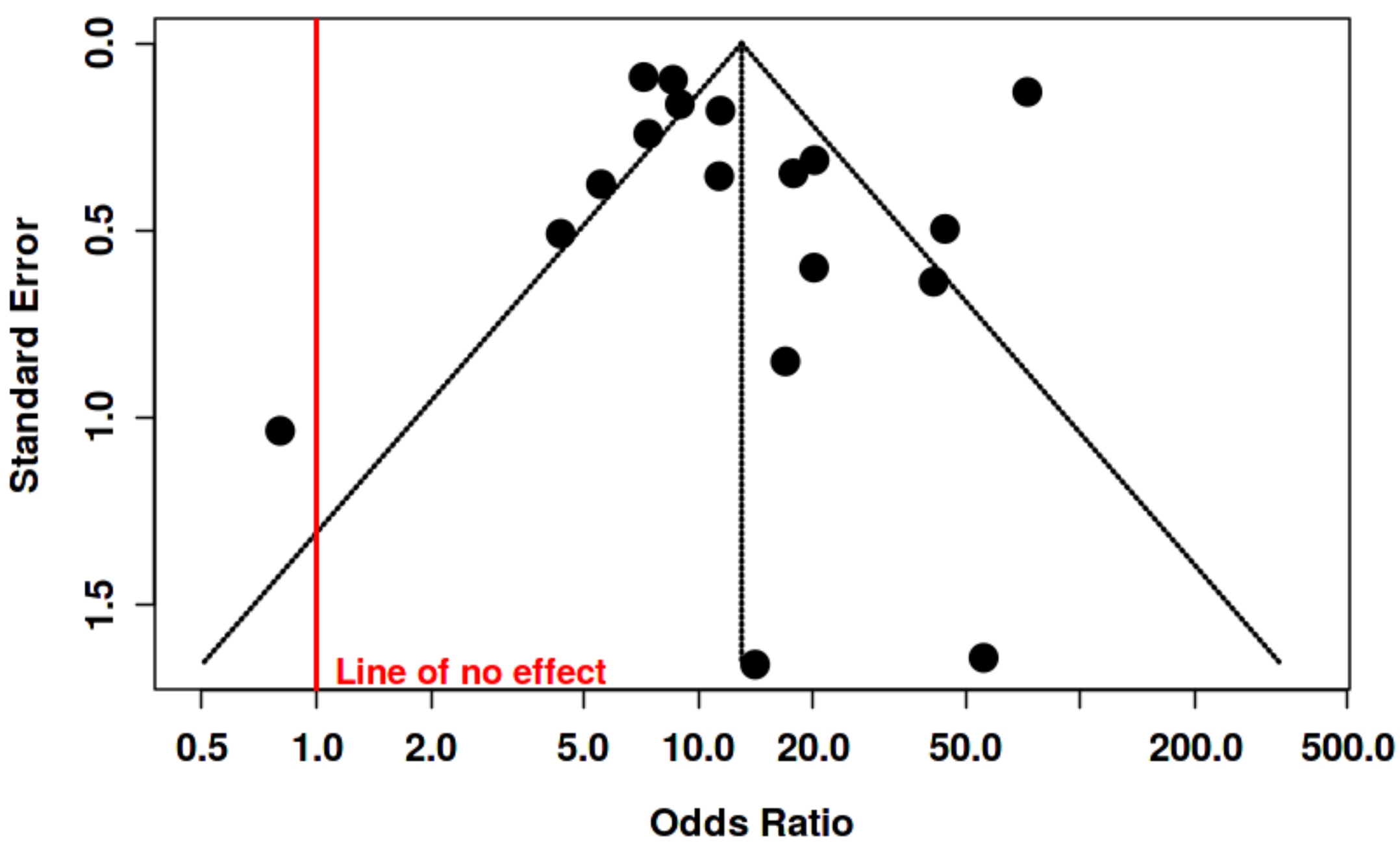

Vitamin K Antagonist

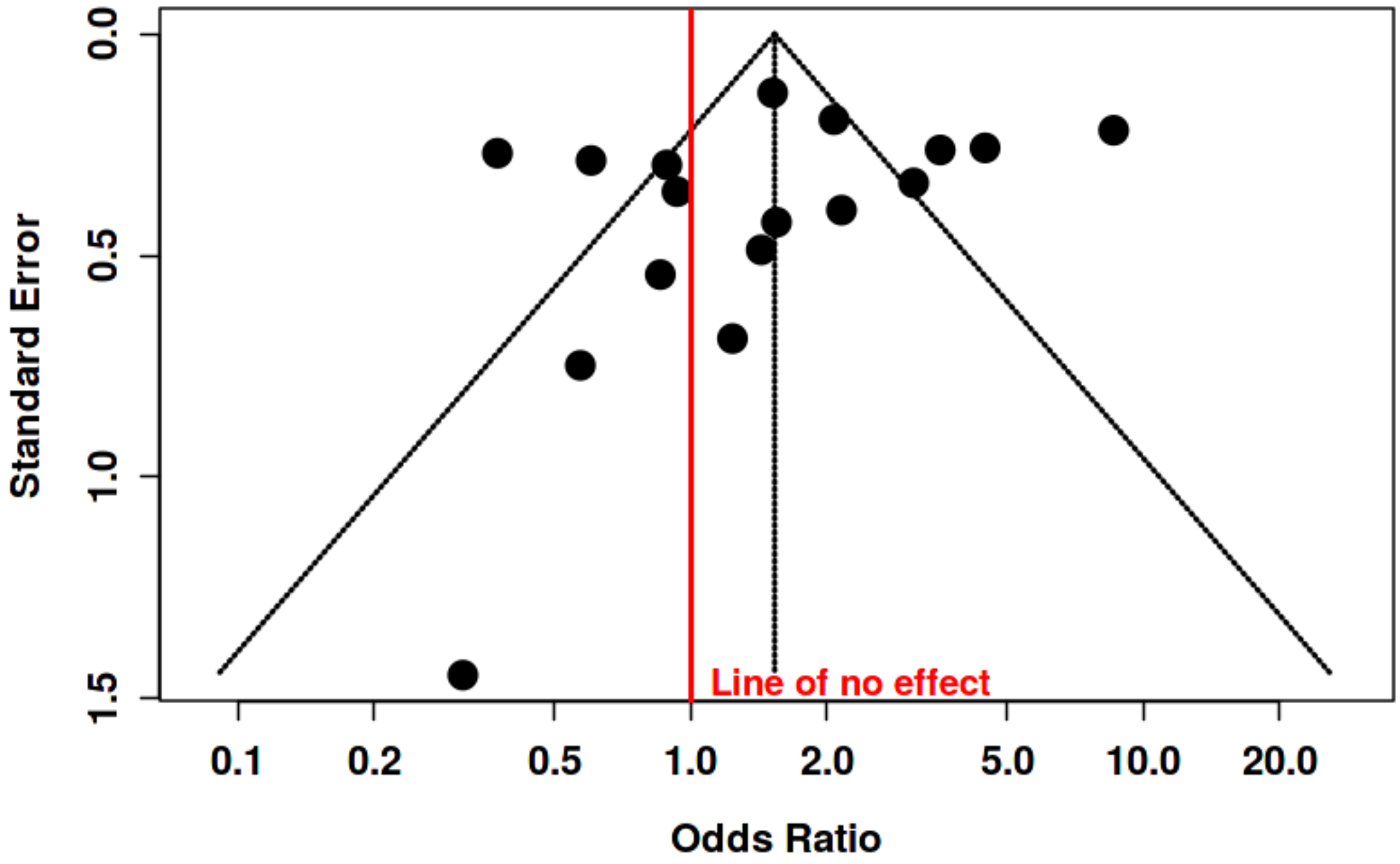

Vomiting Once

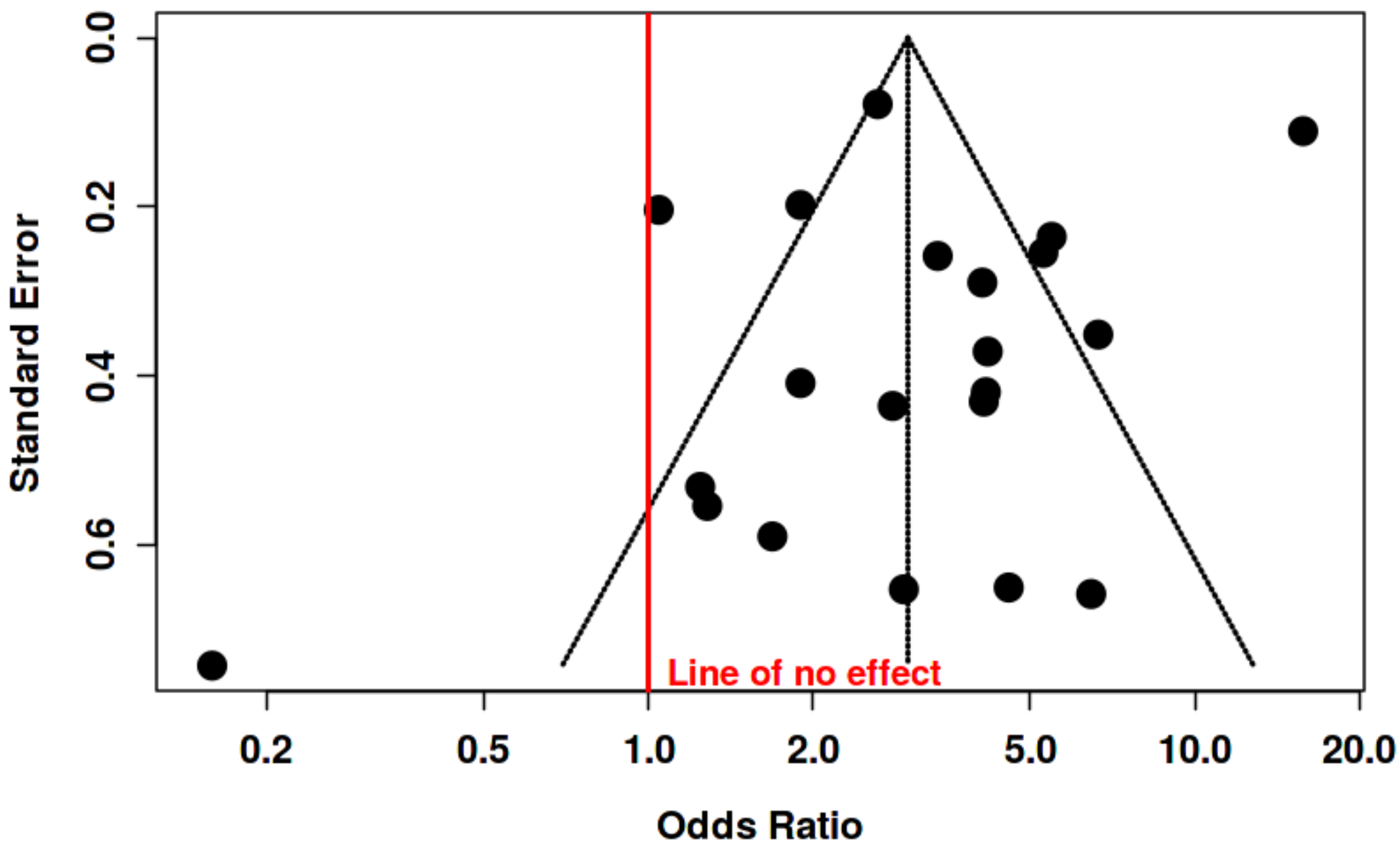

6. Forest Plots (Neurosurgical Intervention)

Anticoagulation

Study	Anticoagulation Events	Anticoagulation Total	No Anticoagulation Events	No Anticoagulation Total	Weight	Risk Ratio IV, Random, 95% CI
Martin 2018	4	23	13	222	11.1%	2.97 [1.05; 8.36]
Ward 2022	3	18	15	216	9.1%	2.40 [0.77; 7.52]
Patel 2023	30	239	96	1303	79.8%	1.70 [1.16; 2.51]
**Total (95% CI)**		**280**		**1741**	**100.0%**	**1.87 [1.32; 2.64]**
**Prediction interval**						**[0.88; 3.99]**

Heterogeneity: $Tau^2 = 0$; $Chi^2 = 1.17$, df = 2 (P = 0.5560); $I^2 = 0.0\%$
Test for overall effect: Z = 3.56 (P = 0.0004)

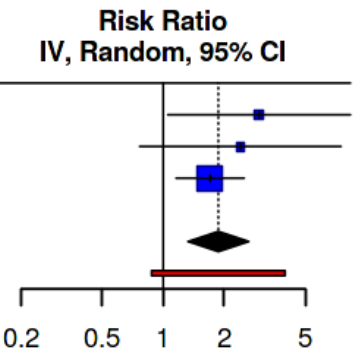

Antiplatelet Therapy

Study	APT Events	APT Total	No APT Events	No APT Total	Weight	Risk Ratio IV, Random, 95% CI
Thorson 2013	5	16	25	344	15.9%	4.30 [1.90; 9.75]
Sumritpradit 2016	5	31	3	67	10.7%	3.60 [0.92; 14.13]
Martin 2018	3	76	13	222	11.8%	0.67 [0.20; 2.30]
Savioli 2021	6	483	6	1443	12.8%	2.99 [0.97; 9.22]
Antoni 2022	6	452	11	717	14.1%	0.87 [0.32; 2.32]
Ward 2022	5	66	13	268	14.1%	1.56 [0.58; 4.23]
Patel 2023	49	678	77	864	20.6%	0.81 [0.58; 1.14]
**Total (95% CI)**		**1802**		**3925**	**100.0%**	**1.59 [0.85; 2.95]**
**Prediction interval**						**[0.26; 9.83]**

Heterogeneity: $Tau^2 = 0.4555$; $Chi^2 = 20.78$, df = 6 (P = 0.0020); $I^2 = 71.1\%$
Test for overall effect: Z = 1.45 (P = 0.1457)

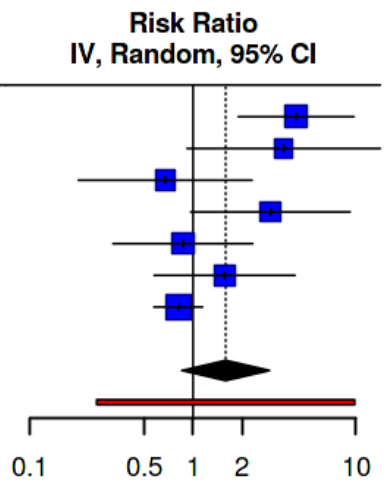

Direct Oral Anticoagulation (DOAC)

Study	DOAC Events	DOAC Total	No DOAC Events	No DOAC Total	Weight	Risk Ratio IV, Random, 95% CI
Antoni 2022	3	456	14	713	32.4%	0.34 [0.10; 1.16]
Della Pepa 2022	1	23	29	253	13.2%	0.38 [0.05; 2.66]
Patel 2023	4	54	122	1488	54.4%	0.90 [0.35; 2.36]
**Total (95% CI)**		**533**		**2454**	**100.0%**	**0.58 [0.29; 1.18]**
**Prediction interval**						**[0.12; 2.76]**

Heterogeneity: $Tau^2 = 0$; $Chi^2 = 1.75$, df = 2 (P = 0.4159); $I^2 = 0.0\%$
Test for overall effect: Z = -1.49 (P = 0.1362)

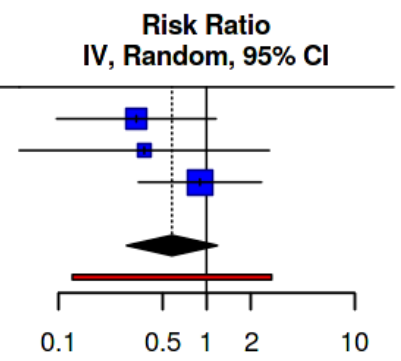

Glasgow Coma Scale Score <15

Study	GCS<15 Events	GCS<15 Total	GCS=15 Events	GCS=15 Total	Weight	Risk Ratio IV, Random, 95% CI
Thorson 2013	17	167	13	193	20.8%	1.51 [0.76; 3.02]
Chojak 2021	3	67	0	119	1.7%	12.39 [0.65; 236.33]
Tourigny 2021	14	176	26	302	23.7%	0.92 [0.50; 1.72]
Karanci 2022	5	45	17	396	13.2%	2.59 [1.00; 6.68]
Patel 2023	45	368	81	1174	40.6%	1.77 [1.26; 2.50]
**Total (95% CI)**		**823**		**2184**	**100.0%**	**1.60 [1.08; 2.36]**
**Prediction interval**						**[0.64; 3.96]**

Heterogeneity: $Tau^2 = 0.0671$; $Chi^2 = 6.19$, df = 4 (P = 0.1854); $I^2 = 35.4\%$
Test for overall effect: Z = 2.35 (P = 0.0190)

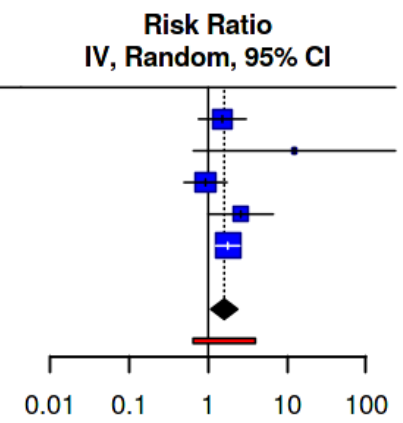

Male Sex

Study	Male Sex Events	Male Sex Total	Female Sex Events	Female Sex Total	Weight	Risk Ratio IV, Random, 95% CI
Thorson 2013	22	263	8	97	15.9%	1.01 [0.47; 2.20]
Sumritpradit 2016	6	61	2	37	4.5%	1.82 [0.39; 8.55]
Tourigny 2021	28	327	12	151	21.3%	1.08 [0.56; 2.06]
Ward 2022	12	147	6	69	11.4%	0.94 [0.37; 2.40]
Patel 2023	87	826	39	716	46.9%	1.93 [1.34; 2.78]
**Total (95% CI)**		**1624**		**1070**	**100.0%**	**1.41 [1.01; 1.98]**
**Prediction interval**						**[0.73; 2.75]**

Heterogeneity: $Tau^2 = 0.0280$; $Chi^2 = 4.87$, df = 4 (P = 0.3011); $I^2 = 17.8\%$
Test for overall effect: Z = 2.03 (P = 0.0428)

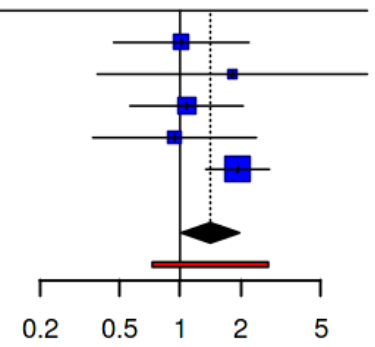

Vitamin K Antagonist

Study	VKA Events	VKA Total	No VKA Events	No VKA Total	Weight	Risk Ratio IV, Random, 95% CI
Thorson 2013	4	9	26	351	26.0%	6.00 [2.65; 13.61]
Sumritpradit 2016	0	4	8	94	7.4%	1.24 [0.08; 18.21]
Antoni 2022	2	229	15	940	16.5%	0.55 [0.13; 2.38]
Della Pepa 2022	2	23	29	253	17.8%	0.76 [0.19; 2.98]
Patel 2023	26	185	100	1357	32.3%	1.91 [1.27; 2.85]
**Total (95% CI)**		**450**		**2995**	**100.0%**	**1.72 [0.76; 3.91]**
**Prediction interval**						**[0.18; 16.87]**

Heterogeneity: $Tau^2 = 0.5012$; $Chi^2 = 12.00$, df = 4 (P = 0.0173); $I^2 = 66.7\%$
Test for overall effect: Z = 1.29 (P = 0.1965)

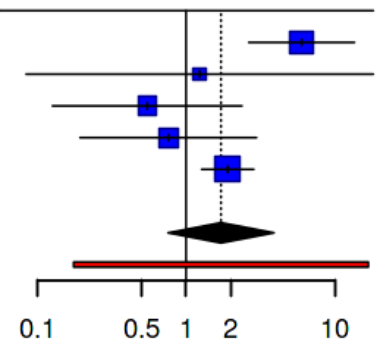

7. Sensitivity Analysis (Neurosurgical Intervention)

Anticoagulation

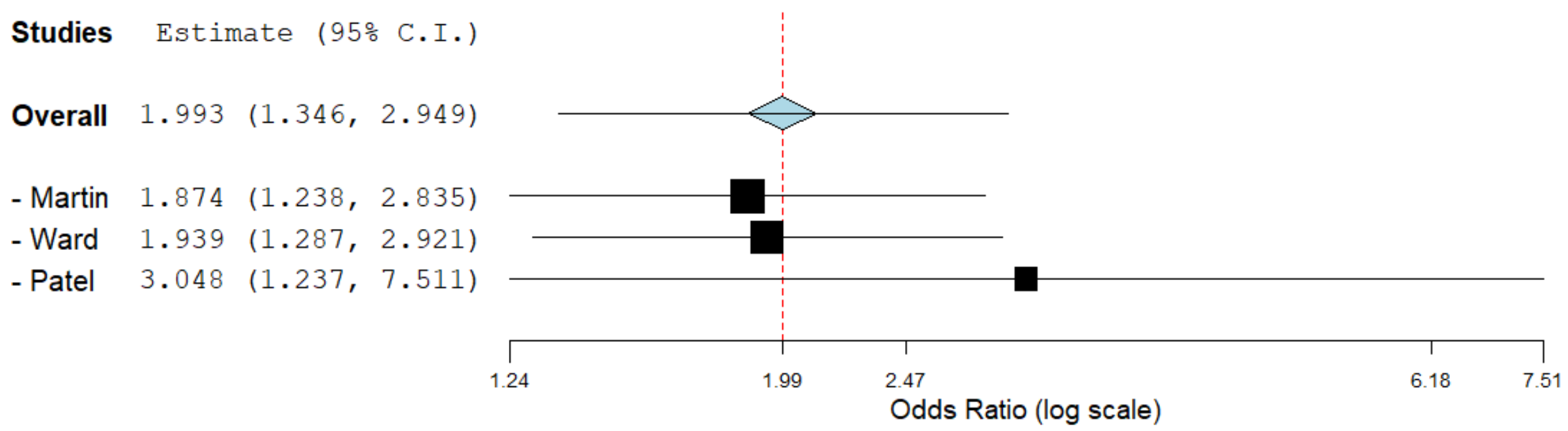

Antiplatelet Therapy

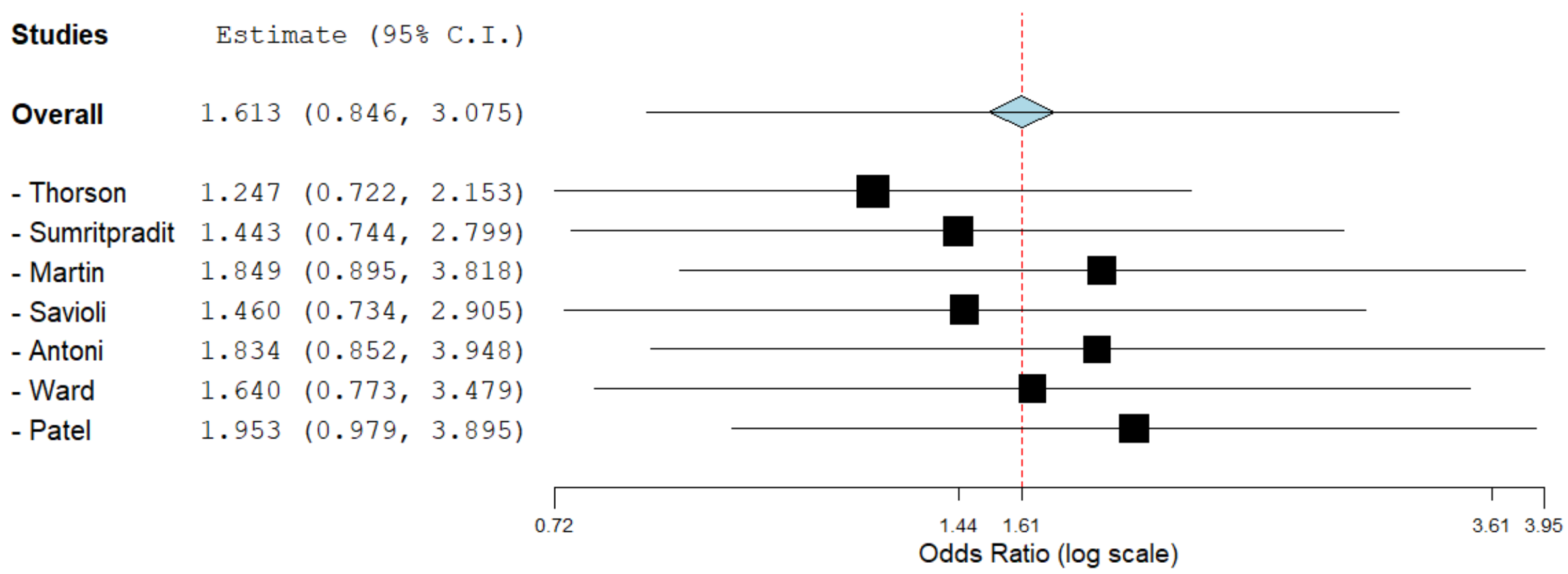

Direct Oral Anticoagulation (DOAC)

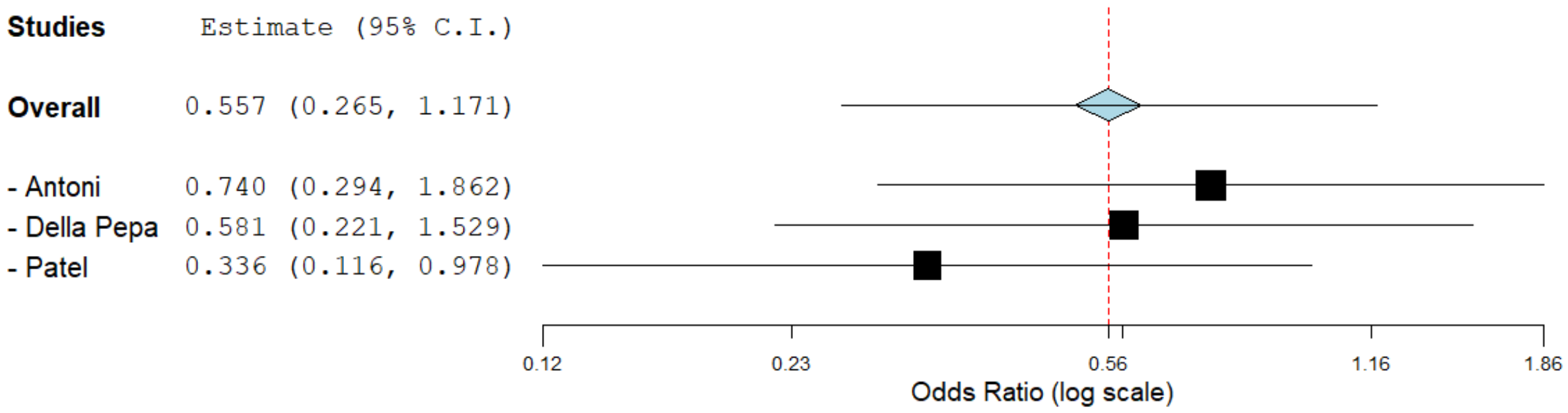

Glasgow Coma Scale Score <15

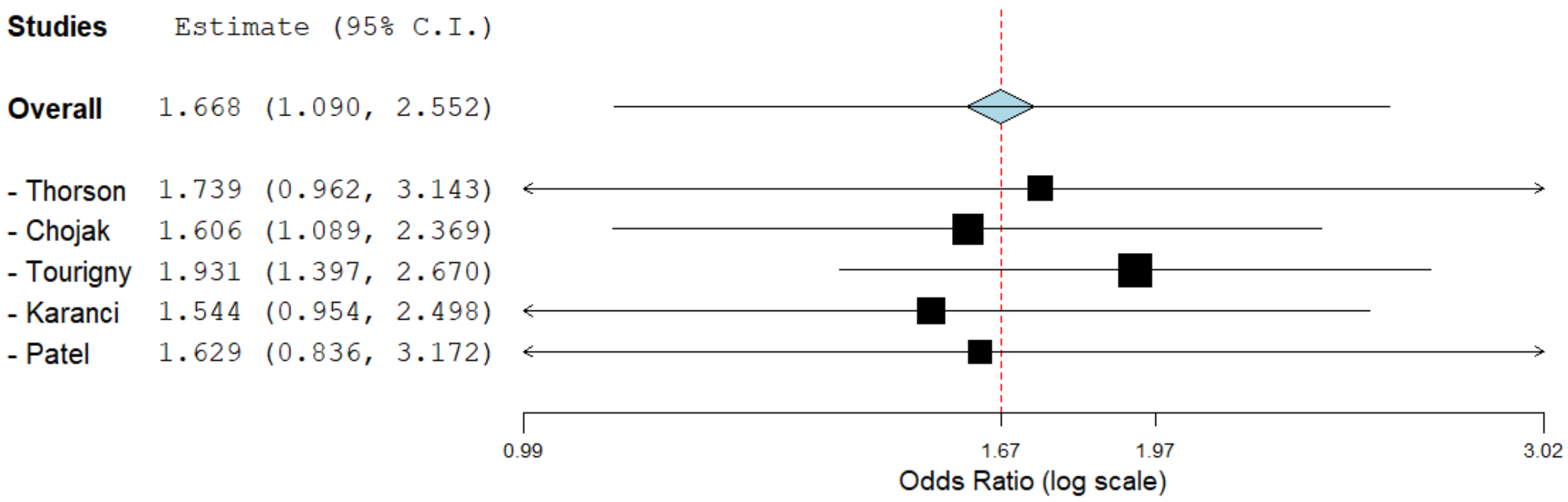

Male Sex

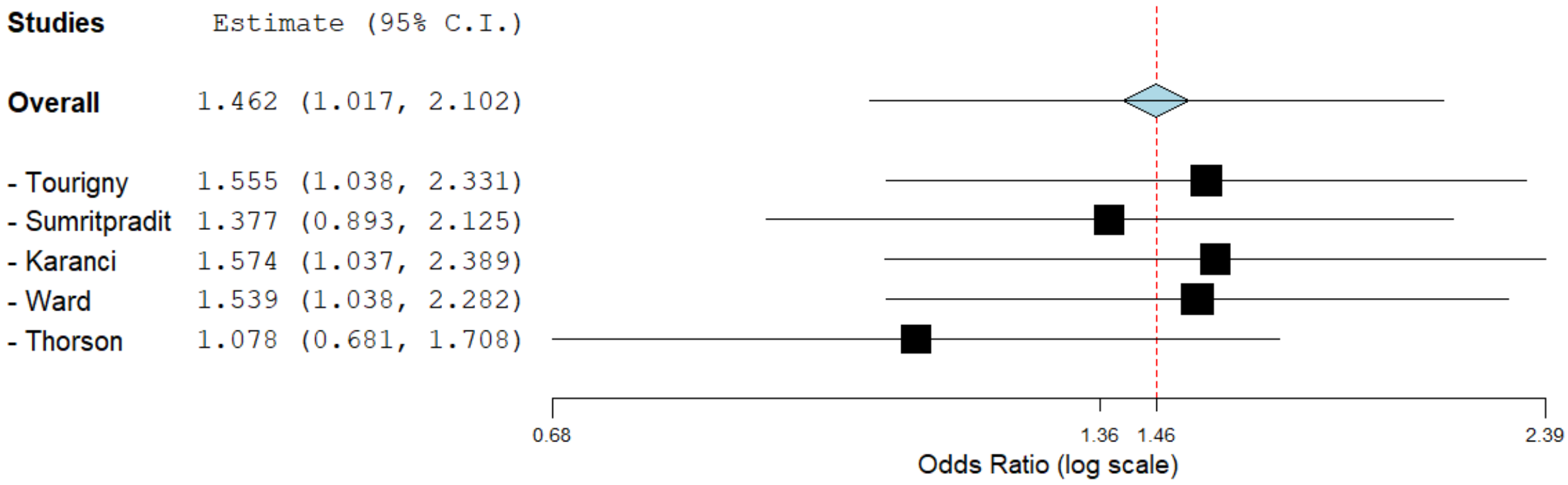

Vitamin K Antagonist

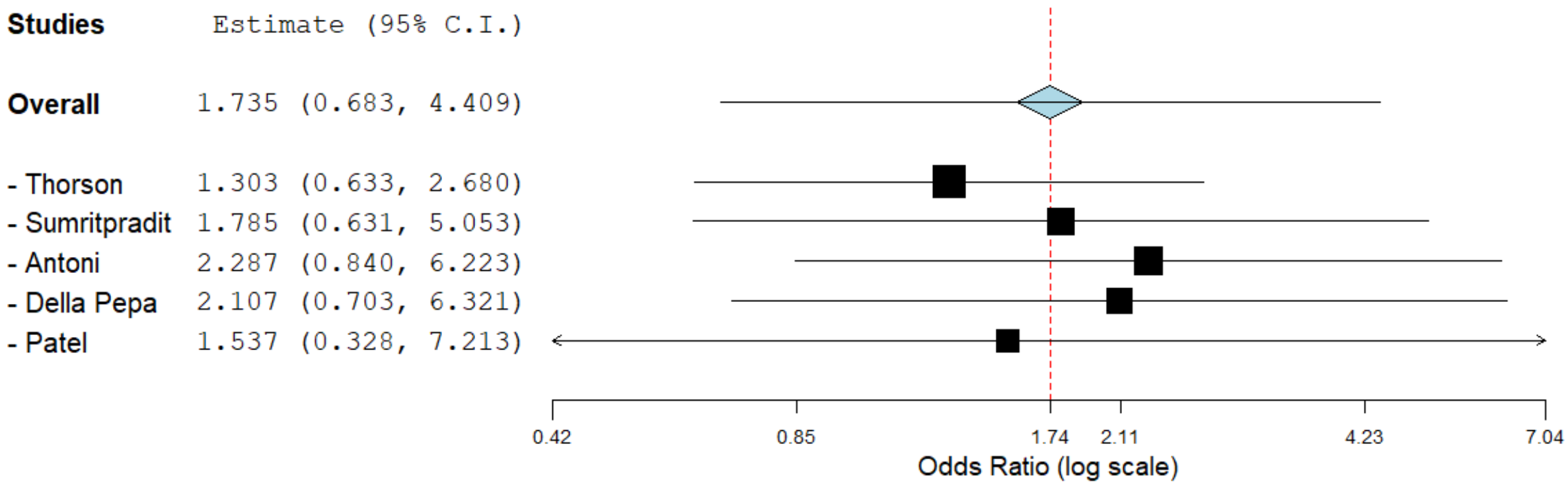

8. Funnel Plots (Neurosurgical Intervention)

Anticoagulation

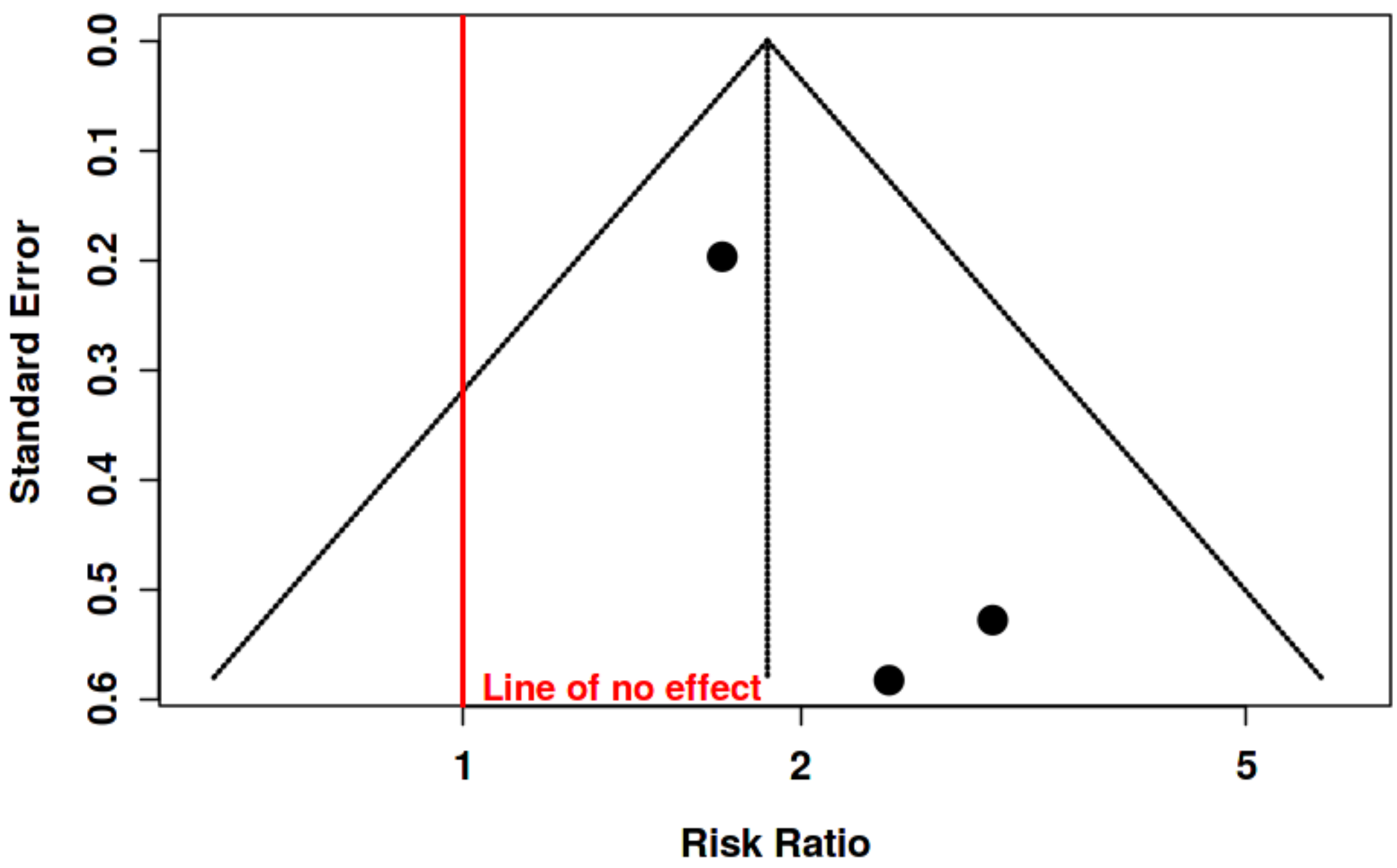

Antiplatelet Therapy

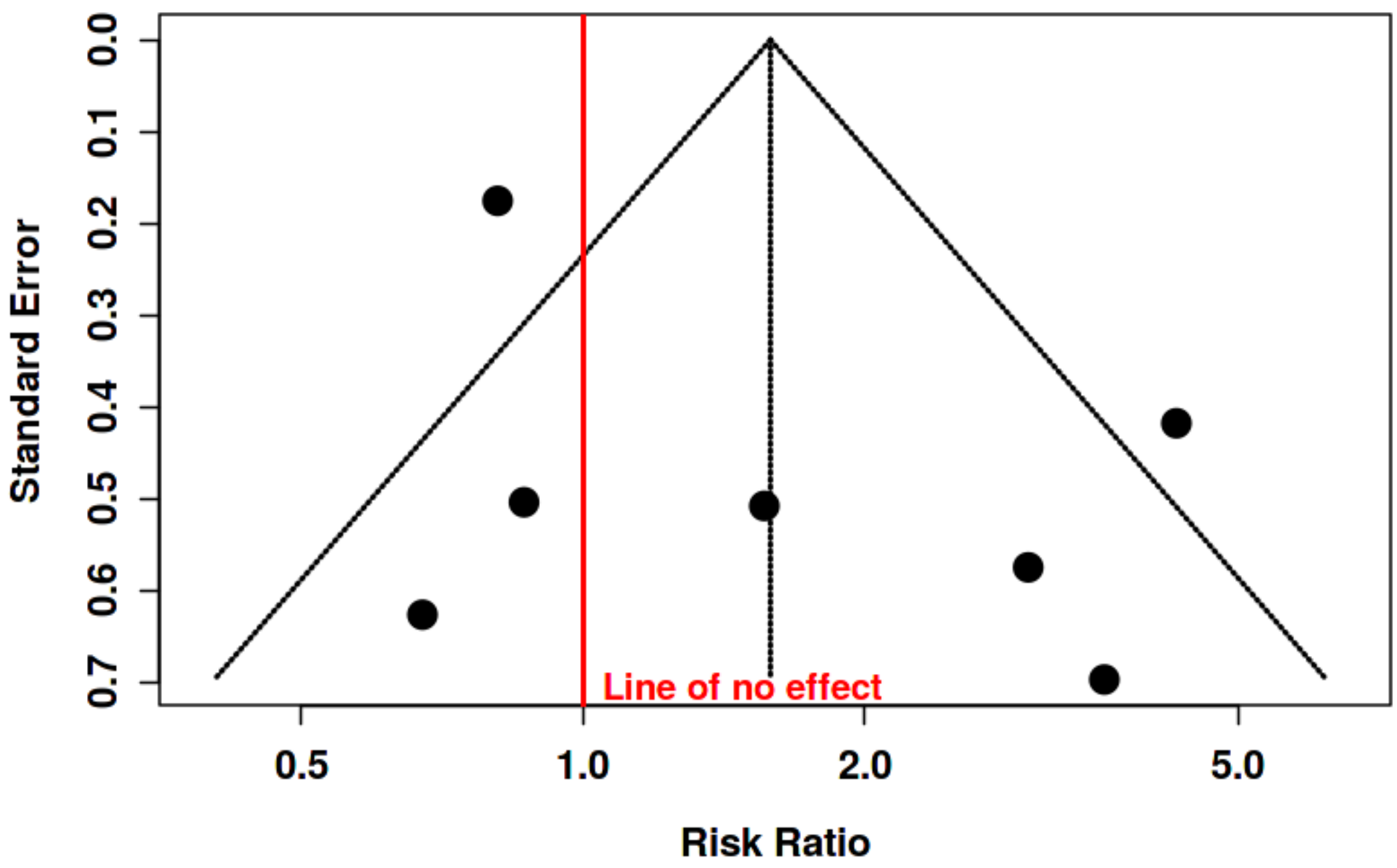

Direct Oral Anticoagulation (DOAC)

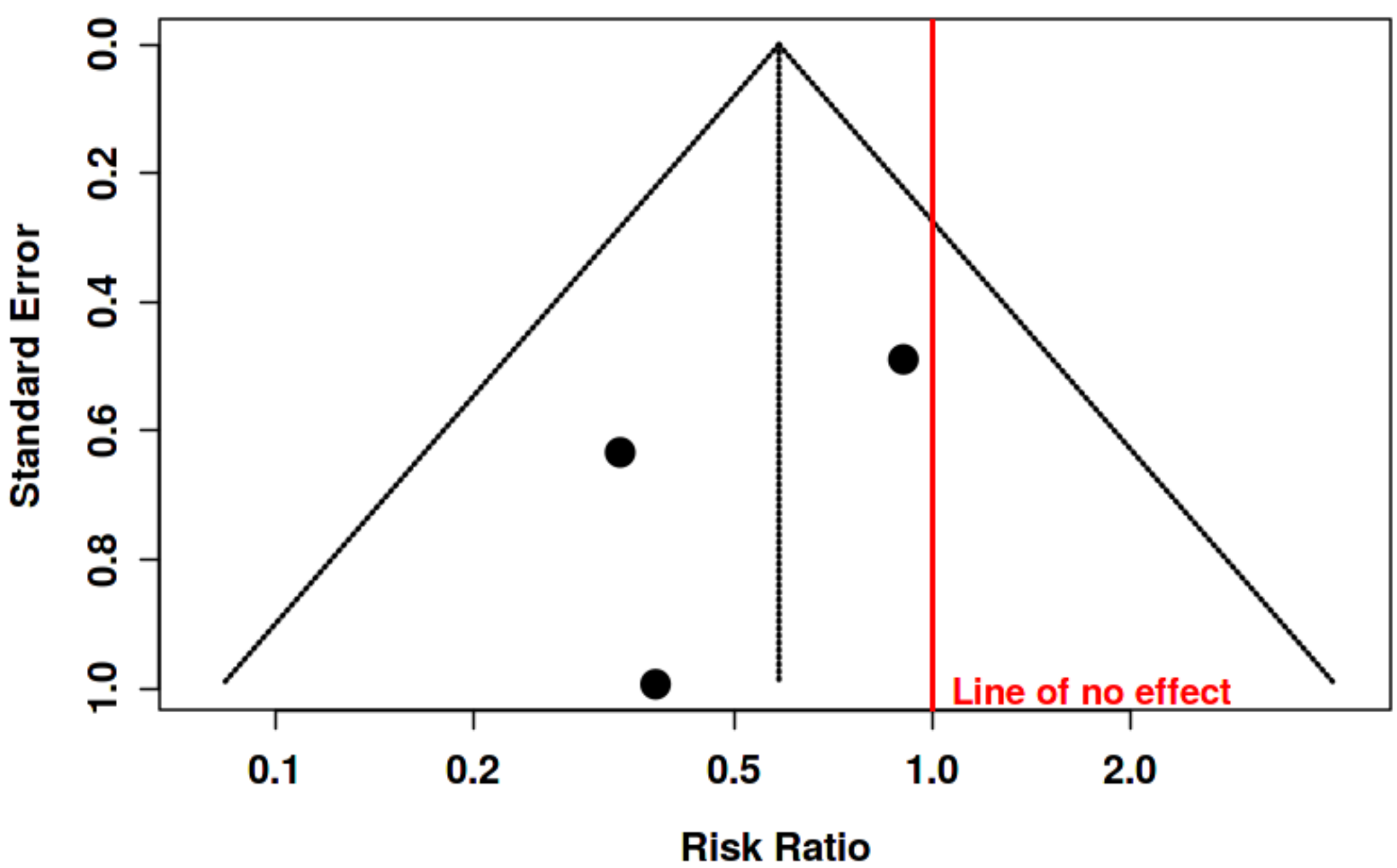

Glasgow Coma Scale Score <15

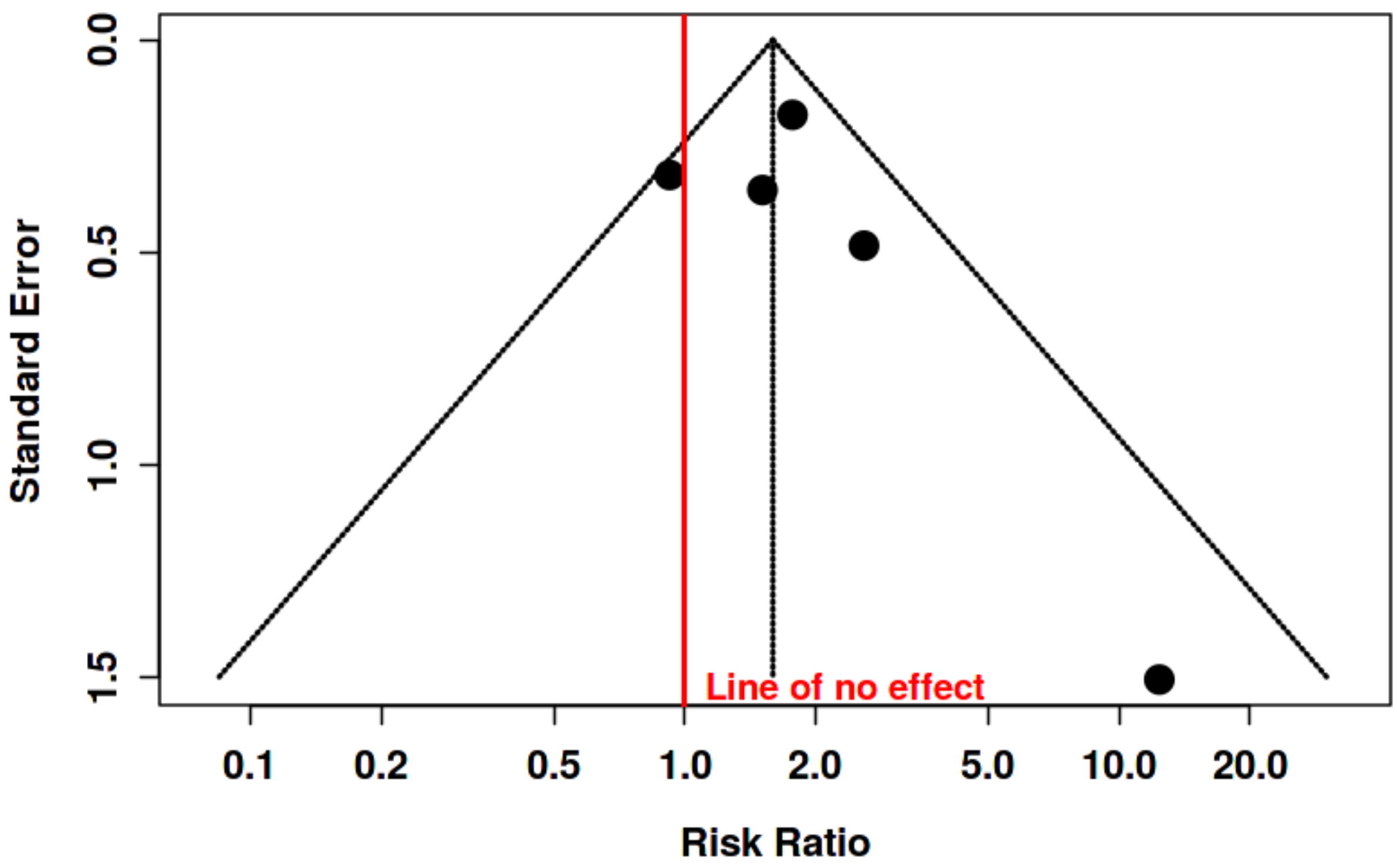

Male Sex

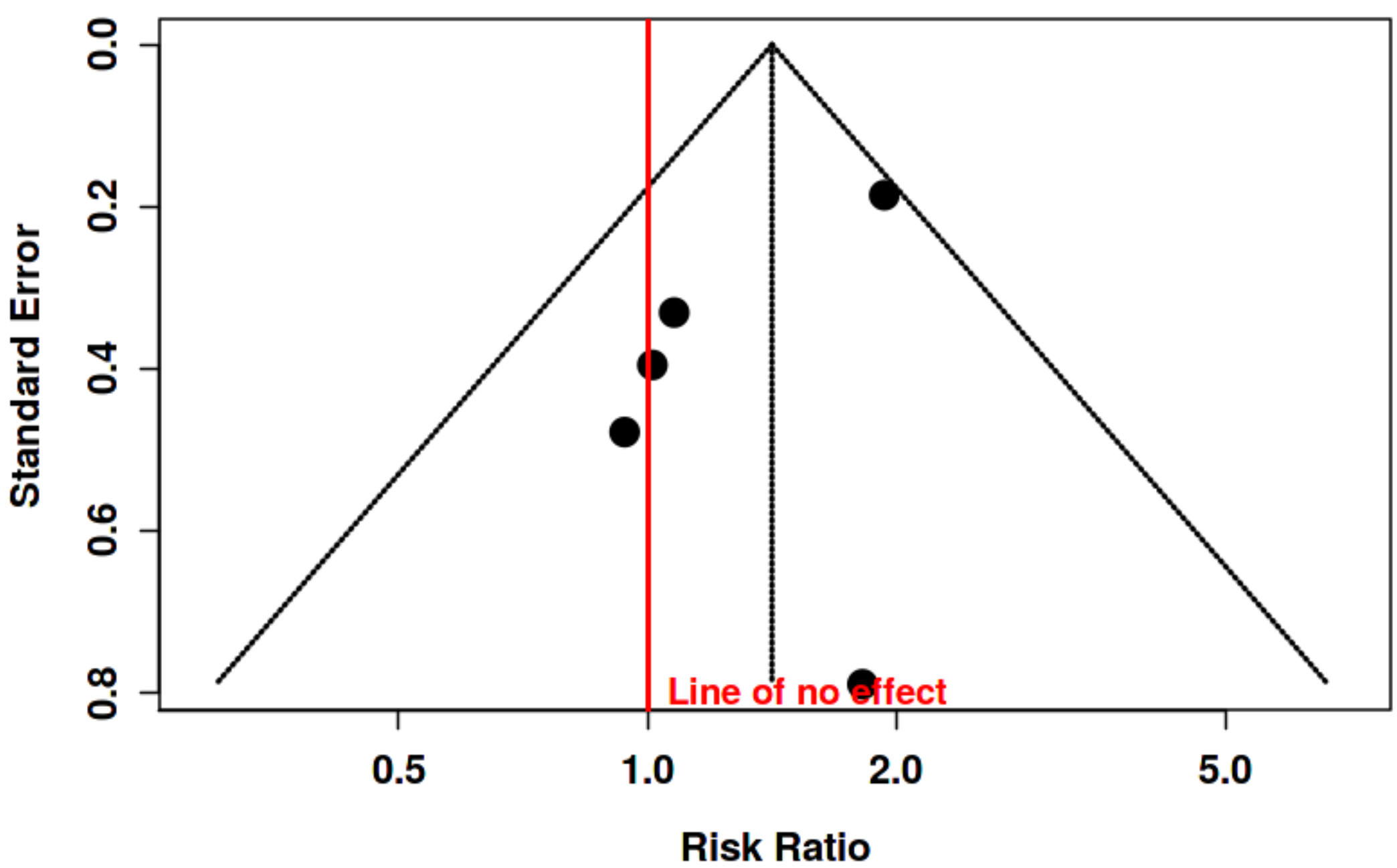

Vitamin K Antagonist

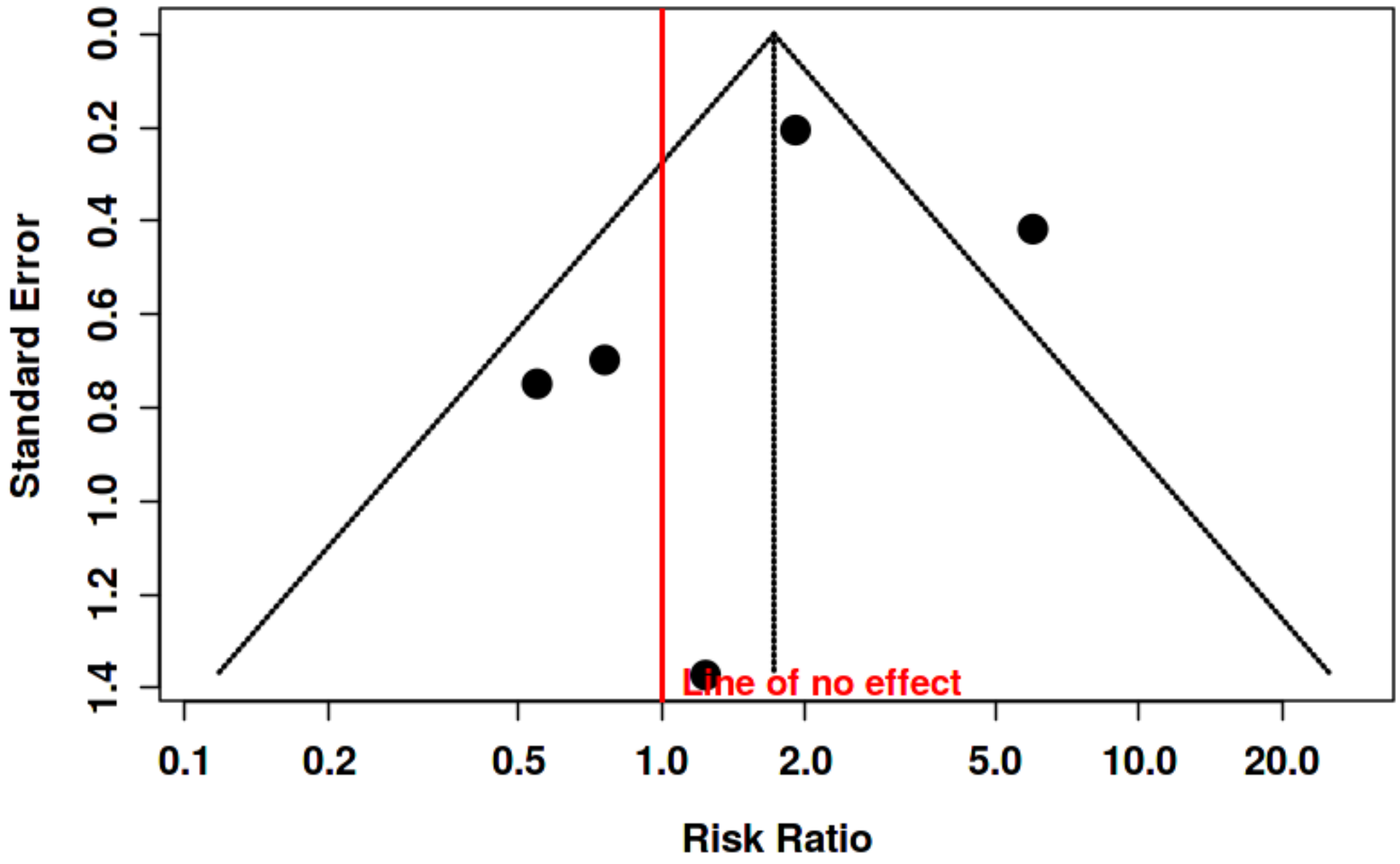

9. Quality Assessment of Diagnostic Accuracy Studies (QUADAS) Summary (Traumatic Intracranial Injury)

Overall Summary

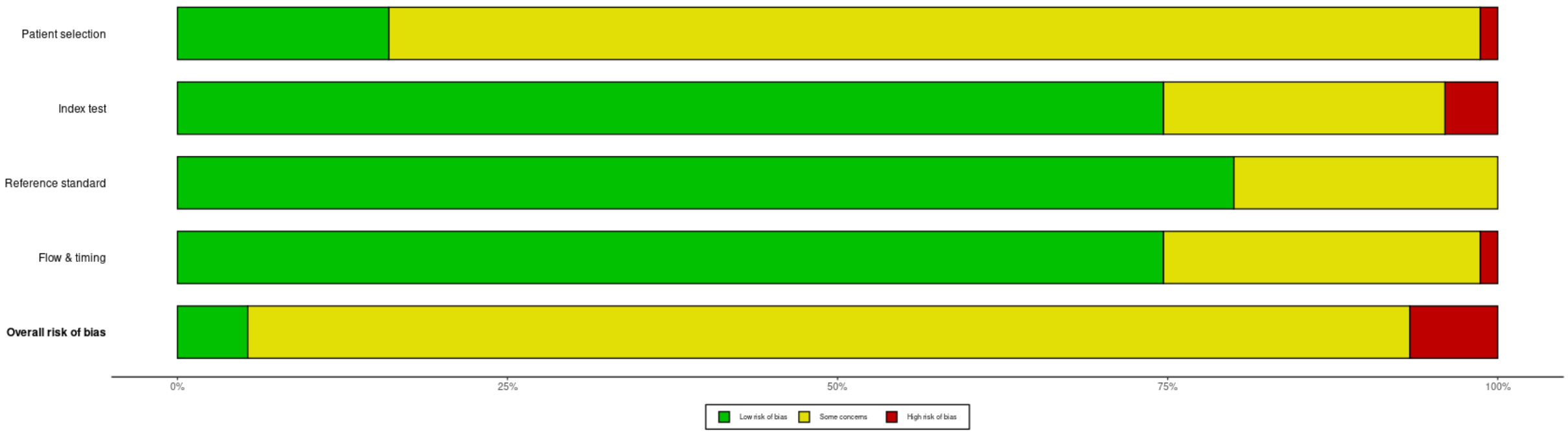

Studies from the 2013 Data Synthesis

Risk of bias domains

Study	D1	D2	D3	D4	Overall
Abdul Latip	-	+	+	+	-
Abdul Rahman	-	+	+	-	-
Barrow	-	-	+	-	-
Bazarian	-	+	+	X	X
Biberthaler	+	-	-	+	-
Biberthaler 2	-	+	+	-	-
Biberthaler 3	-	+	+	-	-
Bouvier	-	+	+	-	-
Brewer	-	+	+	-	-
Claudia	+	+	+	+	+
Deboussard	-	+	+	-	-
Fabbri	-	-	+	+	-
Fabbri 2	X	+	+	-	X
Fabbri 3	+	-	+	-	-
Falimirski	-	+	+	+	-
Haydel	-	+	+	+	-
Ibanez	-	+	+	+	-
Ingebrigtsen	+	-	-	-	-
Jacobs	+	-	+	-	-
Kisat	-	+	+	-	-
Kotlyar	-	+	+	-	-
Livingston	-	+	+	+	-
Mack	-	+	-	+	-
Moore	+	+	+	+	+
Morochovic	-	+	+	-	-
Mower	-	+	+	+	-
Muller	-	-	-	+	-
Muller 2	-	+	+	-	-
Munoz-Sanchez	-	+	+	+	-
Mussack	-	X	+	+	X
Poli-de-Figueiredo	+	-	-	+	-
Saboori	+	+	+	-	-
Smits	-	+	+	-	-
Stiell	-	-	+	+	-
Stiell 2	-	-	+	+	-
Turedi	-	+	+	-	-
Zongo	+	+	+	+	+

Domains:
D1: Patient selection.
D2: Index test.
D3: Reference standard.
D4: Flow & timing.

Judgement
X High
- Some concerns
+ Low

Studies from the 2024 Data Synthesis

Risk of bias domains

Study	D1	D2	D3	D4	Overall
Allouchery	-	-	+	+	-
Alrajhi	-	+	+	+	-
Asadollahi	-	+	-	+	-
Bazarian	-	+	-	+	-
Calcagnile	-	-	+	+	-
Chayoua	-	+	-	+	-
Cipriano	-	+	-	+	-
Colas	-	+	+	+	-
Dupuis	-	+	+	+	-
Foks	-	-	+	+	-
Galliazzo	+	-	+	+	-
Gill	-	+	+	+	-
Hofmann	-	+	+	+	-
Hosseininejad	-	+	+	+	-
Innocenti	-	+	+	+	-
Jame	-	+	+	+	-
Kahouadji	-	+	+	+	-
Lagerstedt	-	+	+	+	-
Leitner	-	-	-	+	-
Levine	-	+	+	+	-
Martínez-Rivas	-	+	+	+	-
Mishra	-	+	+	+	-
Molaei-Langroudi	-	+	+	+	-
Niklasson	+	+	+	+	+
Nishijima	-	+	+	+	-
Oris	-	X	+	+	X
Sakkas	-	+	+	+	-
Savioli	-	+	+	+	-
Schindler	-	+	+	+	-
Shafie	-	+	-	+	-
Singh	-	+	+	+	-
Sugimoto	-	+	+	+	-
Teeratakulpisarn	-	+	+	+	-
Turcato	-	+	+	+	-
Vaniyapong	-	-	-	+	-
Yamada	-	X	-	+	X
Yuksen	-	+	-	+	-
Zhang	+	+	-	+	-

Domains:
D1: Patient selection.
D2: Index test.
D3: Reference standard.
D4: Flow & timing.

Judgement
X High
- Some concerns
+ Low

10. Quality Assessment of Diagnostic Accuracy Studies (QUADAS) Summary (Neurosurgical Intervention)

Overall Summary

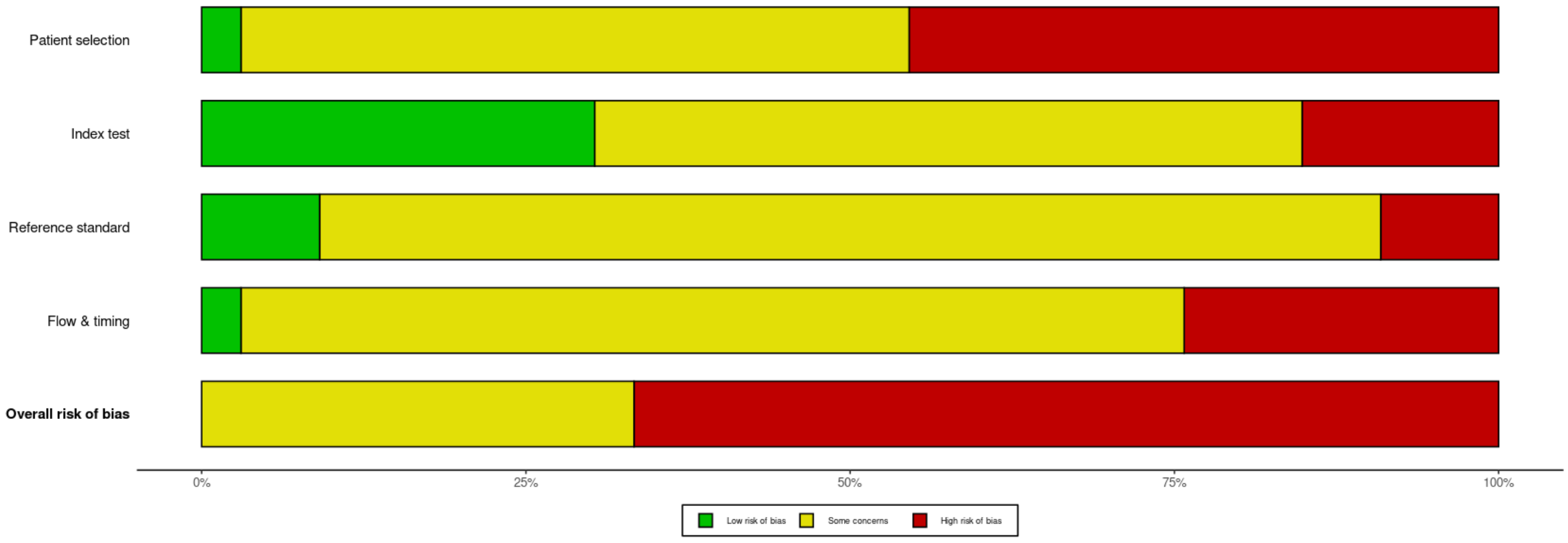

Studies from the 2013 Data Synthesis

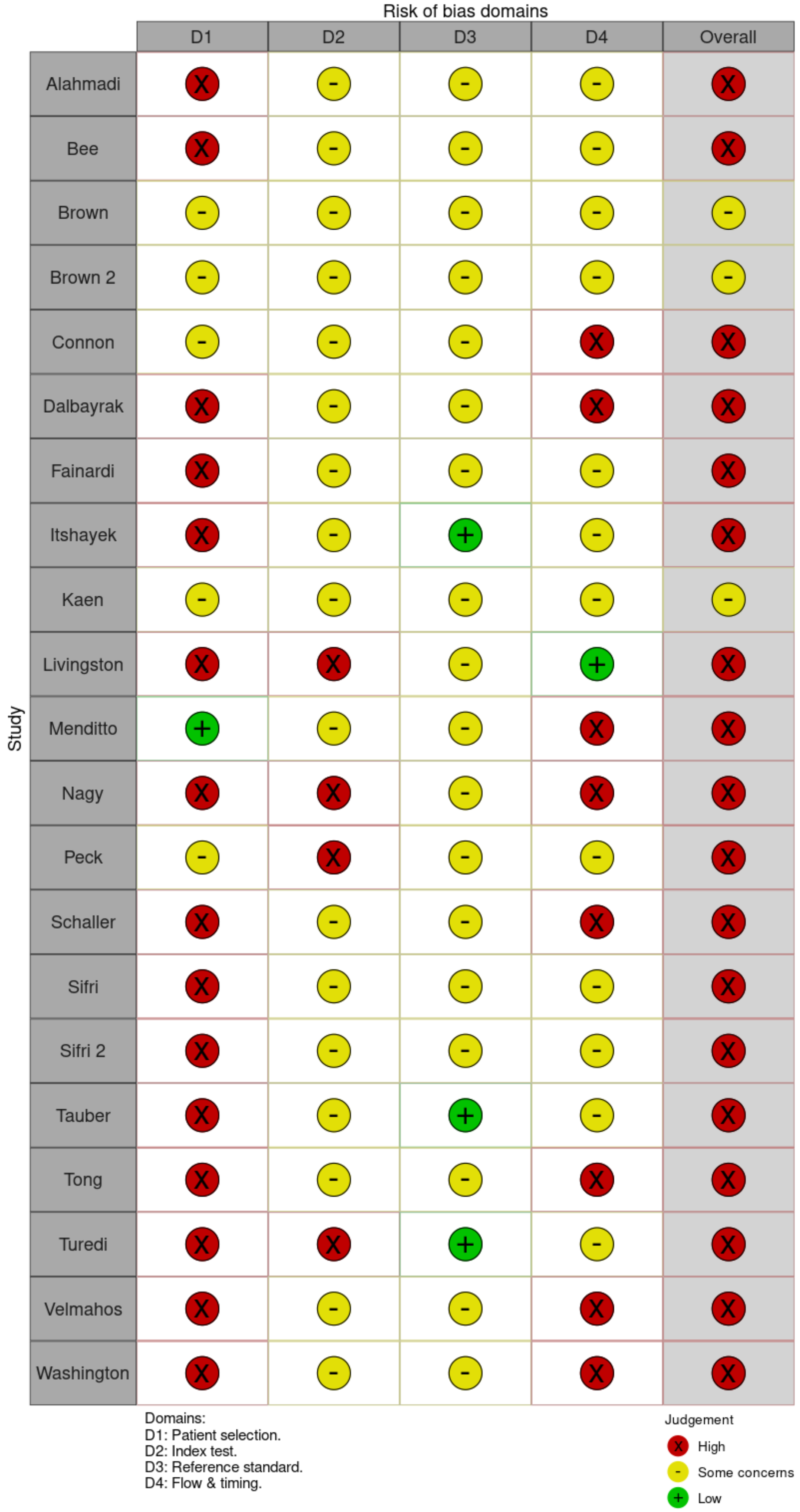

Studies from the 2024 Data Synthesis

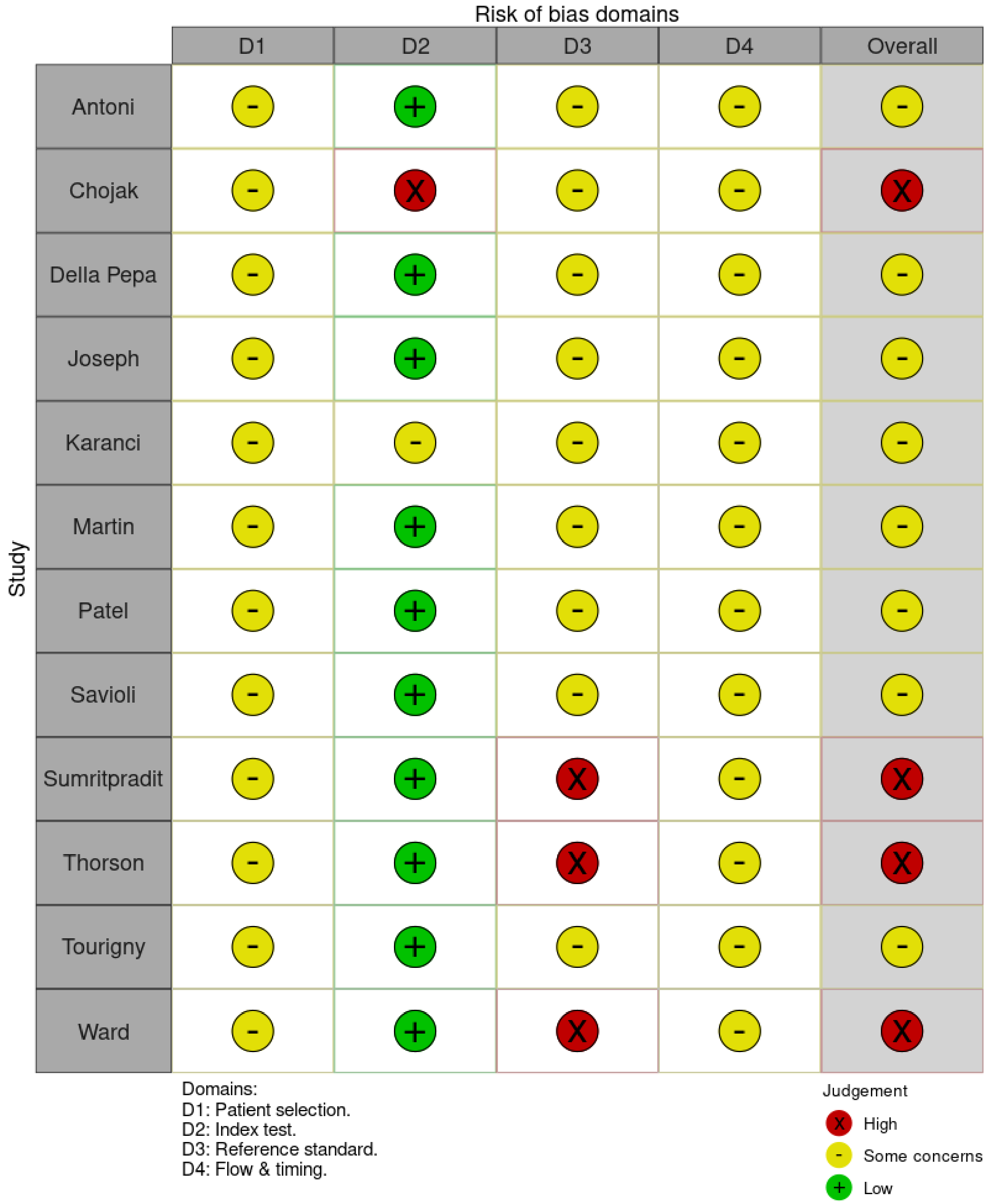

11. Summary of Evidence Grading (Outcomes)

No of studies	Design	Risk of bias	Inconsistency	Indirectness[1]	Imprecision	Other[2]	Certainty (overall score)[3]
**Outcome: Traumatic intracranial injury**							
110	36 retrospective observational<br>74 prospective observational	-1	0	0	0	0	⊕⊕⊕◯ **Moderate**
**Outcome: Neurosurgical intervention**							
33	23 retrospective observational<br>10 prospective observational	-1	-1	0	0	0	⊕⊕◯◯ **Low**

Initial level of evidence is moderate for both outcomes due to the availability of solely observational studies. Due to availability of a large quantity of data, the level of evidence for both outcomes are upgraded to high. Due to significant risk of selection bias based on bias assessment through QUADAS (see appendix), the level of evidence for both outcomes is downgraded to moderate. Finally, due to overall inconsistency across studies in methodology, and outcome definitions, the outcome of neurosurgical intervention is downgraded to low. Further assessment for evidence grading is performed for each individual risk factor.

---

[1] Indirectness includes consideration of

- Indirect (between study) comparisons
- Indirect (surrogate) outcomes
- Applicability (study populations, interventions or comparisons that are different than those of interest)

[2] Other considerations for downgrading include publication bias. Other considerations for upgrading include a strong association with no plausible confounders, a dose response relationship, and if all plausible confounders or biases would decrease the size of the effect (if there is evidence of an effect), or increase it if there is evidence of no harmful effect (safety)

[3] ⊕⊕⊕⊕ **High** = This research provides a very good indication of the likely effect. The likelihood that the effect will be substantially different** is low.

⊕⊕⊕◯ **Moderate** = This research provides a good indication of the likely effect. The likelihood that the effect will be substantially different** is moderate.

⊕⊕◯◯ **Low** = This research provides some indication of the likely effect. However, the likelihood that it will be substantially different** is high.

⊕◯◯◯ **Very low** = This research does not provide a reliable indication of the likely effect. The likelihood that the effect will be substantially different** is very high.

** Substantially different = a large enough difference that it might affect a decision

12. Summary of Evidence Grading (Individual Risk Factors)

Risk factors for traumatic intracranial injury in emergency department head injury patients

Patients: Adult mild head injury or mTBI patients
Setting: Emergency department
Exposure: Risk factor for intracranial injury
Comparison: Control group without presence of the risk factor
Outcome: Head CT verified traumatic intracranial injury

Risk factors	Frequency of outcome		Odds ratio (95% CI)	Number of studies	Additional information	Certainty of the evidence (GRADE)	Rationale
	With risk factor	Without risk factor					
**Age Over Threshold**	8 633 per 25 539	19 929 per 100 979	OR 1.853 (1.470 to 2.334)	26	I^2=92.6% (-1)	⊕⊕⊖⊖ Low	Heterogenous age thresholds across studies. Observational studies, high heterogeneity.
**Anticoagulation**	608 per 2 596	1 480 per 13 103	OR 1.756 (1.068 to 2.889)	13	I^2=88.3% (-1)	⊕⊕⊖⊖ Low	Heterogenous definition of anticoagulation (VKA/DOAC) and comparators. Observational studies, high heterogeneity.
**Antiplatelet Therapy**	813 per 5 757	2 124 per 26 987	OR 1.963 (1.563 to 2.466)	18	I^2=70.3% (-1)	⊕⊕⊖⊖ Low	Heterogenous comparators. Observational studies, medium heterogeneity.
**Dual Antiplatelet Therapy**	28 per 118	1 053 per 6 740	OR 2.300 (1.349 to 3.920)	4	I^2=0.0%	⊕⊕⊕⊖ Moderate	Few studies and prevalence. Observational studies.
**Direct Oral Anticoagulation**	201 per 3 018	1 531 per 12 752	OR 0.792 (0.552 to 1.137)	11	I^2=65.7% (-1)	⊕⊕⊖⊖ Low	Heterogenous comparators. Observational studies, medium heterogeneity.
**GCS Score <15**	2 589 per 8 150	4 104 per 42 351	OR 4.884 (3.141 to 7.594)	38	I^2=97.0% (-1)	⊕⊕⊖⊖ Low	Observational studies, high heterogeneity.
**Headache**	1 097 per 10 485	3 283 per 10 485	OR 1.597 (1.176 to 2.168)	30	I^2=91.1% (-1)	⊕⊕⊖⊖ Low	Heterogenous risk factor definition. Observational studies, high heterogeneity.
**Intoxication**	1 360 per 10 236	3 634 per 10 236	OR 1.241 (0.945 to 1.631)	25	I^2=88.6% (-1)	⊕⊕⊖⊖ Low	Heterogenous risk factor definition. Observational studies, high heterogeneity.

**Male Sex**	2 203 per 9 827	1 449 per 8 191	OR 1.116 (0.934 to 1.334)	21	I^2=69.2%	⊕⊕⊕⊖ Moderate	Observational studies, moderate heterogeneity.
**Loss of Consciousness**	3 774 per 26 276	2 735 per 43 052	OR 2.401 (1.923 to 2.998)	34	I^2=90.0% (-1)	⊕⊕⊖⊖ Low	Heterogenous risk factor definition. Observational studies, high heterogeneity.
**Neurological Deficit**	1 329 per 5 588	2 125 per 38 651	OR 6.224 (2.381 to 16.274)	19	I^2=90.0% (-1)	⊕⊕⊖⊖ Low	Heterogenous risk factor definition. Observational studies, high heterogeneity.
**Post-traumatic Amnesia**	1 569 per 13 384	1 754 per 25 544	OR 2.392 (1.897 to 3.016)	29	I^2=82.1% (-1)	⊕⊕⊖⊖ Low	Observational studies, high heterogeneity.
**Repeated Vomiting**	319 per 1 772	1 571 per 20 026	OR 2.513 (1.673 to 3.776)	7	I^2=82.6% (-1)	⊕⊕⊖⊖ Low	Observational studies, high heterogeneity.
**Scalp Wound**	737 per 7 309	569 per 10 897	OR 2.515 (1.625 to 3.892)	5	I^2=80.4% (-1)	⊕⊕⊖⊖ Low	Observational studies, high heterogeneity.
**Post-traumatic Seizure**	187 per 1 092	3 530 per 46 408	OR 2.303 (1.443 to 3.677)	22	I^2=73.5% (-1)	⊕⊕⊖⊖ Low	Uncertain risk factor definition. Observational studies, moderate heterogeneity.
**Signs of Injury Above Clavicle**	1 083 per 11 459	443 per 9 818	OR 1.920 (1.223 to 3.013)	10	I^2=84.5% (-1)	⊕⊕⊖⊖ Low	Unspecific risk factor. Observational studies, high heterogeneity.
**Signs of Skull Fracture**	1 394 per 2 511	2 929 per 43 177	OR 12.976 (8.174 to 20.601)	18	I^2=93.8% (-1)	⊕⊕⊖⊖ Low	Heterogenous risk factor definition. Observational studies, high heterogeneity.
**Vitamin K Antagonist**	521 per 4 649	1 737 per 18 170	OR 1.534 (0.991 to 2.375)	17	I^2=88.6% (-1)	⊕⊕⊖⊖ Low	Heterogenous comparators. Observational studies, high heterogeneity.
**Vomiting Once**	1 075 per 2 667	2 466 per 27 608	OR 2.991 (1.960 to 4.564)	21	I^2=92.9% (-1)	⊕⊕⊖⊖ Low	Observational studies, high heterogeneity.

High = This research provides a very good indication of the likely effect. The likelihood that the effect will be substantially different‡ is low.
Moderate = This research provides a good indication of the likely effect. The likelihood that the effect will be substantially different‡ is moderate.
Low = This research provides some indication of the likely effect. However, the likelihood that it will be substantially different‡ is high.
Very low = This research does not provide a reliable indication of the likely effect. The likelihood that the effect will be substantially different‡ is very high.
‡ Substantially different = a large enough difference that it might affect a decision

Risk factors for neurosurgical intervention in emergency department head injury patients

Patients: Adult mild head injury or mTBI patients
Setting: Emergency department
Exposure: Risk factor for intracranial injury
Comparison: Control group without presence of the risk factor
Outcome: Emergency neurosurgical intervention due to TBI

Risk factors	Frequency of outcome		Odds ratio (95% CI)	Number of studies	Additional information	Certainty of the evidence (GRADE)	Rationale
	With risk factor	Without risk factor					
**Anticoagulation**	37 per 280	124 per 1 741	OR 1.993 (1.346 to 2.949)	3	I^2=0.0%	⊕⊕⊖⊖ Low	Heterogenous methodology across studies. Observational studies.
**Antiplatelet Therapy**	79 per 1 802	148 per 3 925	OR 1.613 (0.846 to 3.075)	7	I^2=67.5% (-1)	⊕⊖⊖⊖ Very Low	Heterogenous methodology across studies. Observational studies, moderate heterogeneity.
**Direct Oral Anticoagulation**	8 per 533	164 per 2 454	OR 0.557 (0.265 to 1.171)	3	I^2=0.0%	⊕⊕⊖⊖ Low	Heterogenous methodology across studies. Observational studies.
**Glasgow Coma Scale Score <15**	84 per 823	137 per 2 184	OR 1.668 (1.090 to 2.552)	5	I^2=34.6%	⊕⊕⊖⊖ Low	Heterogenous methodology across studies. Observational studies, low heterogeneity.
**Male Sex**	155 per 1 624	67 per 1 070	OR 1.462 (1.017 to 2.102)	5	I^2=17.7%	⊕⊕⊖⊖ Low	Heterogenous methodology across studies. Observational studies, low heterogeneity.
**Vitamin K Antagonist**	34 per 450	178 per 2 995	OR 1.735 (0.683 to 4.409)	5	I^2=60.3%	⊕⊕⊖⊖ Low	Heterogenous methodology across studies. Observational studies, moderate heterogeneity.

High = This research provides a very good indication of the likely effect. The likelihood that the effect will be substantially different‡ is low.
Moderate = This research provides a good indication of the likely effect. The likelihood that the effect will be substantially different‡ is moderate.
Low = This research provides some indication of the likely effect. However, the likelihood that it will be substantially different‡ is high.
Very low = This research does not provide a reliable indication of the likely effect. The likelihood that the effect will be substantially different‡ is very high.
‡ Substantially different = a large enough difference that it might affect a decision

13. Summary of the Modified Delphi Process (Quantitative Data)

Specialization	Country	Recommendation 1	Recommendation 2	Recommendation 3	Recommendation 4	Recommendation 5	Recommendation 6	Recommendation 7
Emergency Medicine	Sweden	6	6	7	6	5	7	7
Neurology	Finland	6	6	7	6	6	7	1
Neurosurgery	Sweden	6	7	7	6	5	6	6
General and Trauma Surgery	Sweden	7	7	7	5	6	7	7
General and Trauma Surgery	Sweden	7	7	7	6	6	7	7
Neurointensive Care	Finland	6	6	7	5	5	7	6
Neurointensive Care	Norway	7	7	7	7	7	7	7
Neurosurgery	Sweden	6	7	7	7	4	7	7
Neurosurgery	Norway	7	5	7	7	6	5	7
Neurointensive Care	Sweden	6	6	7	6	6	7	7
Anesthesiology and Intensive Care	Sweden	7	7	7	7	4	4	7
General and Trauma Surgery	Sweden	6	6	7	5	3	6	7
General and Trauma Surgery	Finland	7	6	7	5	6	7	7
General and Trauma Surgery	Sweden	6	6	5	5	3	6	6
Neurointensive Care	Sweden	7	7	7	7	5	5	6
Pediatric Neurology	Sweden	7	6	7	7	6	6	6
Neurointensive Care	Sweden	6	6	7	7	6	7	7
Neurosurgery	Norway	7	2	7	5	7	7	3
Neurology	Denmark	7	7	7	3	3	6	6
Neurosurgery	Norway	7	2	7	7	5	7	6
Emergency Medicine	Sweden	5	2	7	5	6	6	5
Neurosurgery	Finland	6	6	7	2	5	6	5
Emergency Medicine	Sweden	6	2	5	5	5	3	5
Emergency Medicine	Sweden	6	4	7	5	5	6	6
Emergency Medicine	Denmark	6	2	6	2	6	6	6
Neurosurgery	Norway	6	6	6	6	5	6	7
Geriatrics	Sweden	6	7	6	6	4	4	6
Emergency Medicine	Denmark	5	1	7	2	5	6	4
Rehabilitation Medicine	Sweden	6	6	7	6	3	7	4
Agreement Level (29 Participants)		100%	75.8%	100%	86.2%	75.9%	89.6%	86.2

14. List of Modified Delphi Process Participants

Stakeholders:

Annika Fogle Arvidsson

Mika Martikainen

Johan Viberg

Magnus Halldén

Terhi Lohela

Torbjørn Skodvin

Max Klasén

Carl-Jacob Khailat Holmberg

Ville Vänni

Olivia Kiwanuka

Johanna Savilampi

Dhanushan Dhayalan

Aqeel Chaudry

Carl Tagesson

Lars André

Christian Engvall

Lisbeth Schjerling

Cathrine Tverdal

Johan Herlin Ejerhed

Marie-Laure Bouchy Jacobsson

Kajsa Johansson

Steven Haugbøl

Scandinavian Neurotrauma Committee Members:

Niklas Marklund

Shirin Frisvold

Andras Buki

Fredrik Wickbom

Olga Calcagnile

Fredrik Ginstman

Teemu Luoto

15. Variable Definitions and Harmonisation

Candidate predictors and operational definitions

Variable	Harmonised definition used for synthesis	Accepted source-study terminology or mapping	Important qualifications and expected heterogeneity
Age	Patient age at presentation, analysed according to the age threshold specified by the source study	Older age, elderly patient, age above a specified threshold, commonly ≥60 or ≥65 years	Age thresholds were not assumed to be interchangeable. Analyses were stratified by threshold where sufficient data were available; otherwise, threshold variation was recorded as a source of heterogeneity.
Sex	Sex recorded in the source study, ordinarily analysed as male versus female	Male sex, biological sex	Definitions were accepted as reported. Studies generally did not distinguish sex assigned at birth from gender identity.
Glasgow Coma Scale (GCS) score	Initial or admission GCS following head injury and before sedation or intubation, where reported	GCS 13, GCS 14, GCS 15, impaired GCS, reduced level of consciousness	GCS categories were analysed separately where possible. A GCS score attributable exclusively to a documented pre-injury baseline impairment was not considered a post-traumatic predictor when this distinction was available. Timing of GCS assessment varied between studies.
Loss of consciousness	Reported or witnessed transient loss of consciousness temporally associated with the injury	Loss of consciousness, suspected loss of consciousness, witnessed loss of consciousness, brief unconsciousness, blackout	Definitions and duration thresholds varied. Suspected and confirmed loss of consciousness were combined only when studies did not report them separately.
Post-traumatic amnesia	Inability to recall events immediately before or after the injury, as defined by the source study	Amnesia, post-traumatic amnesia, anterograde amnesia, retrograde amnesia, memory deficit	Duration and assessment methods varied substantially. Retrograde and anterograde amnesia were combined only when reported as a single predictor by the source study.
Headache	Headache reported after the head injury during the initial assessment	Headache, severe headache, persistent headache, worsening headache	Differences in severity, duration, and progression were retained as study-level variation. “Severe” or “worsening” headache was not assumed to be equivalent to headache of any severity when separately reported.
Vomiting once and repeated vomiting	One or more episodes of vomiting following the injury	Vomiting, emesis, repeated vomiting, two or more episodes of vomiting	Threshold-specific analyses were performed for single and repeated episodes. Nausea without vomiting was not mapped to this predictor.
Post-traumatic seizure	A seizure or convulsive event reported to have occurred after the head injury and before or during initial assessment	Post-traumatic seizure, traumatic seizure, convulsion after injury, impact seizure when not separately classified	Isolated impact convulsions were retained separately where the source study distinguished them. Where the temporal sequence was unclear, the source study’s classification was used. Patients whose seizure clearly preceded and caused the trauma were not regarded as having a post-traumatic seizure when this distinction was available.
Focal neurological deficit	A new focal neurological abnormality following the injury	Unilateral motor weakness, unilateral sensory deficit, dysphasia or aphasia, visual field disturbance, cranial nerve deficit, focal neurology	Deficits known to be present before injury were excluded where the source data allowed this distinction. Altered consciousness without focal findings was not included in this category.
Clinical signs of skull fracture	Examination findings suggestive of a depressed, open, or basilar skull fracture	Palpable skull defect, suspected open or depressed fracture, cerebrospinal fluid leakage, haemotympanum, Battle sign, periorbital ecchymosis or “raccoon eyes”	Signs of basilar and depressed skull fracture were combined as “signs of skull fracture” only when studies did not report them separately. A fracture identified solely by imaging was an outcome finding and not a pre-imaging predictor.
Scalp wound	A traumatic wound of the scalp above the eyebrow level requiring clinical assessment or treatment	Scalp laceration, scalp wound, sutured scalp injury, scalp haematoma, wound above the eyebrows	Scalp haematoma was included if the source study used a broader composite definition that could not be separated. The requirement for suturing or other treatment varied across studies.
Visible trauma above the clavicles	Physical evidence of trauma to the head, face, or neck above the clavicles	Trauma above the clavicles, injury above the clavicles, facial injury, external signs of head or facial trauma	This was treated as a broader construct than scalp wound. It was not mapped to scalp wound when the underlying anatomical information was unavailable.
Dangerous or high-energy injury mechanism	An injury mechanism classified as high risk according to the source study or an established decision rule	Pedestrian or cyclist struck by a motor vehicle, occupant ejected from a vehicle, fall from elevation, high-energy road traffic collision, dangerous mechanism	Definitions varied materially between decision rules. Study-specific criteria were retained, and differently defined composite mechanisms were pooled only when considered clinically comparable.
Alcohol or drug intoxication	Clinical or laboratory evidence of intoxication at initial assessment	Alcohol intoxication, drug intoxication, intoxication, elevated blood alcohol concentration	Clinical assessment and laboratory thresholds varied. Prescribed sedative use without evidence of intoxication was not automatically included.
Pre-injury anticoagulant therapy, any	Therapeutic use of an oral or parenteral anticoagulant at the time of injury	Anticoagulation, oral anticoagulation, warfarin, vitamin K antagonist, direct oral anticoagulant, heparin or low-molecular-weight heparin	Prophylactic and therapeutic dosing were distinguished where possible. Studies reporting mixed anticoagulant classes without class-specific results contributed only to the “any anticoagulation” synthesis.
Vitamin K antagonist therapy	Pre-injury treatment with warfarin or another vitamin K antagonist	Warfarin, vitamin K antagonist, coumarin derivative	International normalised ratio thresholds and therapeutic status varied. VKA use was analysed separately from direct oral anticoagulant use where data permitted.
Direct oral anticoagulant therapy (DOAC)	Pre-injury treatment with a direct factor Xa inhibitor or direct thrombin inhibitor	Apixaban, rivaroxaban, edoxaban, dabigatran, DOAC, NOAC	Individual agents were combined into the DOAC category unless agent-specific synthesis was possible. Timing of the last dose, adherence, renal function, and measured anticoagulant activity were inconsistently reported.
Pre-injury antiplatelet therapy, any	Use of at least one platelet-inhibiting medication at the time of injury	Aspirin, acetylsalicylic acid, clopidogrel, ticagrelor, prasugrel, antiplatelet therapy	Antiplatelet monotherapy and dual antiplatelet therapy were separated where possible. Non-prescription or intermittent aspirin use was classified according to the source study.
Antiplatelet monotherapy	Treatment with one antiplatelet agent at the time of injury	Aspirin alone, clopidogrel alone, other single-agent antiplatelet therapy	Studies that combined monotherapy with dual therapy without separable data contributed only to the broader “any antiplatelet therapy” category.

Dual antiplatelet therapy (DAPT)	Concurrent treatment with two platelet-inhibiting agents at the time of injury	Aspirin plus clopidogrel, aspirin plus ticagrelor, aspirin plus prasugrel, DAPT	The specific drug combination and indications for therapy varied. Dual therapy was kept distinct from antiplatelet monotherapy whenever possible.
Coagulopathy	A pre-existing or laboratory-defined impairment of coagulation reported by the source study	Bleeding disorder, clotting disorder, coagulopathy, elevated international normalised ratio, thrombocytopenia	Medication-associated anticoagulation was not included in this category when reported separately. Laboratory thresholds and underlying causes varied and were recorded as potential sources of heterogeneity.
Intraventricular shunt	Presence of a ventricular cerebrospinal-fluid shunt at the time of injury	Ventriculoperitoneal shunt, ventriculoatrial shunt, ventricular shunt, CSF shunt	This predictor was considered separately from clinical suspicion of acute shunt malfunction or mechanical disconnection. No assumption was made that shunt presence alone indicated malfunction.
Previous neurosurgical procedure	History of cranial neurosurgery before the index injury	Previous craniotomy, previous intracranial surgery, cranial operation	Included only when reported as a distinct candidate predictor. Prior surgery was not considered equivalent to the presence of a ventricular shunt.
Bleeding tendency or bleeding disorder	Known condition associated with increased bleeding risk, excluding medication exposure when reported separately	Haemophilia, von Willebrand disease, bleeding diathesis, known bleeding disorder	Definitions were accepted as reported because individual disorders were uncommon. Medication-related bleeding risk was analysed under the relevant medication category.

Non-baseline predictor variables

Variable	Classification in the review	Rationale
S100B concentration	Diagnostic biomarker, analysed separately from routine clinical predictors	Requires blood sampling and an assay-specific threshold. Performance depends on sampling time, age, extracranial injury, and assay characteristics.
GFAP concentration	Diagnostic biomarker, analysed separately	Requires blood sampling and a prespecified assay threshold. Biomarker performance may vary with sampling time, age, injury type, and assay platform.
UCH-L1 concentration	Diagnostic biomarker, analysed separately	Considered alone or in combination with GFAP according to the assay and source study.
CT (computed tomography) finding	Outcome or post-index-test finding	Not available before radiological assessment and therefore not eligible as a pre-imaging predictor for PECO 1.
Repeat CT finding	Follow-up outcome	Used to identify new or progressive radiological injury where relevant, not as a baseline predictor.
Neurosurgical intervention	Outcome	Represented the principal actionable outcome for PECO 2 and was not analysed as a candidate predictor.

Harmonised outcome definitions

Outcome	Harmonised definition	Included source-study outcomes	Qualifications
Acute traumatic intracranial injury on CT	An acute traumatic structural intracranial abnormality identified on non-contrast head CT following the index injury	Intracranial haemorrhage, subdural haematoma, epidural haematoma, traumatic subarachnoid haemorrhage, intraparenchymal haemorrhage or contusion, and other acute traumatic intracranial findings as defined by the source study	Isolated extracranial injuries were excluded. Skull fracture was included only where the source study incorporated it within a composite traumatic CT outcome, such studies were identified as using a broader outcome definition.
Acute neurosurgical intervention	An acute invasive cranial procedure undertaken to prevent or treat neurological deterioration or death attributable to traumatic intracranial injury	Haematoma evacuation, craniotomy or craniectomy, insertion of an intracranial pressure monitor, external ventricular drainage, or another acute cranial procedure as defined by the source study	Admission, neurological observation, specialist consultation, anticonvulsant treatment, reversal of anticoagulation, and repeat CT alone were not classified as neurosurgical intervention. Thresholds for intervention were expected to vary between centres and patient populations.

Mapping of study-level variables

1. Study-reported definitions were retained.
   The review team did not retrospectively redefine individual participants using criteria unavailable in the source publication.
2. Clinically distinct variables were not merged solely to increase sample size.
   For example, scalp wound, scalp haematoma, and any trauma above the clavicles were retained as separate constructs where the published data allowed this distinction.
3. Threshold-dependent predictors were harmonised cautiously.
   Age, vomiting frequency, amnesia duration, injury mechanism, and laboratory definitions of coagulopathy were pooled only when the thresholds were considered sufficiently comparable. Differences in thresholds were documented and considered when assessing clinical heterogeneity and GRADE certainty.
4. Medication classes were analysed hierarchically.

Data were extracted for any anticoagulation, vitamin K antagonists, direct oral anticoagulants, any antiplatelet therapy, antiplatelet monotherapy, and dual antiplatelet therapy. A study contributed to the most specific available category. Broader and class-specific estimates from the same participants were not entered into the same pooled analysis.

5. Composite predictors were not disaggregated without source data.
   If a study reported only a composite predictor, such as "coagulopathy or anticoagulant use," it was not assigned to an individual component unless component-specific data were available.
6. Unreported variables were coded as missing rather than absent.
7. Adjusted and unadjusted estimates were distinguished.
   The primary binary meta-analysis was based on study-level event counts for patients with and without each predictor. Adjusted estimates reported by individual studies were extracted separately, where available, and used for contextual interpretation rather than combined with unadjusted estimates in the same synthesis.
8. Overlapping cohorts were avoided.
   When two or more publications described overlapping populations, the publication with the most complete, relevant, or recent dataset was selected for the relevant synthesis unless non-overlapping subgroups could be identified.
9. Multiple predictor strata were collapsed only when appropriate.
   Where mutually exclusive exposure strata represented the same harmonised predictor, event counts and denominators were summed to generate one exposed group. Participants were included only once in each synthesis.
10. Ambiguous definitions were resolved by consensus.
    Uncertain mappings were reviewed by at least two members of the review team. Variables were excluded from quantitative synthesis when clinically defensible harmonisation could not be achieved.

16. Evidentiary Table 1

Clinical Question 1: "Which adult emergency department patients with minimal or mild head injury need a head computed tomography and which patients may be directly discharged?"

Study	Year	Country	Design	n	Age	GCS	Other inclusion criteria	Exclusion criteria	CT%	Follow up	Evidence level (CEBM)	Limitations and comments	Outcomes	Risk Factors
Allouchery	2018	France	P	1449	>10	13-15	Antiplatelet treatment, LOC, PTA, 3 hours from injury with S100B sampling	mTBI with no medium or high risk factors	70	No	3	SB. S100B threshold 0.10 µg/L for adults and 0.18 µg/L for teenagers 10-18 years of age	Intracerebral lesion	S100B
Alrajhi	2015	Canada	R	176	25-100	13-15	GCS 13-15, Warfarinized, INR>1.5	Penetrating injury, neurological deficit, structural brain abnormality, referrals and repeat visits	100	No	3	SB. Warfarinized cohort.	Intracranial hemorrhage	Male sex, headache, LOC, GCS<15, PTA, vomiting, signs of skull fracture
Asadollahi	2015	Iran	P	158	≥18	13-15	LOC, PTA	GCS 15 without LOC or PTA, pregnancy, neurologic or psychiatric disorder, focal neurological deficit, multitrauma, renal or liver disease, AIS≥2, non-traumatic lesions	100	No	4	SB. Brain edema defined as a positive CT finding. S100B thresholds 0.115 µg/L and 0.210 µg/L tested.	Intracerebral lesion	S100B, male sex, fall, anticoagulation, intoxication, LOC, PTA, headache, repeated vomiting, seizure
Bazarian	2021	Multinational Cohort	P	1901	18-98	13-15	Non-penetrating head injury, CT performed, blood sample collected	Withdrawn, blood sample lost, CT inconclusive	100	No	3	SB. Cohort includes only patients with biomarker sampling.	Traumatic intracranial injury	GPAF/UCH-L1
Calcagnile	2016	Sweden	P	726	>18	14-15	LOC, PTA	Focal neurology, non-Swedish citizens, S100B sampling >3h	55.8	3 months after injury	3	SB.	Traumatic intracranial injury	S100B
Chayoua	2024	The Netherlands	P	253	>18	13-15	LOC, PTA	Neurological or psychiatric comorbidity, prior admission for TBI, drug abuse, mental disability, language barriers.	100	No	3	SB. Cohort includes only patients with biomarker sampling.	Traumatic intracranial injury	Male sex, LOC, GCS<15, PTA, intoxication, GPAF/UCH-L1.
Cipriano	2018	Italy	P	206	>18	13-15	Patients on oral anticoagulation treatment	>48h from trauma, no anticoagulation effect	100	Yes, 30 days after the traumatic injury	3	SB. Cohort includes only patients on oral anticoagulation treatment.	Immediate and delayed traumatic intracranial hemorrhage	Male sex, DOAC treatment, high energy trauma, LOC, PTA, antiplatelet
Colas	2023	France	P	840	>18	13-15	Patients on antithrombotic treatment		100	No	3	SB. Cohort of patients all on antithrombotic medication.	Traumatic intracranial hemorrhage	Antiplatelet, VKA, DOAC, male sex, high energy trauma, GCS<15, LOC, PTA, intoxication, signs of skull base fracture
Dupuis	2023	France	R	1001	>65	mTBI		Pre-existing neurological conditions, hospitalized patients, polytrauma,	100	Hospitalization, neurosurgical intervention	3	SB.	Traumatic intracranial hemorrhage	Male sex, GCS<15, neurological deficit

								degraded neurological status prior to trauma						
Foks	2019	The Netherlands	P	3914	>16	15	Blunt head trauma, <24h from injury	Patients transferred from other hospitals	79	No	3	SB.	Traumatic intracranial injury	LOC, PTA
Galliazzo	2019	Italy	R	1846	>18	13-15	mTBI patients	LMWH treated patients	75	No	3	SB.	Traumatic intracranial hemorrhage	Antiplatelet, VKA, DOAC, DAPT, male sex, GCS<15, LOC, PTA, neurological deficit, seizure, headache, vomiting, intoxication, signs of skull base fracture, high energy trauma, scalp wound
Gill	2018	US	P	277	18-85	13-15	Blood collection and radiology within 48 hours of injury		100	No	3	SB. CT and MRI performed.	Traumatic intracranial injury	Male sex, high energy trauma
Hofmann	2021	Austria	R	1129	75-105	mTBI	Admitted patients >75 years on antithrombotic or antiplatelet medication, low impact fall, two or more CT scans	Polytrauma, high impact trauma, one CT only	100	No	4	SB. Cohort with age threshold, admission, antithrombotic medication and specific traumatic mechanism	Traumatic intracranial hemorrhage	Antiplatelet, VKA, DOAC
Hosseininejad	2023	Iran	P	74		14-15	Mild head trauma	Moderate or severe TBI	100	No	4	SB.	Traumatic intracranial injury	Male sex, vomiting, GCS<15, headache
Innocenti	2017	Italy	R	478		13-15	Admitted to emergency department high dependency unit for mTBI		100	Follow up at 6 months	4	SB.	Traumatic intracranial injury	Male sex, intoxication, high energy trauma
Jame	2014	Iran	P	400	>3	13-15	Mild head trauma within 12 hours		100	No	4	SB.	Abnormal CT results	High energy trauma, signs of skull base fracture, repeated vomiting, age>65, sign of trauma above clavicle, seizure, PTA
Kahouadji	2020	Switzerland	P	130	>18	13-15	Mild head trauma with indication for head CT in accordance with the Canadian CT head rule		100	No	3	SB.	Abnormal CT results	S100B
Lagerstedt	2018	Multinational Cohort	P	132	>14	15	Mild traumatic brain injury and one additional clinical symptom	CT performed >24 hours from trauma, pregnancy, no consent	100	No	4	SB.	Traumatic intracranial injury	PTA, LOC, vomiting, headache
Leitner	2021	Austria	R	1788	18-103	13-15	Mild traumatic brain injury with indication for CT screening based on the German Society of Neurosurgery guidelines, hospitalized patients		96	30 days and additional follow-up for mortality	4	SB. Hospitalized patients only.	Traumatic intracranial hemorrhage	Male sex, anticoagulation, DAPT, antiplatelet, VKA, DOAC, age
Levine	2014	US	R	658	>14	15	Minor head trauma patients who have undergone a CT	Pre-injury Warfarin use, multiple visits, pediatric patients,	100	No	4	SB.	Traumatic intracranial hemorrhage	Antiplatelet (clopidogrel)

								altered mental status						
Martínez-Rivas	2023	Spain	R	907	All	13-15	Mild traumatic brain injury patients		100	No	3	SB.	Traumatic intracranial hemorrhage	PTA, vomiting, headache, seizures, anticoagulation, antiplatelet, VKA, DOAC
Mishra	2017	India	P	453	All	15	Traumatic brain injury patients with GCS 15		100	No	4	SB. GCS 15 patients only.	Abnormal CT results	Age, male sex, high energy trauma, LOC, vomiting, seizure
Molaei-Langroudi	2019	Iran	P	610	>2	13-15	Blunt traumatic brain injury with head CT scan performed within 24 hours	Hemodynamic instability, complications requiring special care, opium addiction, no consent	100	No	4	SB.	Traumatic intracranial injury	GCS<15, high energy trauma, headache, vomiting, skull fracture, injury above clavicle, intoxication, LOC, neurological deficit, seizure
Niklasson	2024	Sweden	R	4850	>18		Presenting to ED with head trauma	Return visits, duplicate or missing records, non-medically managed patients, no head trauma	100	No	3	Probable SB.	Traumatic intracranial hemorrhage	Antiplatelet, VKA, DOAC, DAPT.
Nishijima	2013	US	P	982	>18	13-15	Mild blunt head trauma, on warfarin or clopidogrel, undergone CT	Transfer from outside facilities	100	No	3	SB. All patients on warfarin on clopidogrel.	Traumatic intracranial hemorrhage	Age, VKA, antiplatelet, high energy trauma, headache, intoxication, trauma above clavicles, GCS<15
Oris	2021	France	P	1172	>65	13-15	Mild traumatic brain injury with medium risk, blood sampling within 3 hours	Mild traumatic brain injury with high or low risk of complications, missing information sheet	82	No	3	Probable SB. Partial overlap in cohort with Allouchery 2018.	Abnormal CT results	S100B
Sakkas	2024	Germany	R	696	>18	13-15	Mild traumatic brain injury patients on antithrombotic medication		100	No	3	SB. All patients on antithrombotic medication.	Traumatic intracranial hemorrhage	Antiplatelet, VKA, DOAC, DAPT, age, male sex, high energy trauma, GCS<15, LOC, headache, PTA, neurological deficit, seizure, skull fracture
Savioli	2020	Italy	R	1677	>18	14-15	Mild head trauma		100	No	3	Probable SB.	Traumatic intracranial hemorrhage	DOAC, VKA
Schindler	2023	Germany	R	227	>65		Minor head trauma, ground level fall, preinjury anticoagulation and CT performed	Patients with severe injuries managed from the trauma bay	100	No	4	SB. Age threshold, all patients anticoagulated, ground level fall only. Absence of clear numerical report of findings.	Traumatic intracranial hemorrhage	Antiplatelet (ASA), DOAC
Shafie	2023	Iran	P	498	All	13-15	Head injury or scalp wound suggesting head injury, <30 minutes of LOC, <24 hours of PTA	Refusal to participate, intoxication	100	No	3	Probable SB. Edema among outcome CT findings.	Traumatic intracranial hemorrhage, skull fracture or edema	Male sex, high energy trauma, anticoagulation, antiplatelet, vomiting, LOC, PTA, seizure, skull fracture, headache, GCS<15

Singh	2023	India	P	100	>16	13-15	Head injury, <30 minutes of LOC, <24 hours of PTA, impaired mental state, transient neurological deficit		100	No	3	Probable SB.	Significant CT findings (SDH, EDH, SAH, contusion, depressed/basal skull fracture), insignificant CT findings (soft tissue swelling, linear skull fractures, maxillofacial fractures)	GCS<15, age
Sugimoto	2017	Japan	R	73	All	14-15	mTBI patients who underwent head CT	Antiplatelet, anticoagulant, multitrauma, seizure, history of neurosurgery	100	No	3	SB.	Intracranial structural disorder	Male sex, GCS<15, high energy trauma
Teeratakulpisarn	2021	Thailand	R	100	>18	13-15	mTBI patients with high risk of ICH	Non-traumatic abnormal CT findings	100	No	3	SB due to inclusion criteria.	Traumatic intracranial hemorrhage	Male sex, high energy trauma, LOC
Turcato	2022	Italy	R	3054	All	14-15	mTBI patients on oral anticoagulation	Arrival >48 hours after injury, ineffective anticoagulation, incomplete data, non-residents in the region of the hospital	100	No	3	Probable SB. Outcome presented as immediate ICH combined with delayed ICH.	Global traumatic intracranial hemorrhage (immediate and delayed)	Male sex, VKA, DOAC, high energy trauma, intoxication, antiplatelet, LOC, PTA, headache, GCS<15, neurological deficit, trauma above clavicles, vomiting, seizure
Vaniyapong	2020	Thailand	P	1164	>16	13-15	Blunt head injury, one of: headache, vomiting, LOC, PTA, seizure, intoxication, previous procedure, anticoagulant use, sign of skull fracture or scalp wound	Uncertain history of trauma, time from injury >24h	39.3	Clinical follow-up 7 days from injury	3	Probable SB.	Traumatic intracranial injury	Age, male sex, GCS<15, high energy trauma, intoxication, headache, vomiting, LOC, PTA, seizure, anticoagulant, neurological deficit, scalp wound, skull fracture
Yamada	2020	Japan	P	1122	>18	15	Minor head trauma.		93.3	No	3	Probable SB.	Traumatic intracranial hemorrhage	Age, male sex, antiplatelet, anticoagulation, high energy trauma, scalp wound, intoxication, neurological deficit
Yuksen	2018	Thailand	R	708	>15	13-15	Mild traumatic brain injury	No symptoms	100	No	3	Probable SB.	Traumatic intracranial hemorrhage	Age, male sex, GCS<15, skull fracture, vomiting, neurological deficit, seizure, headache, PTA, LOC, antiplatelet, VKA, high energy trauma, intoxication
Zhang	2017	China	R	3142	17-84	13-15	mTBI admitted <24 hours after injury, age >16, undergone CT	Open TBI, incomplete data.	100	No	3	Probable SB.	Traumatic intracranial injury	Male sex, age, intoxication, PTA, vomiting, skull fracture

17. Evidentiary Table 2

Clinical Question 2: "Which adult emergency department patients with minimal or mild head injury need in-hospital observation and/or a repeat head computed tomography?"

Study	Year	Country	Design	n	Age	GCS	Other inclusion criteria	Exclusion criteria	Follow-up	Evidence level	Relevant findings	Limitations and comments	Outcomes	Risk Factors
Antoni	2022	Austria	R	1169	>18	3-15	Patients on antithrombotic therapy, blunt head trauma, initial CT and >24 hours of in-hospital observation	Alcohol intoxication	Until discharge	4	85/1169 initial CT pathologies, 7/1169 delayed ICH, 12/1169 neurosurgery	SB	Initial CT findings, delayed ICH, neurosurgery	DOAC, VKA, antiplatelet
Chojak	2021	US	R	186	19-90	13-15	Conservatively treated mTBI patients at a neurosurgical department	Subdural hematoma, younger than 18, need emergency surgery	Length of stay	4	7/186 with clinical deterioration, 3/186 needed neurosurgical intervention	SB, patients with subdural hematoma and need for emergency surgery excluded	Deterioration, NSI, death	GCS
Della Pepa	2022	Italy	R	299	>18	14-15	LOC, PTA	Immediate surgery, age <18, pregnant women, coagulopathic disease, no CT, negative initial CT			32/299 patients underwent NSI	SB	Initial CT finding, lesion progression, NSI	VKA, DOAC
Joseph	2015	US	R	876	>18	13-15	ICI on initial CT	Antiplatelet/anticoagulation, transferred patients	Length of stay	4	47/876 patients underwent NSI	SB, univariable and multivariable OR reported	CT progression, NSI	Age, sex, intoxication, LOC, CT findings, platelet count
Karanci	2022	Turkey	R	441	0-88	13-15	Repead head CT scan	Only one CT scan, penetrating injuries, non-traumatic cause for CT, repeat CT due to technical issues	None	4	5/441 patients underwent craniotomy, 17/441 patients underwent mannitol treatment	SB, reports mannitol alongside NSI as intervention	ICI, CT progression, NSI/mannitol treatment	GCS, amnesia, skull base fracture, seizure, vomiting, neurological deficit
Martin	2018	US	R	332	>18	All	AIS head score > 0	No traumatic injury	All cause mortality	4	16/332 patients required NSI	SB	NSI	Anticoagulant, antiplatelet
Patel	2023	US	R	1542	>18	13-15	Traumatic ICH on initial CT head	AIS > 1 in any region besides the head, any polytrauma	60-day mortality	3	126/1542 patients required NSI	SB	NSI	Sex, dementia, anticoagulation, DOAC, VKA, antiplatelet, neurological deficit, headache, LOC, seizure, GCS
Savioli	2021	Italy	R	1926	>18	14-15		Suspected or diagnosed skull fracture and spontaneous ICH	30 days after visit	4	12/1926 patients underwent NSI	SB	NSI, ED revisits within 30 days	Antiplatelet
Sumritpradit	2016	Thailand	R	145	>15	All	Traumatic brain injury with repeat CT	Duration of admission >72 hours		4	8/145 patients underwent NSI	SB, repeated CT cohort	NSI	Sex, antiplatelet, VKA
Thorson	2013	US	R	360	>18	13-15	AIS head score > 0	No non-head AIS > 1, penetrating trauma, pregnancy, incarcerated, transfer		4	30/360 patients required NSI	SB	CT progression, NSI	Sex, GCS, VKA, antiplatelet
Tourigny	2021	Canada	R	478	>16	13-15	LOC, PTA, focal neurological deficit or complication in addition to mTBI	Penetrating injury, cerebral tumor or aneurysm found on CT		4	40/478 patients required NSI	SB	NSI	Sex, intoxication, age, PTA, LOS, GCS, headache, vomiting, seizure, VKA, antiplatelet, DOAC
Ward	2022	US	R	234	>18	13-15	AIS head 1-2	Non-blunt mechanism, pregnant, did not undergo a second scan	Discharge disposition, length of stay	4	18/234 required neurosurgical intervention	SB, repeated CT cohort	NSI, medical intervention, CT progression, LOS	Sex, anticoagulation, antiplatelet

18. Notes from the Modified Delphi Process

Recommendation 1
Notes from the modified Delphi process: Stakeholder comments were few, with questions being raised concerning the clinical relevance of GCS score 14 arising from preexisting dementia. While these patients will likely require a case-by-case clinician assessment, the intent of the guideline is clarified to be primarily addressing new onset symptoms, thus an altered GCS score that developed post-traumatically. Another issue raised concerns the timeframe after head injury beyond which emergency CT is no longer considered relevant. This was addressed in the discussion concerning Recommendation 5.

Recommendation 2
Notes from the modified Delphi process: The definition of a scalp wound is questioned and addressed as stated above. A major concern raised by stakeholders is that due to the presence of a high proportion of elderly patients in the Nordic head injury population, applying >65 years of age and antiplatelet therapy may lead to excessive rule-in of patients at low risk of requiring intervention. To mitigate potential overtriage in elderly populations, the guideline emphasises that eligible patients must remain clinically stable and be candidates for emergency surgical or medical interventions.

Recommendation 3
Notes from the modified Delphi process: Definition of focal neurological deficit is requested. This is specified as a new, persisting post-traumatic unilateral motor or sensory dysfunction, dysphasia or visual disturbance and this definition is provided in the help sheet for the updated guideline flowcharts (see Appendix). The question of necessity of admissions for post-traumatic seizures where imaging was negative was also raised. However, the workgroup is insistent that admission remains relevant due to the diagnostic uncertainty involved and the potential risk of deterioration and recurrent seizures.

Recommendation 4
Notes from the modified Delphi process: Comments pertain primarily to limited experience with biomarkers in clinical practice and limited availability of relevant biomarker assays. The workgroup emphasises that the SNC does not endorse any specific biomarker, but flowcharts have been included to account for the possibility of clinical integration where sampling is available (see Appendix).

Recommendation 5
Notes from the modified Delphi process: Stakeholders noted that resource constraints may lead to CT being favored over prolonged observation. A second concern raised is that patients on antithrombotic medication may require radiological guidance to correctly prioritise the pausing or reversal of medication, making observation alone not feasible in a large proportion of scenarios.

Recommendation 6
Notes from the modified Delphi process: Stakeholder feedback include concern regarding to what extent the discharge instructions were applicable to patients who do not have full autonomy at baseline due to pre-existing conditions. An additional comment is that symptoms representative of post-concussive symptoms should be listed more exhaustively, though this may not be feasible to maintain legibility but may be modified in national documentations. Stakeholders also suggested that symptoms of chronic subdural haematoma are not sufficiently described, however the discharge instructions recommend a family doctor or general practitioner assessment upon prolonged or worsening symptoms which provide coverage for potential chronic complications. Stakeholders with expertise in rehabilitation medicine provided evidential guidance and suggestions for modifications to the discharge instructions, specifically the benefit of aerobic exercise and limited utility of additional rest beyond the initial 48-hour window (see references below).

Leddy JJ, Burma JS, Toomey CM, Hayden A, Davis GA, Babl FE, et al. Rest and exercise early after sport-related concussion: a systematic review and meta-analysis. Br J Sports Med. 2023;57(12):762-70.

Mercier LJ, McIntosh SJ, Boucher C, Joyce JM, Batycky J, Galarneau JM, et al. Effect of Aerobic Exercise on Symptom Burden and Quality of Life in Adults With Persisting Post-concussive Symptoms: The ACTBI Randomized Controlled Trial. Arch Phys Med Rehabil. 2025;106(2):195-205.

Recommendation 7
Notes from the modified Delphi process: The pathway into the flowchart is discussed and while the intention is understood, the task of ruling out patients that are not appropriate candidates for emergency intervention is

recognised to be a challenge in the ED setting. The absence of post-traumatic amnesia is questioned. The intent of suspected loss of consciousness as defined by the guideline is intended to overlap with this risk factor. Final concerns raised return to the subject of questioning the placement of age >65, scalp wound and antiplatelet therapy when they are considered less specific by clinicians in comparison to the other predictors.

19. Guideline Flowcharts, Help Sheets and Discharge Information

Presented in the following order:

Flowchart 1: No biomarkers available

Flowchart 2: S100B available

Flowchart 3: GFAP and UCH-L1 available

Flowchart 4: GFAP available

Help Sheet 1: No biomarkers available

Help Sheet 2: S100B available

Help Sheet 3: GFAP and UCH-L1 available

Help Sheet 4: GFAP available

Discharge advice for a person who has had a head injury

SNC 2026 Guideline for Initial Management of Minimal, Mild and Moderate Head Injury in Adults ≥ 18 years old (no biomarkers available)

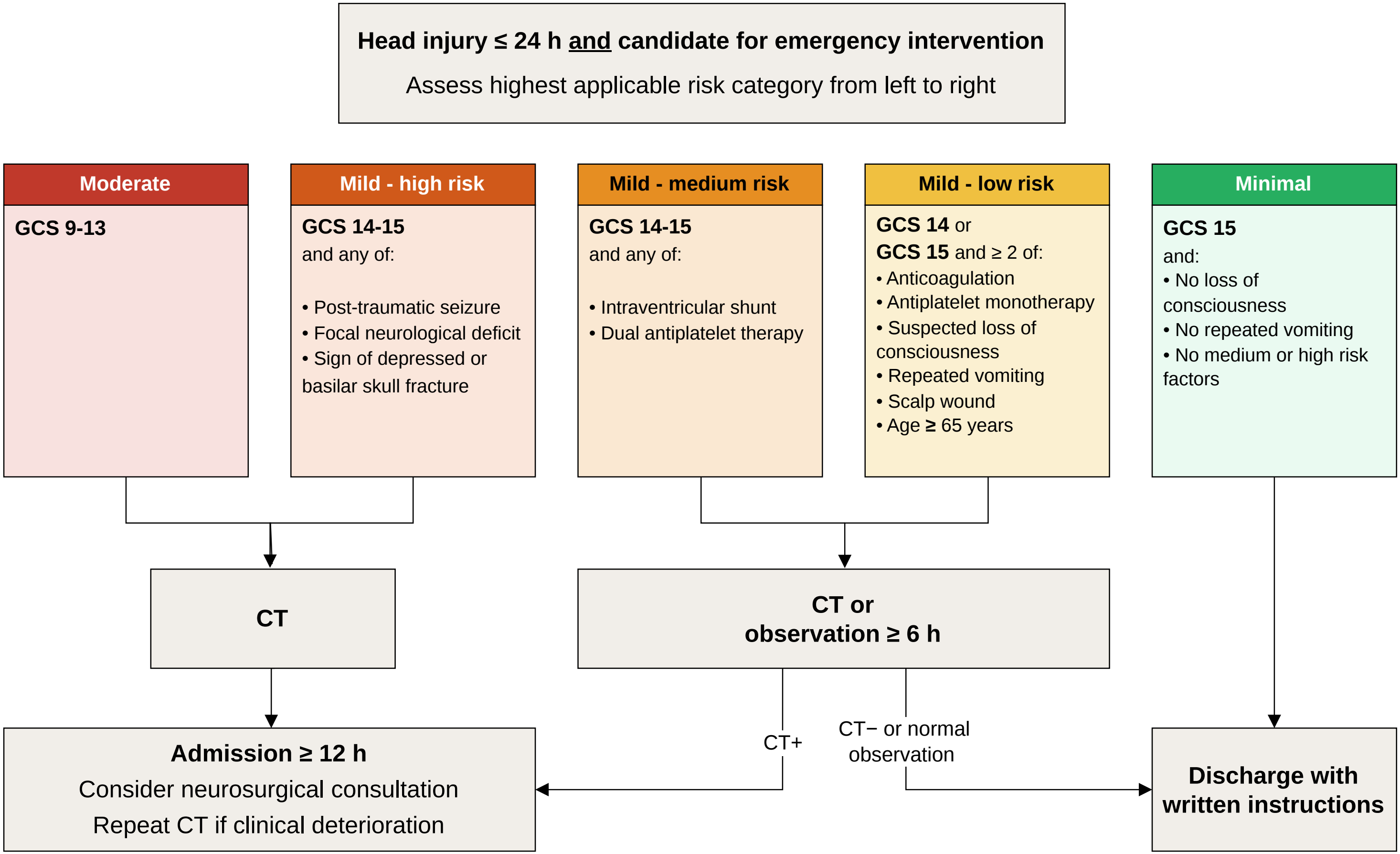

SNC 2026 Guideline for Initial Management of Minimal, Mild and Moderate Head Injury in Adults ≥ 18 years old (S100B available)

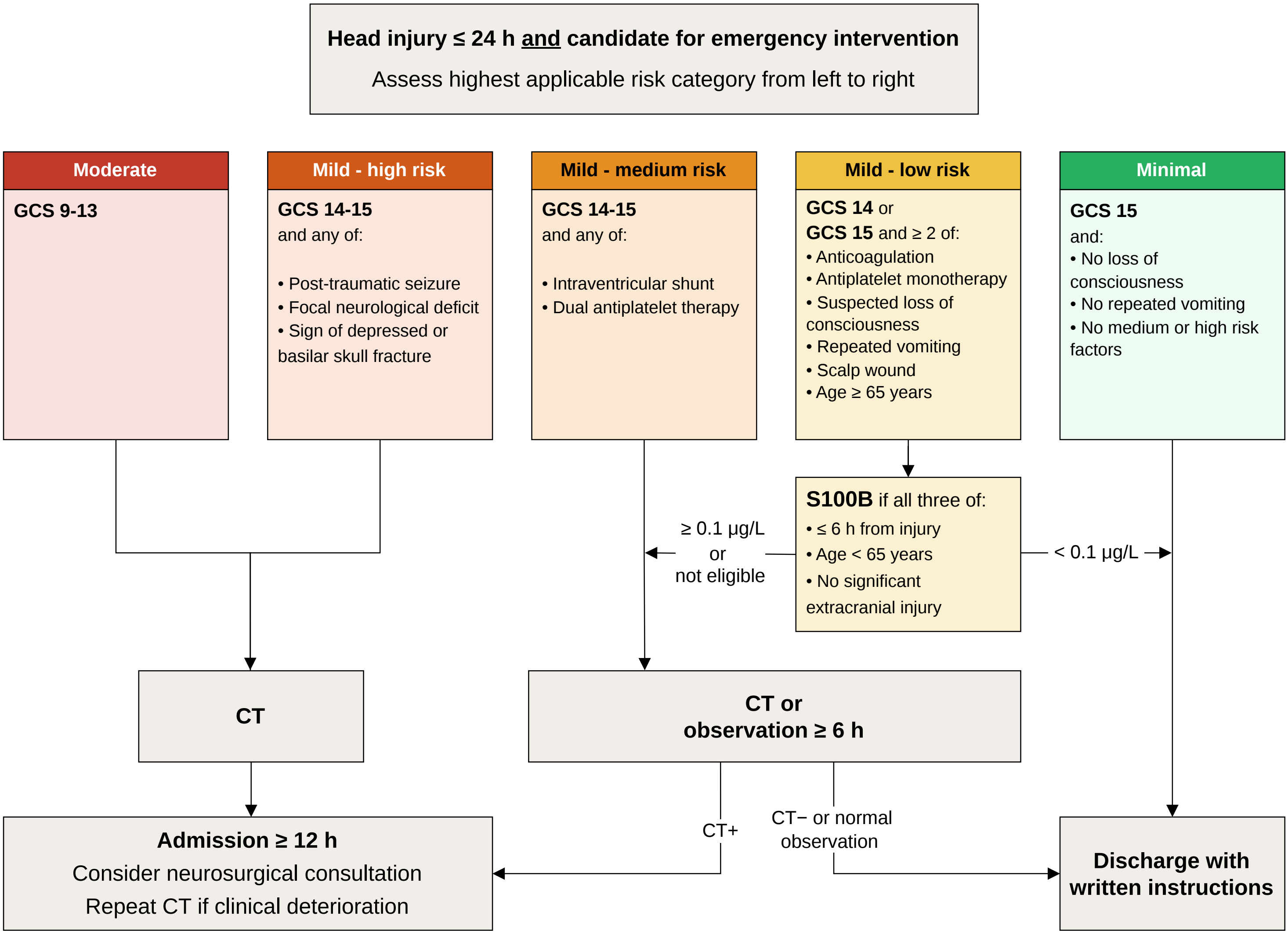

SNC 2026 Guideline for Initial Management of Minimal, Mild and Moderate Head Injury in Adults ≥ 18 years old (GFAP and UCH-L1 available)

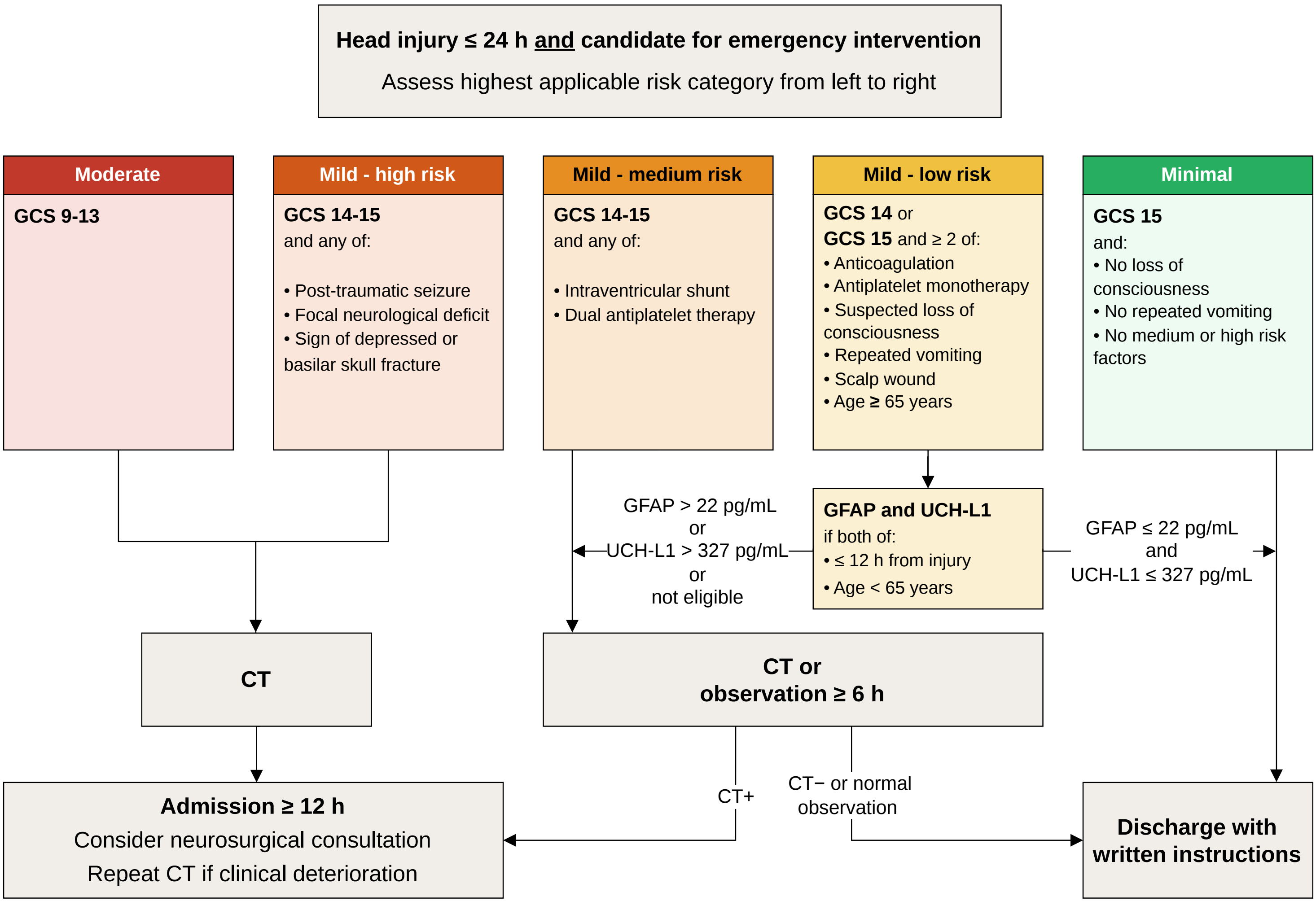

SNC 2026 Guideline for Initial Management of Minimal, Mild and Moderate Head Injury in Adults ≥ 18 years old (GFAP available)

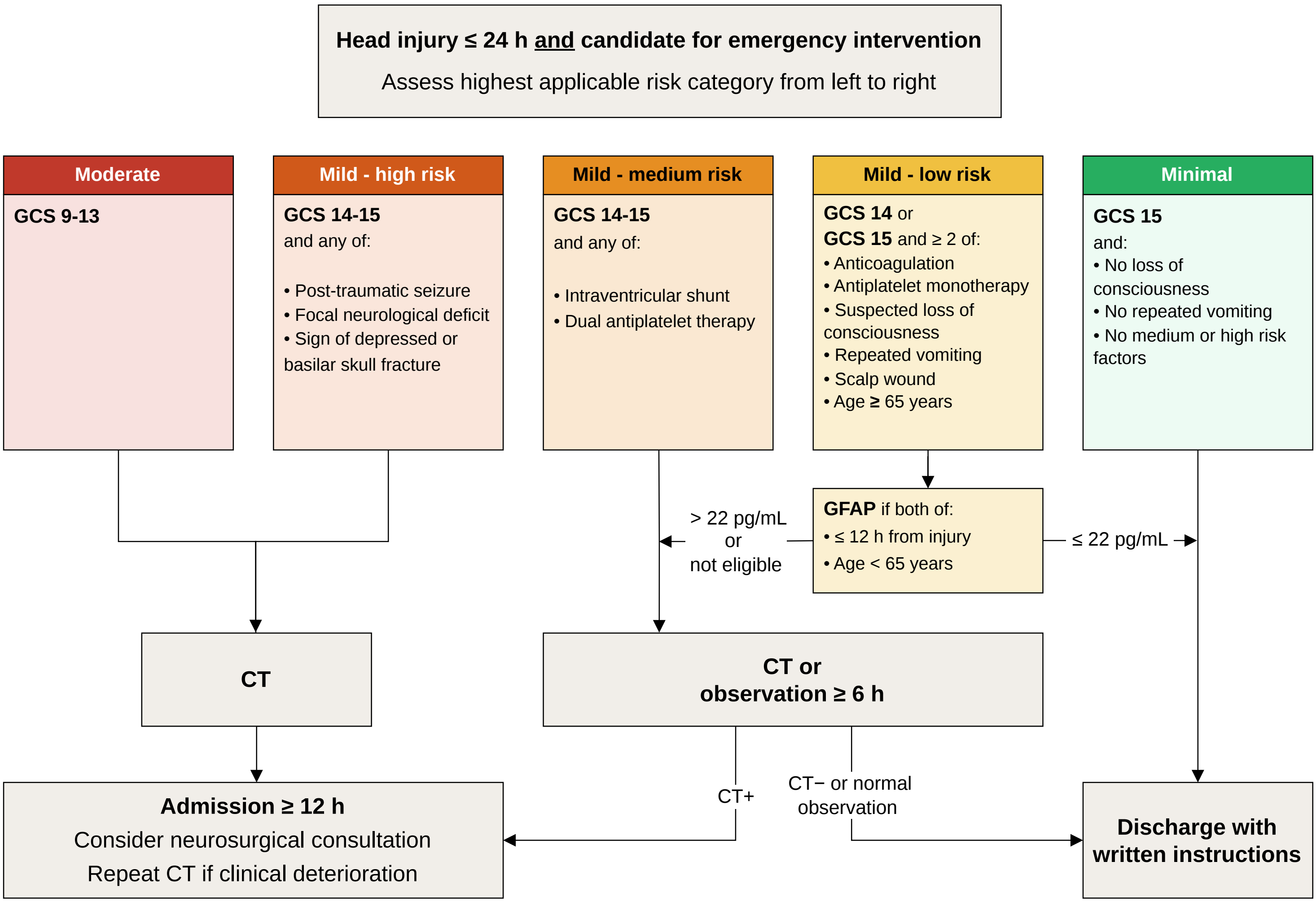

Scandinavian Guidelines for Initial Management of Adult Patients with Minimal, Mild and Moderate Head Injury
Help Sheet (No Biomarkers)

Main Points
This guideline is based upon evidence-derived recommendations and expert consensus from the Scandinavian Neurotrauma Committee (SNC) mTBI working group. Being a guideline, it should not override clinical judgement and may be superseded if necessary. Glasgow Coma Scale (GCS) scores should be noted as the score after resuscitation and reduced GCS scores must be reductions from baseline. A standard clinical exam should be sufficiently comprehensive for the various risk factors to be adequately assessed. This guideline is intended for patients who are candidates for emergency neurosurgical or medical interventions. If the patient is suffering from severe comorbidities and is assessed not to be appropriate for acute neurosurgical intervention, or withdraws consent for further investigation, we do NOT recommend the application of this guideline. The guideline does not apply automatically to every reported contact with the head. Before applying the risk factors, the assessing clinician should determine that the mechanism, symptoms, or uncertainty surrounding the event provides reasonable grounds to suspect a clinically relevant head injury.

Mild - High Risk
These patients have GCS scores 14-15 and have had a post-traumatic seizure, have focal neurological deficits (a new, persisting post-traumatic unilateral motor or sensory dysfunction, dysphasia or visual disturbance), or have clinical signs of a depressed skull fracture (palpable depression or abnormal discontinuity of skull) or a basal skull fracture (raccoon eyes, Battle's sign, haemotympanum, cerebrospinal fluid rhinorrhea). These patients are relatively uncommon but have a relatively high risk of intracranial complications. They should undergo a head computed tomography (CT) and also be admitted for close observation for at least 12 hours even if the CT is normal.

Mild - Medium-Low Risk
These patients have GCS score 14-15 and have an intraventricular shunt or are on dual antiplatelet therapy (DAPT). Alternatively, these patients have GCS score 14 without risk factors or GCS score 15 with at least two of the following: anticoagulation use, antiplatelet use, suspected or confirmed loss of consciousness (i.e. the patient cannot clearly deny loss of consciousness), repeated vomiting, a scalp wound or are aged ≥65. These patients should undergo either observation (see below) or a head CT scan and if the CT is normal, can be discharged if symptoms have been stable or improving.

Minimal
These patients have GCS score 15 and have no loss of consciousness, repeated vomiting, or any medium or high risk factors. They have a very low risk of intracranial complications and can be discharged from the hospital without a CT scan or observation.

Observation and Admission
Observation may be done in-hospital or in the emergency department, depending on local routines. We recommend checking for decreased level of consciousness (GCS) and unilateral extremity weakness (basic neurological exam) every 15 minutes during the first 3 hours post-trauma and every 30 minutes following this. Observation frequency can be tailored to patient status (i.e. stable patients may need less frequent observation whilst unstable (worrying and/or worsening signs/symptoms) may need intensified observation). The recommended minimum observation time is 6 hours.

Some patients with minimal head injury or normal CT following mild head injury may need admission for other reasons than the head injury (such as elderly patients without sufficient help at home, patients with other injuries, intoxicated patients, or patients requiring additional assessment for the underlying cause of trauma such as syncope). Since these patients have a very low risk of intracranial complications, they do not need the extensive observation routine mentioned above.

CT
A non-contrast CT in compliance with local radiology routines should be performed as quickly as possible, with a greater urgency for more severe clinical presentations in accordance with the guideline. The patient should be adequately monitored when waiting for the CT scan. When CT is abnormal, consider contacting a neurosurgeon or neurotrauma center for advice concerning further management. Symptomatic patients with a normal non-contrast head CT may need additional radiology (such as CT angiography for possible traumatic vascular dissections).

Repeat CT
Routine repeat CT is not recommended. However, a repeat CT should be done immediately in patients with deterioration in GCS score (≥2 points) or new/progressive neurological deficits (a new, persisting post-traumatic unilateral motor or sensory dysfunction, dysphasia or visual disturbance).

Discharge
ALL patients with head injury should receive oral and written instructions upon discharge (see separate form). These provide general advice concerning their head injury. Do not discharge a patient with worrying or worsening symptoms (other than typical post-injury issues such as mild headache and nausea). Patients should be discharged at their premorbid neurological and functional baseline and into an appropriate level of supervision or care.

Scandinavian Guidelines for Initial Management of Adult Patients with Minimal, Mild and Moderate Head Injury Help Sheet (S100B Available)

Main Points

This guideline is based upon evidence-derived recommendations and expert consensus from the Scandinavian Neurotrauma Committee (SNC) mTBI working group. Being a guideline, it should not override clinical judgement and may be superseded if necessary. Glasgow Coma Scale (GCS) scores should be noted as the score after resuscitation and reduced GCS scores must be reductions from baseline. A standard clinical exam should be sufficiently comprehensive for the various risk factors to be adequately assessed. This guideline is intended for patients who are candidates for emergency neurosurgical or medical interventions. If the patient is suffering from severe comorbidities and is assessed not to be appropriate for acute neurosurgical intervention, or withdraws consent for further investigation, we do NOT recommend the application of this guideline. The guideline does not apply automatically to every reported contact with the head. Before applying the risk factors, the assessing clinician should determine that the mechanism, symptoms, or uncertainty surrounding the event provides reasonable grounds to suspect a clinically relevant head injury.

Mild - High Risk

These patients have GCS scores 14-15 and have had a post-traumatic seizure, have focal neurological deficits (a new, persisting post-traumatic unilateral motor or sensory dysfunction, dysphasia or visual disturbance), or have clinical signs of a depressed skull fracture (palpable depression or abnormal discontinuity of skull) or a basal skull fracture (raccoon eyes, Battle's sign, haemotympanum, cerebrospinal fluid rhinorrhea). These patients are relatively uncommon but have a relatively high risk of intracranial complications. They should undergo a head computed tomography (CT) and also be admitted for close observation for at least 12 hours even if the CT is normal.

Mild - Medium Risk

These patients have GCS score 14-15 and have an intraventricular shunt or are on dual antiplatelet therapy (DAPT). These patients should undergo a head CT scan and if the CT is normal, can be discharged if symptoms have been stable or improving.

Mild - Low Risk

These patients have GCS score 14 without risk factors or are alternatively GCS score 15 and have at least two of the following: anticoagulation use, antiplatelet use, suspected or confirmed loss of consciousness (i.e. the patient cannot clearly deny loss of consciousness), repeated vomiting, a scalp wound, or age ≥65. If less than 6 hours have passed since the trauma, the patient is <65 years of age and there are no significant extracranial injuries, sample serum S100B. If S100B is <0.10 µg/L, the patient has a very low risk of serious intracranial complications and can be discharged. If S100B is ≥0.10 µg/L, either observation (see below) or a CT scan should be performed. If the CT is normal, the patient can be discharged if symptoms have been stable or improving.

Minimal

These patients have GCS score 15 and have no loss of consciousness, repeated vomiting, or any medium or high risk factors. They have a very low risk of intracranial complications and can be discharged from the hospital without a CT scan or observation.

Observation and Admission

Observation may be done in-hospital or in the emergency department, depending on local routines. We recommend checking for decreased level of consciousness (GCS) and unilateral extremity weakness (basic neurological exam) every 15 minutes during the first 3 hours post-trauma and every 30 minutes following this. Observation frequency can be tailored to patient status (i.e. stable patients may need less frequent observation whilst unstable (worrying and/or worsening signs/symptoms) may need intensified observation). The recommended minimum observation time is 6 hours.

Some patients with minimal head injury or normal CT or S100B <0.10 µg/L following mild head injury may need admission for other reasons than the head injury (such as elderly patients without sufficient help at home, patients with other injuries, intoxicated patients, or patients requiring additional assessment for the underlying cause of trauma such as syncope). Since these patients have a very low risk of intracranial complications, they do not need the extensive observation routine mentioned above.

CT

A non-contrast CT in compliance with local radiology routines should be performed as quickly as possible, with a greater urgency for more severe clinical presentations in accordance with the guideline. The patient should be adequately monitored when waiting for the CT scan. When CT is abnormal, consider contacting a neurosurgeon or neurotrauma center for advice concerning further management. Symptomatic patients with a normal non-contrast head CT may need additional radiology (such as CT angiography for possible traumatic vascular dissections).

Repeat CT

Routine repeat CT is not recommended. However, a repeat CT should be done immediately in patients with deterioration in GCS score (≥2 points) or new/progressive neurological deficits (a new, persisting post-traumatic unilateral motor or sensory dysfunction, dysphasia or visual disturbance).

Discharge

ALL patients with head injury should receive oral and written instructions upon discharge (see separate form). These provide general advice concerning their head injury. Do not discharge a patient with worrying or worsening symptoms (other than typical post-injury issues such as mild headache and nausea). Patients should be discharged at their premorbid neurological and functional baseline and into an appropriate level of supervision or care.

Scandinavian Guidelines for Initial Management of Adult Patients with Minimal, Mild and Moderate Head Injury Help Sheet (GFAP + UCH-L1 Available)

Main Points

This guideline is based upon evidence-derived recommendations and expert consensus from the Scandinavian Neurotrauma Committee (SNC) mTBI working group. Being a guideline, it should not override clinical judgement and may be superseded if necessary. Glasgow Coma Scale (GCS) scores should be noted as the score after resuscitation and reduced GCS scores must be reductions from baseline. A standard clinical exam should be sufficiently comprehensive for the various risk factors to be adequately assessed. This guideline is intended for patients who are candidates for emergency neurosurgical or medical interventions. If the patient is suffering from severe comorbidities and is assessed not to be appropriate for acute neurosurgical intervention, or withdraws consent for further investigation, we do NOT recommend the application of this guideline. The guideline does not apply automatically to every reported contact with the head. Before applying the risk factors, the assessing clinician should determine that the mechanism, symptoms, or uncertainty surrounding the event provides reasonable grounds to suspect a clinically relevant head injury.

Mild - High Risk

These patients have GCS scores 14-15 and have had a post-traumatic seizure, have focal neurological deficits (a new, persisting post-traumatic unilateral motor or sensory dysfunction, dysphasia or visual disturbance), or have clinical signs of a depressed skull fracture (palpable depression or abnormal discontinuity of skull) or a basal skull fracture (raccoon eyes, Battle's sign, haemotympanum, cerebrospinal fluid rhinorrhea). These patients are relatively uncommon but have a relatively high risk of intracranial complications. They should undergo a head computed tomography (CT) and also be admitted for close observation for at least 12 hours even if the CT is normal.

Mild - Medium Risk

These patients have GCS score 14-15 and have an intraventricular shunt or are on dual antiplatelet therapy (DAPT). These patients should undergo a head CT scan and if the CT is normal, can be discharged if symptoms have been stable or improving.

Mild - Low Risk

These patients have GCS score 14 without risk factors or alternatively have GCS score 15 and have at least two of the following: anticoagulation use, antiplatelet use, suspected or confirmed loss of consciousness (i.e. the patient cannot clearly deny loss of consciousness), repeated vomiting, a scalp wound, or age ≥65. If less than 12 hours have passed since the trauma and the patient is <65 years of age, sample serum GFAP and UCH-L1. If GFAP is ≤22 pg/mL and UCH-L1 is ≤327 pg/mL (thresholds may vary depending on the assay), the patient has a very low risk of serious intracranial complications and can be discharged. If either GFAP or UCH-L1 is above their respective thresholds, either observation (see below) or a CT scan should be performed. If the CT is normal, the patient can be discharged if symptoms have been stable or improving.

Minimal

These patients have GCS score 15 and have no loss of consciousness, repeated vomiting, or any medium or high risk factors. They have a very low risk of intracranial complications and can be discharged from the hospital without a CT scan or observation.

Observation and Admission

Observation may be done in-hospital or in the emergency department, depending on local routines. We recommend checking for decreased level of consciousness (GCS) and unilateral extremity weakness (basic neurological exam) every 15 minutes during the first 3 hours post-trauma and every 30 minutes following this. Observation frequency can be tailored to patient status (i.e. stable patients may need less frequent observation whilst unstable (worrying and/or worsening signs/symptoms) may need intensified observation). The recommended minimum observation time is 6 hours.

Some patients with minimal head injury or normal CT or GFAP ≤22 pg/mL and UCH-L1 ≤327 pg/mL following mild head injury may need admission for other reasons than the head injury (such as elderly patients without sufficient help at home, patients with other injuries, intoxicated patients, or patients requiring additional assessment for the underlying cause of trauma such as syncope). Since these patients have a very low risk of intracranial complications, they do not need the extensive observation routine mentioned above.

CT

A non-contrast CT in compliance with local radiology routines should be performed as quickly as possible, with a greater urgency for more severe clinical presentations in accordance with the guideline. The patient should be adequately monitored when waiting for the CT scan. When CT is abnormal, consider contacting a neurosurgeon or neurotrauma center for advice concerning further management. Symptomatic patients with a normal non-contrast head CT may need additional radiology (such as CT angiography for possible traumatic vascular dissections).

Repeat CT

Routine repeat CT is not recommended. However, a repeat CT should be done immediately in patients with deterioration in GCS score (≥2 points) or new/progressive neurological deficits (a new, persisting post-traumatic unilateral motor or sensory dysfunction, dysphasia or visual disturbance).

Discharge

ALL patients with head injury should receive oral and written instructions upon discharge (see separate form). These provide general advice concerning their head injury. Do not discharge a patient with worrying or worsening symptoms (other than typical post-injury issues such as mild headache and nausea). Patients should be discharged at their premorbid neurological and functional baseline and into an appropriate level of supervision or care.

Scandinavian Guidelines for Initial Management of Adult Patients with Minimal, Mild and Moderate Head Injury Help Sheet (GFAP Available)

Main Points

This guideline is based upon evidence-derived recommendations and expert consensus from the Scandinavian Neurotrauma Committee (SNC) mTBI working group. Being a guideline, it should not override clinical judgement and may be superseded if necessary. Glasgow Coma Scale (GCS) scores should be noted as the score after resuscitation and reduced GCS scores must be reductions from baseline. A standard clinical exam should be sufficiently comprehensive for the various risk factors to be adequately assessed. This guideline is intended for patients who are candidates for emergency neurosurgical or medical interventions. If the patient is suffering from severe comorbidities and is assessed not to be appropriate for acute neurosurgical intervention, or withdraws consent for further investigation, we do NOT recommend the application of this guideline. The guideline does not apply automatically to every reported contact with the head. Before applying the risk factors, the assessing clinician should determine that the mechanism, symptoms, or uncertainty surrounding the event provides reasonable grounds to suspect a clinically relevant head injury.

Mild - High Risk

These patients have GCS scores 14-15 and have had a post-traumatic seizure, have focal neurological deficits (a new, persisting post-traumatic unilateral motor or sensory dysfunction, dysphasia or visual disturbance), or have clinical signs of a depressed skull fracture (palpable depression or abnormal discontinuity of skull) or a basal skull fracture (raccoon eyes, Battle's sign, haemotympanum, cerebrospinal fluid rhinorrhea). These patients are relatively uncommon but have a relatively high risk of intracranial complications. They should undergo a head computed tomography (CT) and also be admitted for close observation for at least 12 hours even if the CT is normal.

Mild - Medium Risk

These patients have GCS scores 14-15 and have an intraventricular shunt or are on dual antiplatelet therapy (DAPT). These patients should undergo a head CT scan and if the CT is normal, can be discharged if symptoms have been stable or improving.

Mild - Low Risk

These patients have GCS score 14 without risk factors or alternatively have GCS score 15 and have at least two of the following: anticoagulation use, antiplatelet use, suspected or confirmed loss of consciousness (i.e. the patient cannot clearly deny loss of consciousness), repeated vomiting, a scalp wound, or age ≥65. If less than 12 hours have passed since the trauma and the patient is <65 years of age, sample serum GFAP. If GFAP is ≤22 pg/mL (the threshold may vary depending on the assay), the patient has a very low risk of serious intracranial complications and can be discharged. If GFAP is above the threshold, either observation (see below) or a CT scan should be performed. If the CT is normal, the patient can be discharged if symptoms have been stable or improving.

Minimal

These patients have GCS score 15 and have no loss of consciousness, repeated vomiting, or any medium or high risk factors. They have a very low risk of intracranial complications and can be discharged from the hospital without a CT scan or observation.

Observation and Admission

Observation may be done in-hospital or in the emergency department, depending on local routines. We recommend checking for decreased level of consciousness (GCS) and unilateral extremity weakness (basic neurological exam) every 15 minutes during the first 3 hours post-trauma and every 30 minutes following this. Observation frequency can be tailored to patient status (i.e. stable patients may need less frequent observation whilst unstable (worrying and/or worsening signs/symptoms) may need intensified observation). The recommended minimum observation time is 6 hours.

Some patients with minimal head injury or normal CT or GFAP ≤22 pg/mL following mild head injury may need admission for other reasons than the head injury (such as elderly patients without sufficient help at home, patients with other injuries, intoxicated patients, or patients requiring additional assessment for the underlying cause of trauma such as syncope). Since these patients have a very low risk of intracranial complications, they do not need the extensive observation routine mentioned above.

CT

A non-contrast CT in compliance with local radiology routines should be performed as quickly as possible, with a greater urgency for more severe clinical presentations in accordance with the guideline. The patient should be adequately monitored when waiting for the CT scan. When CT is abnormal, consider contacting a neurosurgeon or neurotrauma center for advice concerning further management. Symptomatic patients with a normal non-contrast head CT may need additional radiology (such as CT angiography for possible traumatic vascular dissections).

Repeat CT

Routine repeat CT is not recommended. However, a repeat CT should be done immediately in patients with deterioration in GCS score (≥2 points) or new/progressive neurological deficits (a new, persisting post-traumatic unilateral motor or sensory dysfunction, dysphasia or visual disturbance).

Discharge

ALL patients with head injury should receive oral and written instructions upon discharge (see separate form). These provide general advice concerning their head injury. Do not discharge a patient with worrying or worsening symptoms (other than typical post-injury issues such as mild headache and nausea). Patients should be discharged at their premorbid neurological and functional baseline and into an appropriate level of supervision or care.

Discharge advice for a person who has had a head injury

A doctor has carefully checked you after your head injury and it is now safe for you to leave the hospital.

Contact emergency medical services if alarming symptoms occur such as increasing drowsiness or difficulty waking, severe headache, repeated vomiting, seizure, new weakness or numbness, speech or visual disturbance, impaired coordination, or any other clear neurological deterioration.

Things you shouldn't worry about

You may feel some symptoms over the next few days that should go away in the next 4 weeks. These include a mild headache, problems concentrating, memory issues (forgetfulness), feeling sick (without vomiting), dizziness, feeling irritated (having a bad temper), abnormal tiredness or problems sleeping. If these symptoms get worse, persist for more than 4 weeks, or if you feel very concerned about them, you should see a healthcare provider. We also recommend that you see your family doctor about your ability to drive a motor vehicle if you experience any of these problems.

If you have remaining symptoms

- **DO NOT** take any alcohol or drugs.
- **DO NOT** take sleeping pills, sedatives or tranquilisers unless approved by a doctor.
- Avoid excessive mental or physical exertion for the first 48 hours after injury. Complete bedrest after this is not beneficial. Some symptoms may get worse during physical exertion, but this is not dangerous. See your local concussion guidelines for specific return-to-sports guidance.

Long-term problems

Most people recover and do not have any long-term problems. However, some people may experience prolonged symptoms, even after weeks or months. If you have symptoms that impact your quality of life after 4 weeks, please contact your family doctor so that he or she can check to make sure you are recovering properly.

Contact information:
